# Specifically Breaking Through the Injured Blood‐Brain Barrier With Tannic Acid‐Based Nanomedicine for Ischemic Stroke Ischemia Reperfusion Treatment

**DOI:** 10.1002/EXP.20240388

**Published:** 2025-09-09

**Authors:** Xiaojing Shi, Shuya Wang, Tingli Xiong, Ruishi Li, Wenxuan Zheng, Wensheng Chen, Tianjiao Zhao, Yongqi Yang, Xiaohong Ying, Weimin Qi, Yingci Xia, Jue Wang, Yuqi Zhang, Qiong Huang, Yayun Nan, Kelong Ai

**Affiliations:** ^1^ Department of Pharmacy Xiangya Hospital Central South University Changsha China; ^2^ Xiangya School of Pharmaceutical Sciences Central South University Changsha China; ^3^ National Clinical Research Center for Geriatric Disorders Xiangya Hospital Central South University Changsha China; ^4^ Communication Design Royal Melbourne Institute of Technology University Melbourne Victoria Australia; ^5^ Geriatric Medical Center People's Hospital of Ningxia Hui Autonomous Region Yinchuan Ningxia China; ^6^ Hunan Provincial Key Laboratory of Cardiovascular Research Xiangya School of Pharmaceutical Sciences Central South University Changsha China; ^7^ Key Laboratory of Aging‐Related Bone and Joint Diseases Prevention and Treatment Ministry of Education Xiangya Hospital Central South University Changsha China; ^8^ FuRong Laboratory Changsha Hunan China

**Keywords:** endoplasmic reticulum stress, inflammation, ischemic stroke ischemia reperfusion, mitochondrial targeting, oxidative stress

## Abstract

Protecting neuronal mitochondria by eliminating the mitochondrial ROS (mtROS) storm is crucial to abrogate the neuronal damage cascade of ischemic stroke ischemia reperfusion (ISIR), which is a long‐standing challenge in the field of ischemic stroke (IS). Existing blood‐brain barrier (BBB) penetration methods are usually unable to distinguish between healthy brain tissue and cerebral infarction tissue, and BBB targeting is not compatible with mitochondrial targeting, resulting in a huge barrier to the specific elimination of mtROS in neuronal mitochondria in ISIR. This study introduces an elegantly designed tannic acid, polydopamine, and Mo‐based heteropolyacid ternary composite nanomedicine (TPM), which not only has a superb ability to eliminate multiple ROS thanks to the introduction of polydopamine, but also can actively recognize the injured BBB site, specifically enter the neurons in the cerebral infarction area, and then highly specifically target the mitochondria of neurons to efficiently eliminate mtROS. TPM could significantly inhibit neuronal apoptosis by protecting mitochondria and eliminate inflammation by inhibiting activation of the STING pathway, thereby significantly reducing the size of cerebral infarction. This sequential targeting of TPM from the injured BBB to neuronal mitochondria provides a promising strategy to treat ISIR in the clinical setting.

## Graphical abstract

Protecting neuronal mitochondria by eliminating the mitochondrial ROS (mtROS) storm is crucial to abrogate the neuronal damage cascade of ischemic stroke ischemia reperfusion (ISIR), which is a long‐standing challenge in the field of ischemic stroke (IS). Existing blood‐brain barrier (BBB) penetration methods are usually unable to distinguish between healthy brain tissue and cerebral infarction tissue, and BBB targeting is not compatible with mitochondrial targeting, resulting in the huge barrier of specific elimination of mtROS in neuronal mitochondria in ISIR. This study introduces an elegantly designed tannic acid, polydopamine, and Mo‐based heteropolyacid ternary composite nanomedicine (TPM), which not only has a superb ability to eliminate multiple ROS thanks to the introduction of polydopamine, but also can actively recognize the injured BBB site, specifically enter the neurons in the cerebral infarction area, and then highly specifically target the mitochondria of neurons to efficiently eliminate mtROS. TPM could significantly inhibit neuronal apoptosis by protecting mitochondria and eliminate inflammation by inhibiting activation of the STING pathway, thereby significantly reducing the size of cerebral infarction. This sequential targeting of TPM from the injured BBB to neuronal mitochondria provides a promising strategy to treat ISIR in the clinical setting.

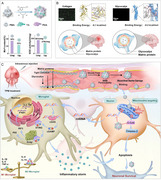

## 1 Introduction

Ischemic stroke (IS) is a disease caused by blockage of blood vessels in the brain [1]. IS causes 11.6% of deaths worldwide and is the second leading cause of death and disability worldwide [2]. The treatment of IS mainly relies on revascularization for the infarcted area [3]. With technological advances, great progress has been made in reperfusion therapy for IS, and the treatment window can even be extended to 24 h for revascularization [4]. Despite complete recanalization of the infarcted blood vessels, more than 60% of the survivors remain disabled, which is mainly attributed to ischemic stroke ischemia reperfusion (ISIR) injury after blood flow restoration [5]. ISIR damages all cell types of the neurovascular unit within the ischemic area, including neurons, microglia, pericytes, and endothelial cells [6]. Among these cells, neurons may be the most vulnerable, and their death is the most important factor contributing to IS‐related mortality and disability [7]. Neurons are abundant in mitochondria because of their extreme demand for energy [8]. In ISIR, electron leakage from the mitochondrial electron transport chain of neurons leads to mitochondrial ROS (mtROS) burst [9]. The mtROS directly destroy the mitochondrial membrane to trigger cytochrome c‐induced neuronal cell death [10]. Damaged mitochondria further release a large amount of mitochondrial DNA (mtDNA) to activate the microglial STING inflammatory pathway, ultimately leading to an inflammatory storm to trigger the neuronal damage cascade of ISIR [11]. Currently, many neuroprotective agents (such as antioxidant‐edaravone, anti‐inflammatory drug‐idebenone, and neuroprotective agent‐nerinetide) and emerging mitochondrial protective drugs (such as S321 and cyclosporine A) are being developed to treat ISIR and restore mitochondrial structure and function [12]. In addition, emerging nanodrugs are being developed to improve the bioavailability of loaded drugs in ISIR lesions, such as neutrophil membrane‐camouflaged polyprodrug nanomedicine [13], and bacteria‐derived outer‐membrane vesicles [14]. The blood‐brain barrier (BBB) and limited mitochondrial targeting greatly restrict the bioavailability of these drugs, posing a huge challenge to their clinical application [15]. Although a number of strategies for penetrating the BBB have been developed, these methods cannot distinguish between infarcted tissues and healthy brain tissues. Therefore, a strategy of actively targeting the BBB site of infarct tissues and then targeting neuronal mitochondria is urgently needed to significantly improve the bioavailability of drugs and reduce side effects.

The BBB is composed of capillary endothelial cells, pericytes, and astrocyte end feet, which ultimately form a dense matrix layer rich in type III collagen in the capillary vessels [16]. The surface of the capillary matrix layer is covered with a dense glycocalyx structure [17]. During ISIR development, ROS and multiple inflammatory factors (TNF‐α, IL‐1β) lead to the activation of proteases such as matrix metalloproteinases (MMPs) [18]. The activation of these MMPs, especially MMP9, can directly degrade tight junction proteins [19], damage the BBB, and form endothelial gaps. They also cause the dense glycocalyx structure to be stripped away, exposing a matrix rich in type III collagen [20]. On the other hand, mitochondrial targeting groups are generally molecules with high affinity for biological molecules of the mitochondrial outer membrane, such as triphenylphosphine, S321, and so on [21]. However, these groups usually have strong positive charges, and drugs modified with these groups cannot pass through the circulatory system to reach the damaged BBB [22]. We noticed that both type III collagen and mitochondrial outer membrane protein TOM20 are rich in proline residues. Tannic acid (TA), a plant polyphenol, causes the astringent taste sensation due to its strong binding to the proline‐rich mucins [23]. Therefore, we hypothesized that TA‐modified drugs may provide a potential approach to achieve an active targeting strategy from the injured BBB to neuronal mitochondria.

**SCHEME 1:**
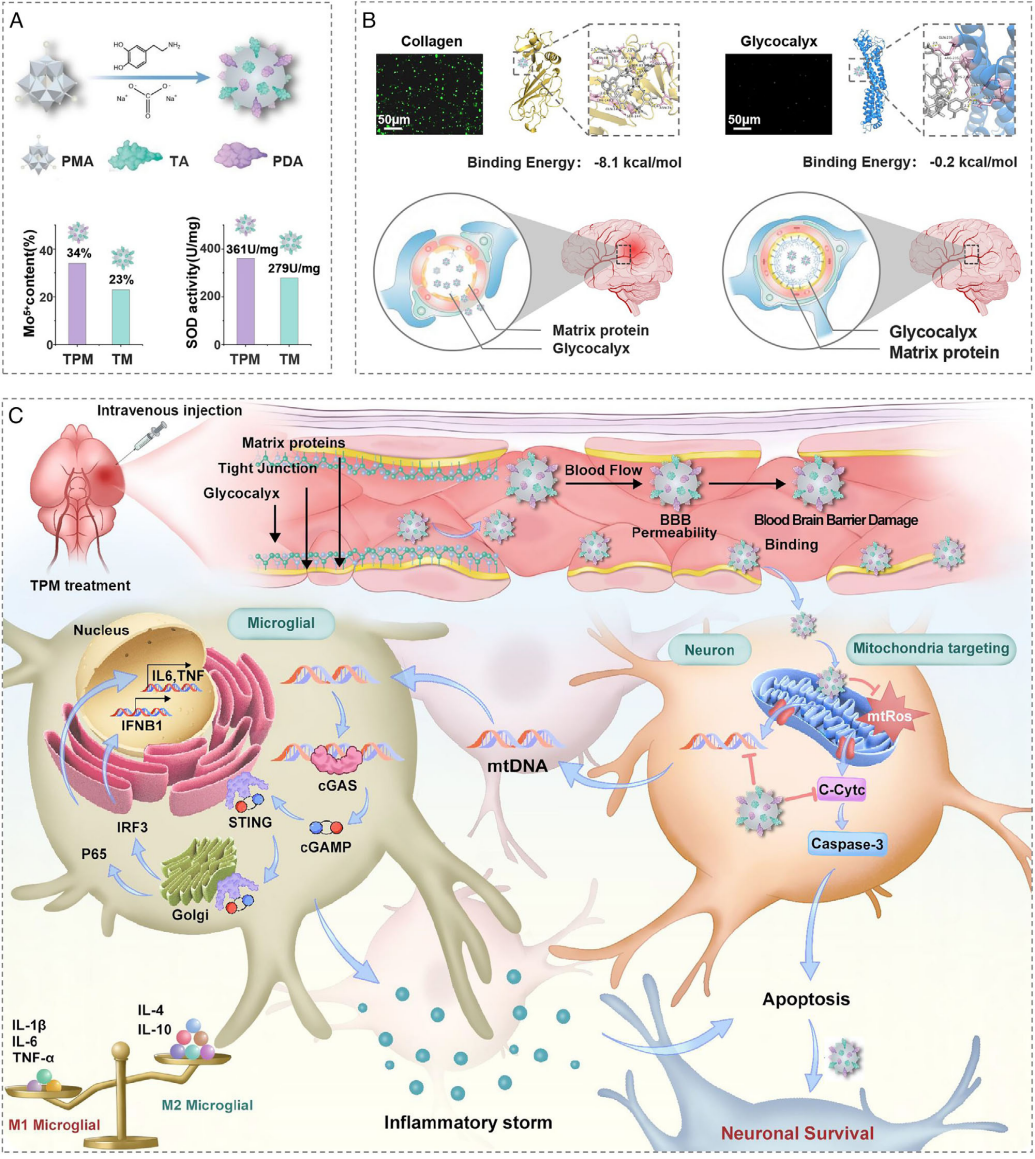
Mechanism diagram of TPM in treating ISIR. (A) TPM is reduced from phosphomolybdic acid (PMA) in the presence of dopamine and TA under alkaline conditions and has a strong ROS scavenging ability. (B) TPM has a high affinity for type III collagen and TOM20, and can successfully pass through the damaged blood‐brain barrier. (C) Specifically, after intravenous injection of TPM, due to its high affinity for damaged vascular matrix proteins and mitochondrial membrane protein TOM20, it targets neuronal mitochondria, effectively clears mtROS, and inhibits neuronal apoptosis. At the same time, TPM could effectively reduce the release of mtDNA in neurons, reduce the activation of STING signals in microglia, and eliminate pro‐inflammatory cytokine storms. Ultimately, TPM effectively reduces neuronal death by reducing oxidative stress and eliminating neuroinflammation.

In this study, we found for the first time that TA has a high affinity for type III collagen and TOM20, while its binding affinity to the glycocalyx group and serum albumin is negligible. On this basis, we developed a highly effective treatment for IS through a tailored ternary composite antioxidant nanomedicine based on TA, polydopamine (PDA), and mo‐heteropolyacid (TPM), which could actively target the injured BBB to neuronal mitochondria in sequence. Given the potent ROS burst in the mitochondria of ISIR neurons, we further enhanced the ROS‐eliminating ability of TPM by PDA [24] (Scheme 1A). Moreover, TPM has a high affinity for type III collagen and TOM20 due to the modification of TA (Scheme 1B). After intravenous injection, TPM accumulated specifically at the injured BBB and entered the neuronal cells in the affected brain tissue through the gaps in the injured BBB. Afterwards, TPM is further bound to TOM20 to specifically eliminate mtROS on mitochondria, restore mitochondrial function, relieve endoplasmic reticulum (ER) stress, and significantly inhibit neuronal cell death. Moreover, the release of mtDNA is also significantly inhibited, subsequently significantly reducing the activation of the STING pathway in microglia and alleviating neuroinflammation (Scheme 1C). Thanks to the advantages of sequential targeting from damaged BBB to neuronal mitochondria, TPM exhibited a strong therapeutic effect and could effectively reverse ISIR even at an ultra‐low dose (3 mg kg^−1^). In addition, dopamine is a common component in the human body, and TPM still had excellent biocompatibility and biosafety even at 10 times the therapeutic dose. Therefore, TPM has broad clinical application prospects in ISIR.

## 2 Results

### 2.1 Characterization of TPM

**FIGURE 1:**
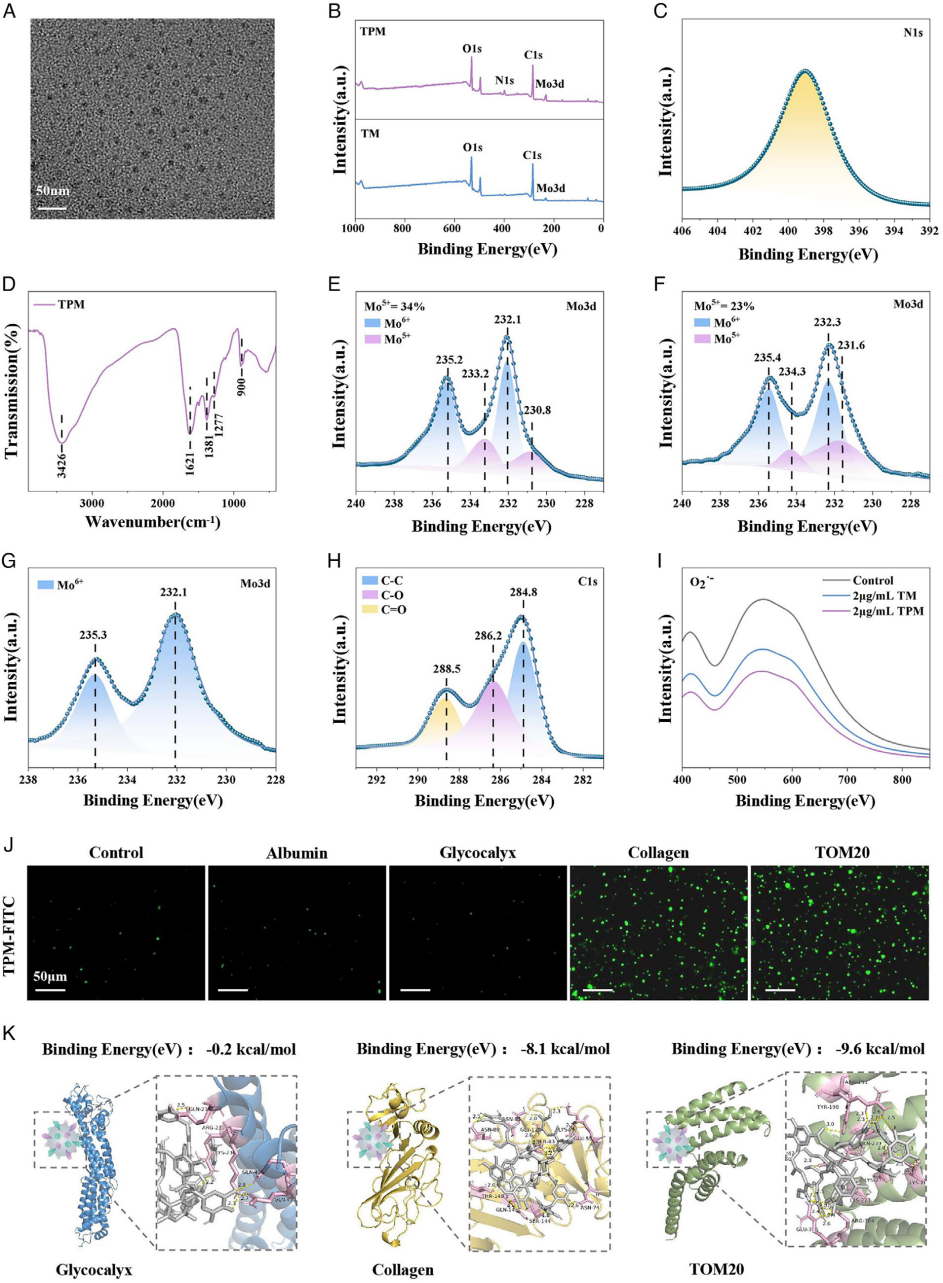
Characterization of TPM. (A) TEM visualization of TPM, scale bar: 50 nm. (B) Total spectrum energy of TPM and TM. (C) N1s narrow scan XPS spectrum of TPM. (D) FTIR spectrum of TPM. (E) Mo3d narrow scan XPS spectrum of TPM. (F) Mo3d narrow scan XPS spectrum of TM. (G) Mo3d narrow scan XPS spectrum of TPM after reaction with H_2_O_2_. (H) C1s narrow scan XPS spectrum of TPM after reaction with H_2_O_2_. (I) O_2_^•−^ scavenging ability of TPM and TM. (J) The protein‐coated plate experiment, scale bar: 50 µm. (K) Results of the molecules docking of TA with glycocalyx, collagen protein, and TOM20 protein.

TPM was prepared by the reduction of PMA with dopamine and TA under alkaline conditions. In this process, dopamine was oxidized and polymerized into PDA, while Mo^6+^ was partially reduced to Mo^5+^. Transmission electron microscopy (TEM) analysis showed that TPM was well dispersed in an aqueous system, and its hydrodynamic diameter was 10–15 nm (Figure 1A and Figure S1). As expected, the TPM surface was negatively charged (−25.2 mV) due to TA and PDA modification (Figure S2). Through X‐ray photoelectron spectroscopy (XPS), TPM was composed of four elements: O, N, C, and Mo, while TM without PDA modification only contained O, C, and Mo (Figure 1B). Further, the fine N1s peak of XPS showed that the N element in TPM was mainly pyrrolidone N (binding energy of 400.0 eV), which fully demonstrated that PDA was successfully modified onto TPM (Figure 1C). Through Fourier transform infrared spectroscopy (FTIR), the characteristic peaks of hydroxyl (3426 cm^−1^), carbonyl (1621 cm^−1^), benzene ring (1381 cm^−1^) and phenolic hydroxyl (1277 cm^−1^) were observed in TPM, indicating that TA was successfully modified on TPM (Figure 1D and Figure S3). Through UV–vis–NIR spectroscopy, TPM showed characteristic absorption peaks extending from visible light to the near‐infrared region (Figure S4) due to the charge transfer between Mo^5+^ and Mo^6+^ through the bridging oxygen bond, suggesting that TPM contains a higher proportion of Mo^5+^. Through the XPS fine peak of Mo3d, the Mo element in TPM was mainly manifested as Mo^5+^ (binding energy 233.2 and 230.8 eV) and Mo^6+^ (binding energy 235.2 and 232.1 eV), among which the content of Mo^5+^ was as high as 34% (Figure 1E). In sharp contrast, the Mo^5+^ content in TM was only 23% (Figure 1F), indicating that PDA modification could increase the Mo^5+^ content in TPM (Figures S5 and S6). The Mo^5+^ content determined the ability of TPM to scavenge ROS. TPM was analyzed by XPS after reaction with various ROS (H_2_O_2_, **·**OH, O_2_^•−^, and ONOO^−^). After reacting with H_2_O_2_, Mo^5+^ of TPM was converted into Mo^6+^(Figure 1G), while the peak spectra of C, N, and O did not change (Figure 1H and Figure S7). Similar results were also found after the reaction of TPM with **·**OH (Figure S8), O_2_^•−^ (Figure S9), and ONOO^−^ (Figure S10), fully confirming that TPM eliminated ROS through Mo^6+^/Mo^5+^ redox. As expected, TPM could efficiently scavenge O_2_^•−^ by the nitroblue tetrazolium (NBT) method, with a pseudo‐superoxide dismutase (SOD) activity of approximately 361 U mg^−1^, which was 1.3 times that of TM (279 U mg^−1^) (Figure 1I and Figure S11). Similarly, TPM could also effectively eliminate **·**OH (Figure S12), ONOO^−^ (Figure S13), and H_2_O_2_ (Figure S14), and the effects were better than TM. Finally, the binding ability of TPM to type III collagen and TOM20 was further verified, and glycogen and serum protein were used as controls. TPM was first labeled with fluorescein isothiocyanate (FITC) (Figure S15), and then serum albumin, glycocalyx, type III collagen, and TOM20 were coated on plates and soaked in TPM‐FITC solution. As shown in Figure 1J and Figure S16, TPM could bind to the plates coated with type III collagen and TOM20, and the fluorescence intensity was nearly 40 times that of the plates coated with serum albumin and glycocalyx. Next, the binding energy of TA with serum albumin, glycocalyx, type III collagen, and TOM20 was calculated by Pyrx Vina. The binding energy of TA to glycocalyx and serum albumin was weak, both −0.2 kcal mol^−1^, while the binding energy to TOM20 and type III collagen could be as high as −9.6 and −8.1 kcal mol^−1^, respectively (Figure 1K and Figure S17). The above results fully demonstrated the successful preparation of TPM, and the introduction of PDA greatly improved the ability of TPM to eliminate ROS, and the modification of TA enabled TPM to bind to type III collagen and TOM20.

### 2.2 TPM Targets Damaged Brain Tissue and Improves Treatment of ISIR

**FIGURE 2:**
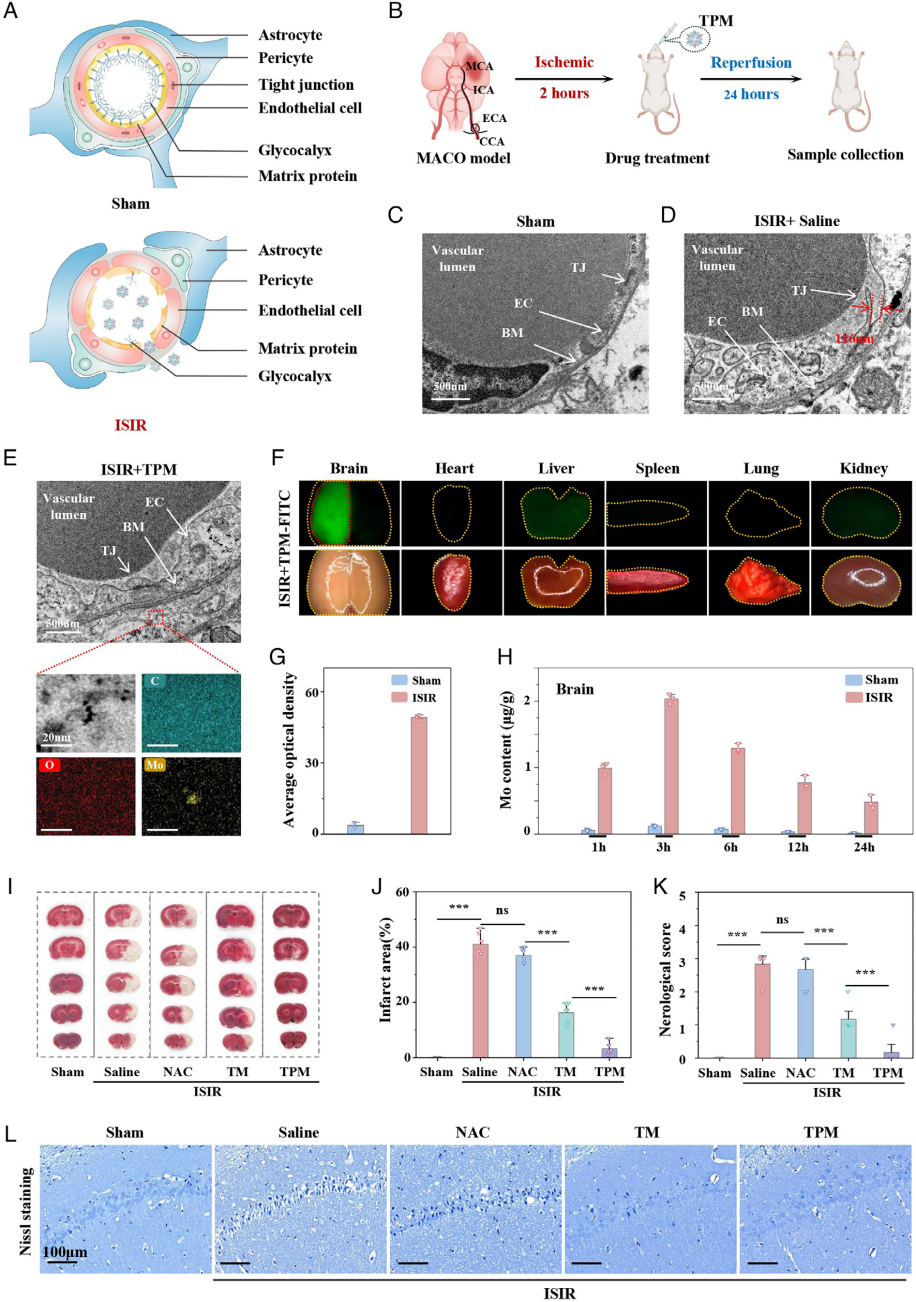
Brain targeting of TPM and improvement of ISIR. (A) Schematic diagram of BBB injury induced by ISIR. (B) Schematic diagram of TPM treating ISIR in rats. (C,D) TEM images of blood vessels in normal and ISIR rat brain tissue, scale bar: 500 nm. (E) TEM and HAADF‐TEM of TPM in the cerebral infarct area (red dotted circle marks TPM), scale bar: 500 nm. (F) Representative images of TPM‐FITC fluorescence imaging and bright field in the brain, heart, lung, liver, spleen, and kidney of rats with ISIR. (G) Fluorescence imaging statistics of the brain organ. (H) ICP‐MS measured the content of Mo in brain tissue after intravenous injection of TPM at 1, 3, 6, 12, and 24 h. (I,J) Brain tissue TTC staining (I) and infarct size statistics (J). (K) Neurological score. (L) Nissl staining of brain tissue in different groups of rats. Scale bar: 100 µm. (In vivo: *n* ≥ 3 animals per group. In vitro: *n* = 3 independent experiments, ns: *P* > 0.05, ^***^ *p* < 0.001).

In ISIR, the damaged BBB provides an opportunity for TPM to enter the injured brain tissue due to the loss of glycosylation and exposure to type III collagen (Figure 2A). The middle cerebral artery occlusion (MCAO) was used to simulate the cerebral ischemia stage. After 2 h of ischemia, the suture blocking the middle cerebral artery was removed to restore blood circulation and simulate the reperfusion stage, thus establishing a rat ISIR model (Figure 2B). First, TEM was used to observe the ultrastructure of brain capillaries. As shown in Figure 2C,D, the morphology of brain capillaries in the Sham group was intact, and the endothelial cells were tightly connected. In the ISIR group, the endothelial cells and vascular basement membrane were obviously swollen, and the endothelial cells lost their tight junctions to form gaps of different sizes. Further statistics showed that the average gap was about 117.85 nm (Figure S18), which was much larger than the particle size of TPM 10–15 nm, providing the possibility of TPM enrichment in ISIR tissue. As expected, we found TPM widely distributed in the capillaries of ISIR tissue in the TPM group through TEM and confirmed that these particles were TPM by high‐angle annular dark field (HAADF) scanning TEM (STEM) (Figure 2E), providing the direct evidence for the specific targeting of TPM to infarcted brain tissue. Subsequently, TPM‐FITC was injected sublingually into ISIR rats at the onset of reperfusion to explore the distribution of TPM in vivo. TPM accumulation was observed in the injured brain tissue 1 h after sublingual intravenous injection of TPM‐FITC, while it was almost not distributed in the brain tissue of the Sham group and non‐infarct area (Figure 2F,G and Figure S19). Inductively coupled plasma‐mass spectrometry (ICP‐MS) was further used to detect the Mo content in various tissues and organs to evaluate the distribution dynamics of TPM in vivo. After intravenous injection, TPM rapidly accumulated in the injured brain tissue and reached a peak at 3 h (16.7 times that of the Sham group) (Figure 2H and Figure S20). In the Sham group, TPM was almost not distributed in the brain tissue but mainly in the liver and kidneys. In the ISIR group, the distribution percentage of TPM in the brain tissue reached about 45%, which fully confirmed the specific targeting ability of TPM to damaged brain tissue (Figure S21). In addition, the content of TPM in various tissues and organs began to slowly decrease at 6 h, which indicated that TPM had excellent biodegradability.

Subsequently, the effect of TPM was investigated in treating ISIR. We first screened the optimal dose of TPM for the treatment of ISIR by triphenyl tetrazolium chloride (TTC) staining. As shown in Figure S22, TPM at a dose of 0.75 mg kg^−1^ could effectively improve the cerebral infarction area of ISIR rats, and the therapeutic effect increases in a dose‐dependent manner. To further explore the therapeutic mechanism of TPM, we selected a dose of 3 mg kg^−1^ for follow‐up research, and selected TM and the clinically approved antioxidant drug N‐acetylcysteine (NAC) at the same dose as a control. Through TTC, the 3 mg kg^−1^ dose of TM group could reduce the cerebral infarction area of rats in the ISIR group from 41.01% to 16.26%, and the 3 mg kg^−1^ dose of TPM could reduce the infarction area to 3.14%, almost reversing the outcome of ISIR (Figure 2I,J). The improvement of cerebral infarction at the same dose of NAC was minimal because NAC did not target the infarcted brain tissue. Zea–Longa neurological function score is a key indicator for evaluating brain tissue neurological damage. The ISIR group and NAC group showed severe neurological damage (neurological score of approximately 3). In contrast, the neurological score in the TPM group was significantly reduced, and the neurological function score at a dose of 3 mg kg^−1^ was close to that of the Sham group, indicating that TPM treatment can significantly improve ISIR‐induced neurological damage (Figure 2K). Nissl staining was used to mark Nissl bodies in brain tissue sections to assess the damage of neurons in different treatment groups. Compared with the Sham group, the neurons in the ISIR group were necrotic, shrunken, and deformed. TPM significantly rescued the neurons in the hippocampus and restored the normal morphology of the neurons (Figure 2L). In addition, hematoxylin and eosin (HE) staining was performed to observe the morphology and pathological changes of brain tissue. Compared with the Sham group, the neurons in the cortical area of the brain tissue shrank and even dissolved, and a large number of vacuoles were visible in the ISIR group, while TPM treatment could greatly improve this situation (Figure S23). The above evidence proved that TPM could effectively target ISIR tissue and improve ISIR.

### 2.3 TPM Targets Neuronal Mitochondria and Restores Mitochondrial Function

**FIGURE 3:**
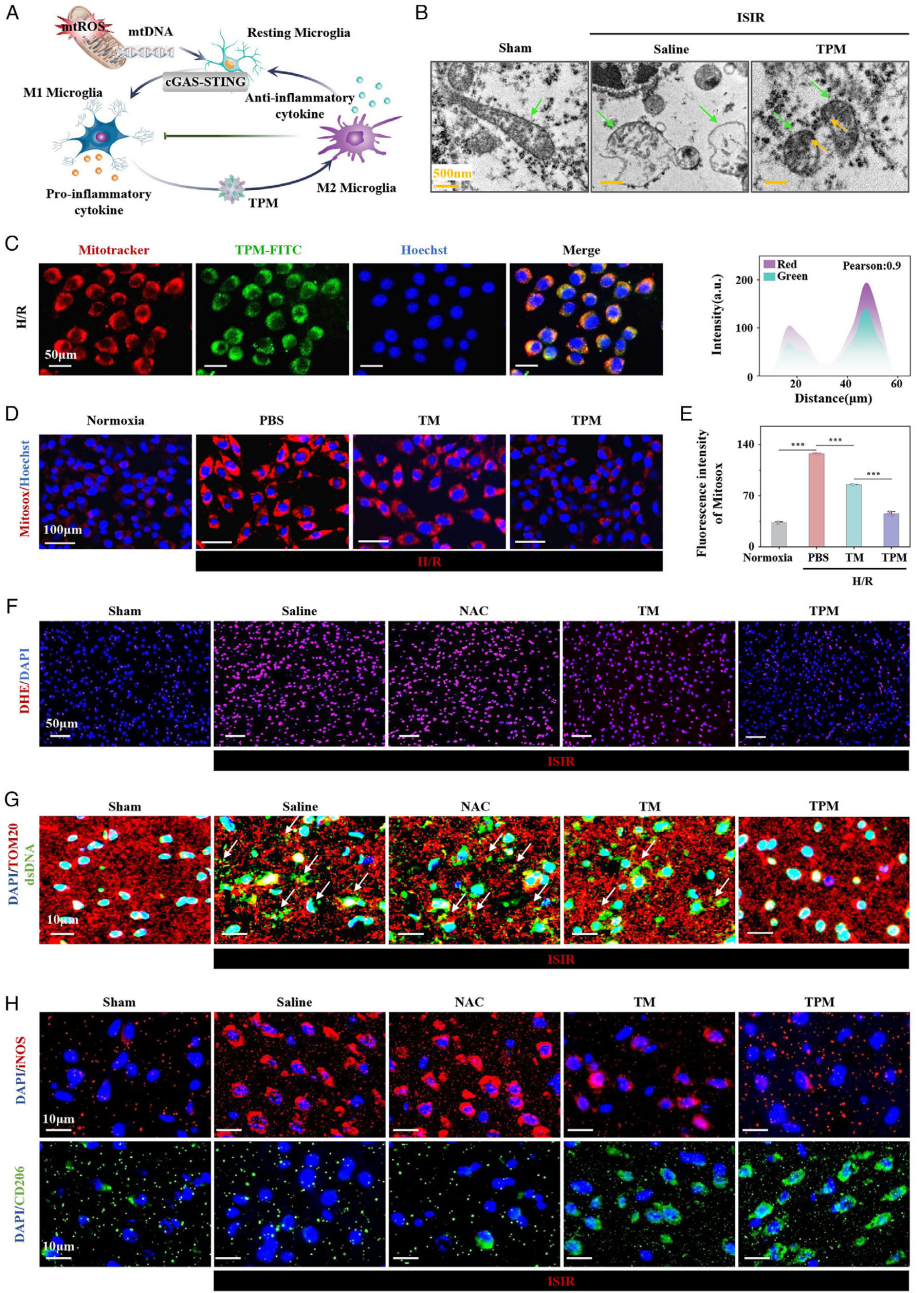
TPM targets neuronal mitochondria and restores mitochondrial function. (A) Schematic diagram of mitochondrial damage‐induced microglial activation. (B) TEM image of mitochondria (indicated by the green arrow) in brain tissue neurons from the Sham group, ISIR group, and TPM treatment group. Scale bar: 500 nm. (C) Representative images and co‐localization analysis of TPM and mitochondria, and Pearson coefficient analysis. Scale bar: 50 µm. (D,E) Immunofluorescence staining and quantification of mitochondrial superoxide in HT22 cells. Scale bar: 100 µm. (F) DHE staining images of brain tissue in each group. Scale bar: 50 µm. (G) Immunofluorescence staining of dsDNA and TOM20 in the cerebral infarction area of each treatment group. Scale bar: 10 µm. (H) Immunofluorescence staining of iNOS and CD206 in the cerebral infarction area of each treatment group. Scale bar: 10 µm. Data were expressed as mean ± SE. (*n* = 3, ^***^p < 0.001).

Mitochondrial damage is a core mechanism of neuronal death in ISIR. The targeting ability of TPM to mitochondria was further explored by labeling mitochondria with Mitotracker and incubation TPM‐FITC to track the distribution of TPM in HT22 cells. As shown in Figure 3C and Figure S24, TPM could be highly targeted to neuronal mitochondria both under normoxia and hypoxia/re‐oxygenation (H/R) conditions, the Pearson coefficients were 0.88 ± 0.05 and 0.90 ± 0.05, respectively, due to the high affinity of TA on the surface of TPM for TOM20. TPM could restore the viability of HT22 cells under H/R conditions (Figure S25). Subsequently, 2,7‐dichlorodihydrofluorescein diacetate (DCFH‐DA) probe was used to detect the level of ROS in HT22 cells. H/R induced an increase in the level of ROS in HT22 cells to 3.7 times that of the Normoxia group, while TPM treatment significantly reduced it to a level close to that of the normal group, NAC, as a ROS inhibitor, showed obvious antioxidant properties at a dose of 100 µg mL^−1^ while TPM could achieve similar antioxidant properties to NAC at a dose of 6 µg mL^−1^ (Figure S26). Flow cytometry experiments also confirmed that TPM could effectively eliminate H/R‐induced ROS in HT22 cells, and the effect was better than TM (Figure S27). The high level of ROS produced by H/R‐induced HT22 cells was mainly derived from mitochondria. Compared with the Normoxia group, the fluorescence intensity of MitoSOX in the H/R group was significantly enhanced (3.81 times that of the Normoxia group) through MitoSOX red probe, while TPM could significantly reduce the level of mtROS (Figure 3D,E) because of high mitochondrial targeting of TPM. In ISIR, mtROS damages the mitochondrial membrane, leading to a decrease in mitochondrial membrane potential (MMP). As shown in Figure S28 (fluorescence microscopy) and Figure S29 (flow cytometry), after H/R treatment, the MMP of HT22 cells was significantly decreased through JC‐1 probe, while TPM treatment could significantly restore the MMP. Mitochondrial function is further measured by ATP production. H/R causes a decrease in ATP synthase activity and a significant reduction in ATP production in HT22 cells, whereas treatment with TMP can restore ATP synthase activity and significantly increase ATP production to normal levels (Figures S30 and S31). These results indicated that TPM could effectively ameliorate H/R‐induced mitochondrial damage in HT22 cells.

In vivo, compared with the Sham group, the neuronal mitochondria in the ISIR group were severely damaged, showing swelling, vacuoles, partial rupture of the outer membrane, and dilation and rupture of the cristae through TEM. In the TPM group, TPM could be directly observed in the mitochondria of neurons (yellow arrows), and the morphology of mitochondria also returned to normal, indicating that TPM can target mitochondria and effectively alleviate mitochondrial damage (Figure 3B). The ROS levels in the infarct sites of different groups were further analyzed through DHE staining. Compared with the Sham group, the ROS level in the ISIR group was significantly increased (5.81 times that of the Sham group). TPM could effectively reduce the fluorescence intensity (1.37 times that of the Sham group), TM had a certain effect, and NAC had a negligible ability to scavenge ROS (Figure 3F and Figure S32). The mtROS destroys the lipids of the mitochondrial membrane and releases malondialdehyde (MDA). Therefore, the level of MDA reflects the damage to the mitochondrial membrane to a certain extent. TPM could significantly reduce the MDA level in the ISIR group, and the effect was better than that of TM (Figure S33). ISIR‐induced neuronal mitochondrial damage leads to the release of mtDNA from mitochondria. By DAPI/dsDNA/TOM20 co‐staining, in the ISIR group, a large amount of mtDNA was released into the cytoplasm and even outside the neurons because the mitochondria were severely damaged (Figure 3G). TPM had a protective effect on mitochondria and could significantly reduce the release of mtDNA. The effect of TPM was better than that of TM, while the effect of NAC was almost negligible. As a kind of damage‐associated molecular pattern molecule, mtDNA induces microglia to polarize toward M1(Figure 3A). Subsequently, we performed immunofluorescence staining of iNOS and CD206, marker proteins representing M1 and M2 glial cells, respectively, to determine the activation of microglia in cerebral infarction tissues of each group. After ISIR injury, almost all microglia in the ischemic area were activated into the pro‐inflammatory M1 type (CD206/iNOS was 0.13), while the microglia in the TPM group changed from M1 type to anti‐inflammatory M2 type (CD206/iNOS was 4.44) (Figure 3H and Figure S34). The above evidences fully prove that TPM could accumulate in the mitochondria of neuronal cells to protect mitochondria, and promote M2 polarization of microglia by reducing the release of mtDNA.

### 2.4 TPM Reverses Neuroinflammation

**FIGURE 4:**
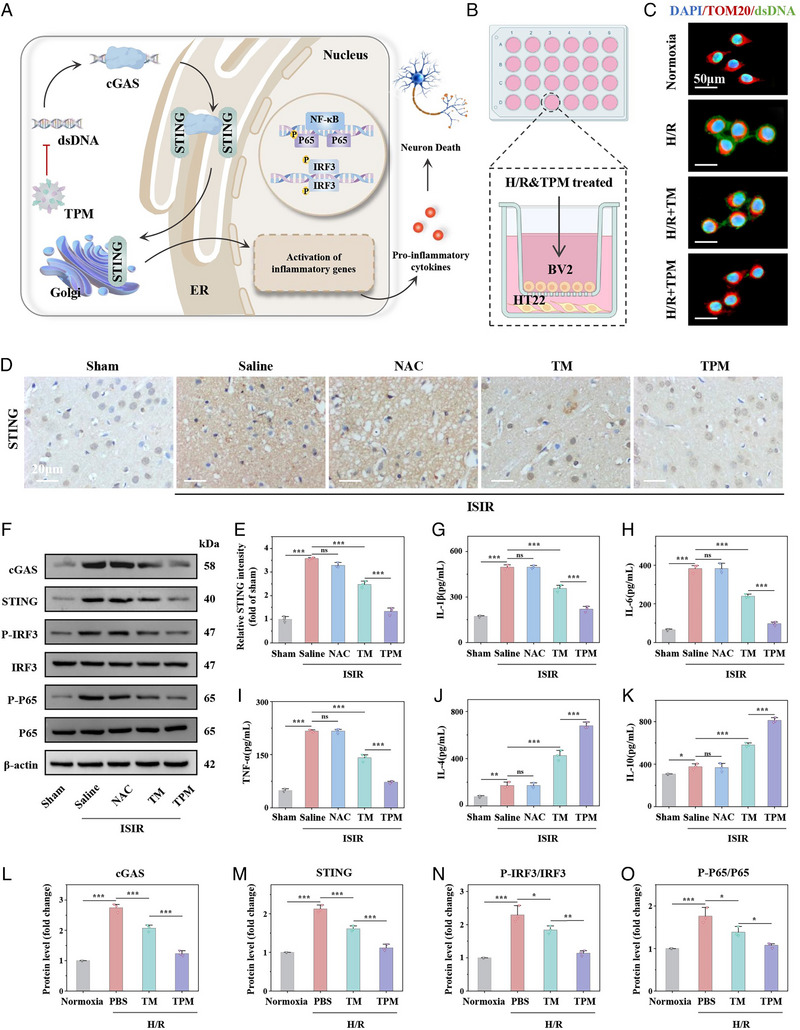
TPM reverses neuroinflammation induced by ISIR. (A) Mechanism of TPM inhibiting the activation of the microglial cGAS‐STING pathway. (B) Schematic illustration of the HT22 cells and BV2 cells coincubation experimental process. (C) Immunofluorescence staining of dsDNA of BV2 cells in different treatment groups. (D) Immunohistochemical staining of STING in the cerebral infarction area of each treatment group. Scale bar: 20 µm. (E) Quantitative analysis of STING immunohistochemical staining. (F) WB of inflammation‐related proteins in cerebral infarct area, including cGAS, STING, IRF3, P‐IRF3, P65, and P‐P65. (G–K) Determination of the levels of pro‐inflammatory factors (IL‐1β, IL‐6, and TNF‐α) and anti‐inflammatory factors (IL‐4 and IL‐10) in the cerebral infarction area of each treatment group. (L–O) Quantitative analysis of inflammation‐related proteins in BV2 cells, including cGAS (l), STING (m), P‐IRF3/ IRF3 (N), P‐P65/ P65 (O). Data were expressed as mean ± SE. (*n* = 3, ns, *p* > 0.05, ^*^p < 0.05, ^**^p < 0.01, ^***^p < 0.001).

Cyclic GMP‐AMP synthase (cGAS) in microglia is highly sensitive to the recognition and induction of mtDNA. The combination of cGAS and mtDNA activates its catalytic activity and produces 2′3′cyclic GMP‐AMP (cGAMP), which is a potent activator of STING. cGAMP binds to STING protein located in the ER and causes significant conformational changes, triggering oligomerization and translocation of STING to Golgi, promoting recruitment of interferon regulatory factor 3 (IRF3), and promoting phosphorylation of IRF3 to dimerize and translocate IRF3 to the nucleus. It can induce the expression of inflammatory mediators and pro‐apoptotic genes. Activation of STING also promotes the release and phosphorylation of NF‐κB, allowing it to undergo nuclear translocation, leading to the release of pro‐inflammatory cytokines such as IL‐6 (interleukin‐6) [25]. Promote the formation of an inflammatory microenvironment, and promote the polarization of microglia to M1 type, forming neuroinflammation and triggering the cascade of neuron damage of ISIR (Figure 4A). In vivo, immunohistochemistry staining was used to detect the expression of cGAS and STING in each group. Compared with the Sham group, the expression of cGAS and STING in the ISIR group was significantly increased, indicating that the cGAS‐STING signaling pathway was activated. TPM could significantly inhibit cGAS (Figure S35) and STING (Figure 4D,E) activation, and the effect was better than TM. The expression levels of key proteins in the cGAS‐STING pathway (cGAS, STING, P‐IRF3/IRF3, and P‐P65/P65) in brain tissues of different groups were further detected by Western blot (WB). TPM could significantly reduce the high expression of cGAS and STING, and inhibit the phosphorylation of IRF3 and P65 (Figure 4F and Figure S36). Next, enzyme‐linked immunosorbent assay (ELISA) was used to measure the levels of various inflammatory factors in the homogenates of affected brain tissues. The pro‐inflammatory factors interleukin (IL‐1β and IL‐6) and tumor necrosis factor‐α (TNF‐α) were all significantly increased after ISIR injury, which were 2.89, 5.90, and 4.45 times higher than those in the Sham group, respectively, suggesting that ISIR induces an inflammatory storm in ischemic brain tissue. Under TPM and TM intervention, the levels of the three pro‐inflammatory factors could be significantly reduced, especially in the TPM group, where the levels of the abovementioned three pro‐inflammatory factors all dropped to normal levels (Figure 4G–I). IL‐10 and IL‐4, which also have anti‐inflammatory effects, were increased with the intervention of TPM (Figure 4J,K). The NAC group was close to the ISIR group because NAC has poor targeting of cerebral infarction tissue.

To further explore the mechanism by which TPM reverses neuroinflammation during ISIR, we co‐cultured HT22 cells and microglia (BV2) to simulate the microenvironment of brain tissue ISIR injury (Figure 4b). The distribution of mtDNA in BV2 cells in each group was observed by co‐staining with DAPI/dsDNA/TOM20. As shown in Figure 4c, compared with the Normoxia group, more abnormally distributed mtDNA were observed in the cytoplasm of BV2 cells in the H/R group, and TPM could significantly reduce the distribution of mtDNA in the cytoplasm in a dose‐dependent manner. We collected BV2 cells from different treatment groups for WB to detect the expression of cGAS‐STING signaling‐related proteins. As shown in Figure 4l–o and Figure S37, similar to the results in vivo, TPM could significantly inhibit the activation of the cGAS‐STING pathway. Moreover, TPM could significantly reduce the abnormal release of pro‐inflammatory factors (IL‐1β, IL‐6, and TNF‐α) in BV2 cells stimulated by H/R, and increase the secretion of anti‐inflammatory factors (IL‐4, IL‐10) (Figure S38). The above results fully demonstrate that TPM can inhibit the activation of the STING pathway and reverse neuroinflammation during ISIR.

### 2.5 TPM Improves Neuronal ER Stress

**FIGURE 5:**
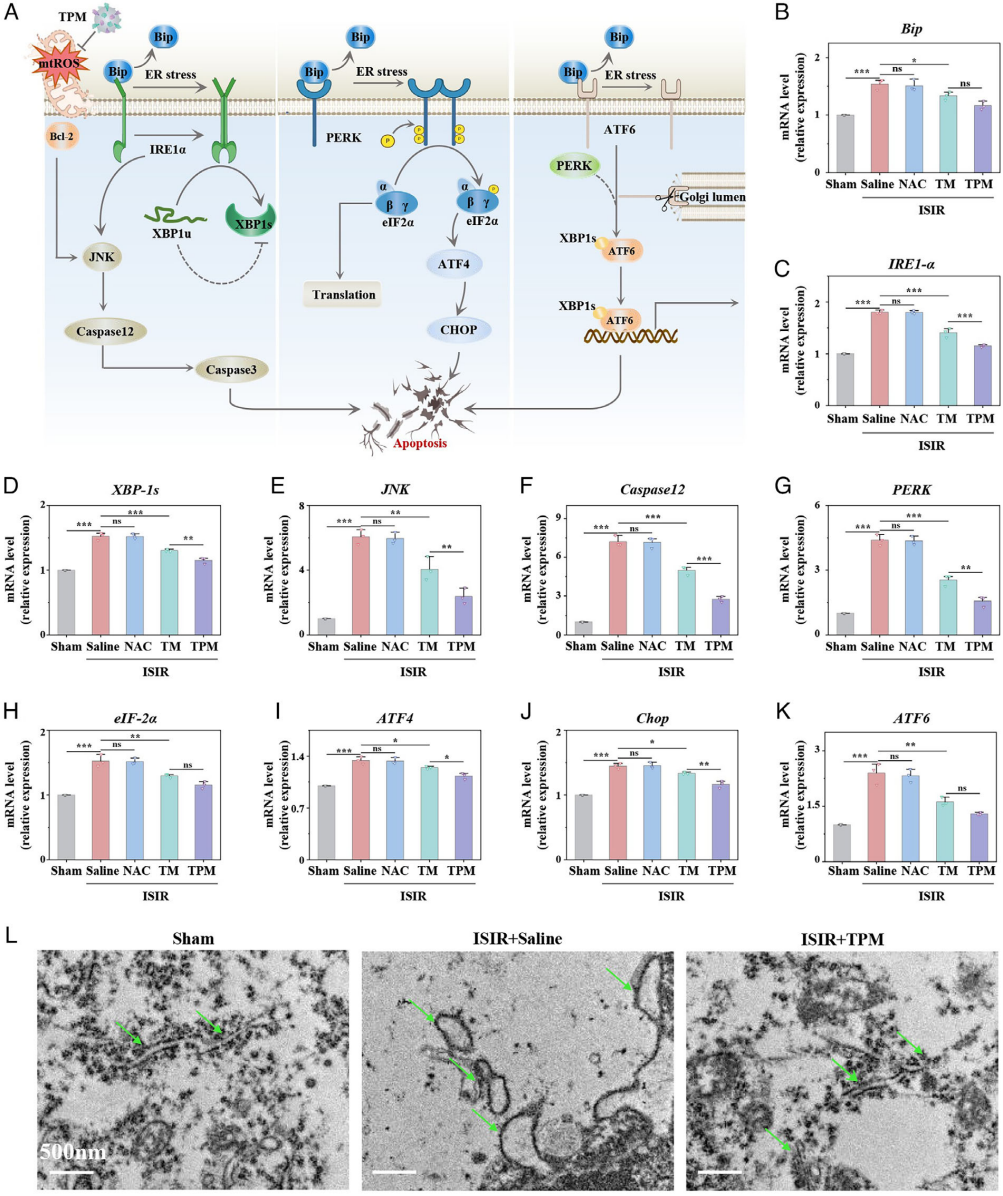
TPM improves ER stress. (A) Schematic diagram of ER stress and TPM protective effect under ISIR model. (B–K) Real‐time fluorescence PCR detection of expression levels of ER stress‐related factors (*Bip, IRE1‐α, XBP‐1s, JNK, Caspase12, PERK, eIF‐2α, ATF4, Chop, ATF6*) in brain tissue after different treatments. (L) TEM image of ER (indicated by the green) in brain tissue neurons from the Sham group, the ISIR group, and the TPM treatment group. Scale bar: 500 nm. Data were expressed as mean ± SE. (*n* = 3, ns: *p* > 0.05, ^**^p < 0.01, ^***^p < 0.001).

The ER membrane and the mitochondrial outer membrane contact each other to form the mitochondria‐associated ER membrane (MAM), which not only structurally connects the mitochondria and the ER tightly together, but also undertakes the material transport and signal molecule transmission between the two organelles [26]. Therefore, the mtROS burst from mitochondria can cause ER stress to hinder protein synthesis and release. Many unfolded and misfolded proteins accumulate in the ER lumen and bind to Bip to release three ER stress functional transmembrane molecules: inositol‐requiring enzyme 1 (IRE1) [27], PKR‐like endoplasmic reticulum kinase (PERK), and activating transcription factor 6 (ATF6) to activate neuronal apoptosis (Figure 5A). Therefore, these three proteins have been identified as markers of ER stress. We hypothesized that TPM could effectively eliminate mtROS to relieve ER stress, which would subsequently significantly reduce the mRNA levels of these three related pathways.

IRE1‐α causes the mRNA of XBP1, which can eliminate ER stress, to be cleaved into XBP1s before translation. In addition, IRE1α can also promote neuronal apoptosis by catalyzing the signaling pathway mediated by c‐Jun N‐terminal kinases (JNK) and Caspase12. PERK leads to the autophosphorylation of eIF‐2α, which causes the translation of the transcription factor ATF4, and then ATF4 induces the expression of Chop to initiate neuronal apoptosis [28]. We first explored the mRNA transcription levels of IRE1‐α and its downstream molecules in the brain tissues of each group through q‐PCR. The mRNA transcript levels of *Bip*, *IRE1‐α*, *JNK*, *Caspase12*, and *XBP‐1s* were significantly increased in the ISIR group. As expected, the abnormally elevated mRNA expression could be reversed to normal levels by TPM treatment (Figure 5B–F). Second, PERK leads to the autophosphorylation of eIF‐2α, which induces the transcription factor ATF4, and subsequently induces the expression of Chop to initiate neuronal apoptosis [29]. As shown in Figure 5G–J, the *PERK* mRNA transcript level in the ISIR group was 4.4 times that of the Sham group. After TPM treatment, the mRNA transcript levels of *PERK*, *eIF‐2α*, *ATF‐4*, and *Chop* decreased significantly. Third, unlike IRE1‐α and PERK, ATF6 is a type III transmembrane transcription factor that resides in the ER. When unfolded proteins accumulate in the ER, ATF6 dissociates from Bip and transfers to the Golgi apparatus, where it is cleaved by site 1 protease (S1P) and site 2 protease (S2P), releasing a fragment of the transcription factor to regulate neuronal apoptosis [30]. As shown in Figure 5K, the mRNA transcript level of *ATF6* in the ISIR group increased, 2.4 times that of the Sham group, while the TPM treatment group significantly reduced the transcript level of *ATF6*, and the effect was better than that of the TM group. In vitro, TPM was also shown to significantly reverse the increase in mRNA levels of IRE1‐α, PERK, and ATF6‐related pathway proteins in HT22 cells induced by H/R (Figure S39). The morphological structure of the ER is an important feature in maintaining its function of the ER. Finally, through TEM of neurons in brain tissue, the ER in neurons of the ISIR group was swollen and ribosomes were detached, while the ER structure almost returned to normal in the TPM group (Figure 5L). The above evidences show that TPM can effectively improve ER stress and maintain the normal morphological structure of the ER.

### 2.6 TPM Reduces Neurons Apoptosis

**FIGURE 6:**
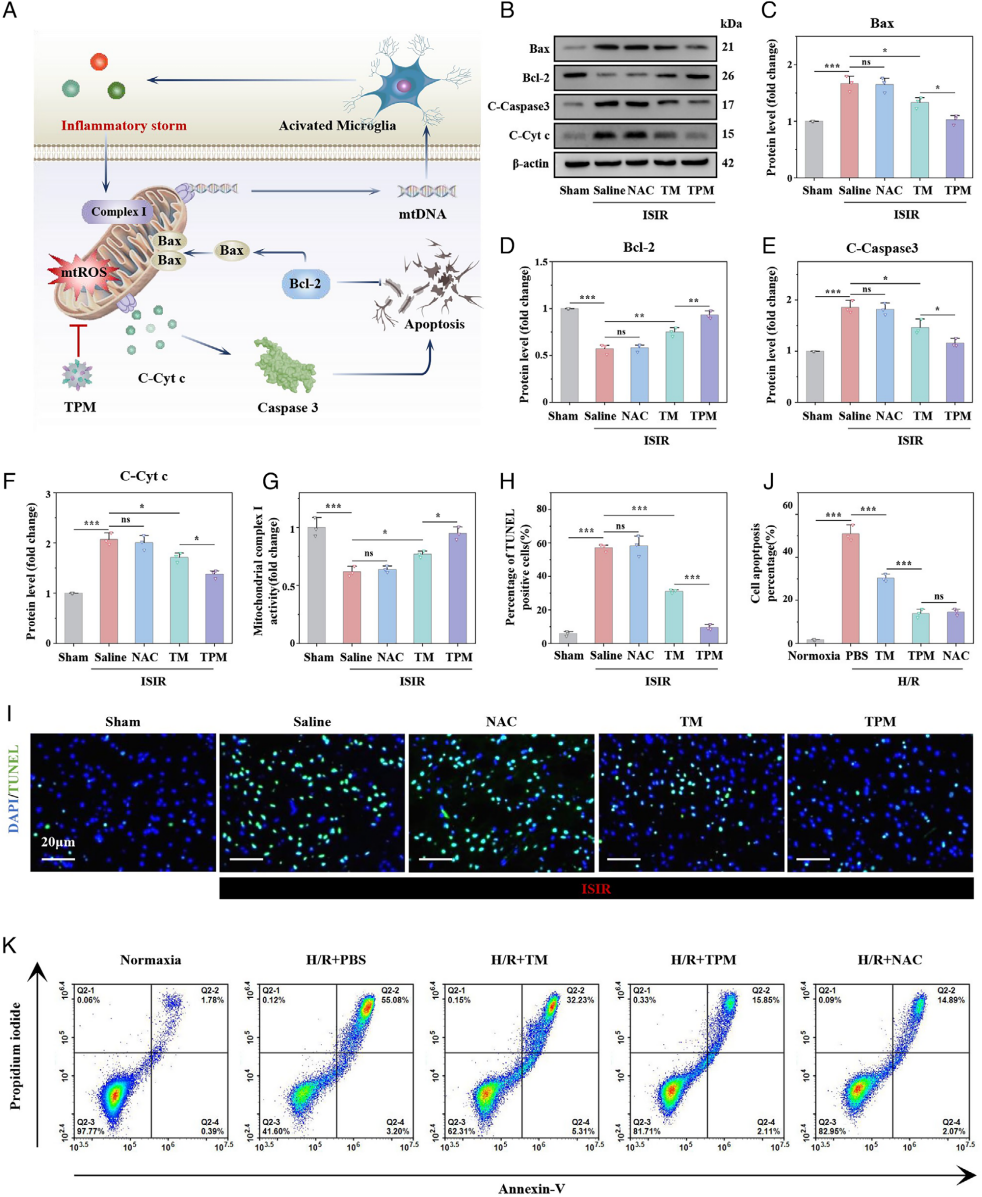
TPM reduces ISIR‐induced apoptosis. (A) Schematic diagram of cell apoptosis induced by endogenous and exogenous factors. (B–F) Western blot of apoptosis‐related proteins in cerebral infarct area, including representative images (B) and analysis of Bax (C), Bcl‐2 (D), C‐Caspase 3 (E), and C‐Cyt c (F). (G) Mitochondrial complex I activity. (H,I) TUNEL staining (H) and quantitative analysis (I) of brain tissue sections in each treatment group. Scale bar: 20 µm. (J,K) Annexin V‐FITC/PI flow cytometry results of HT22 cells under different treatment conditions (K) and quantitative statistical results of apoptotic cell rate (J). Data were expressed as mean ± SE. (*n* = 3, ns: *p* > 0.05, ^*^p < 0.05, ^**^p < 0.01, ^***^p < 0.001).

In ISIR, neuronal death is mainly mediated by intrinsic and extrinsic pathways (Figure 6A). In the intrinsic pathway, mitochondrial damage leads to cytosolic cytochrome c (C‐Cyt c)‐induced neuronal apoptosis [31]. In the exogenous pathway, inflammatory factors such as TNF‐α and IL‐6 inhibit the activity of mitochondrial complex I, aggravating neuronal mitochondrial damage to promote neuronal cell apoptosis [32]. We hypothesized that TPM could inhibit neuronal apoptosis caused by both pathways because TPM has a high degree of mitochondrial targeting and mitochondrial protective effects. To this end, we detected the expression levels of a series of apoptosis‐related proteins (Bax, Bcl‐2, C‐Caspase3, and C‐Cyt c) in brain tissues of different groups by WB. After ISIR injury, the expression levels of Bax, C‐Caspase3 and C‐Cyt c were significantly increased, while the expression levels of Bcl‐2 were significantly down‐regulated. After TPM intervention, the abnormal expressions of these proteins were corrected (Figure 6B–F). At the same time, TPM can also improve the decreased complex I activity caused by ISIR (Figure 6G). Subsequently, we analyzed cell apoptosis in brain tissue in each group through terminal deoxynucleotidyl transferase dUTP nick end labeling (TUNEL) staining. As shown in Figure 6H–I, the cell apoptosis rates in the ISIR group and NAC group were as high as 57.17% and 56.23%, respectively. The apoptosis rate in the TM group was reduced to 31.20%, and the apoptosis rate in the TPM group was significantly reduced to 9.68%. In vitro, flow cytometry results showed that TM could reduce the H/R‐induced cell apoptosis rate from 50.97% to 30.37%, while TPM could reduce the apoptosis rate to 13.97%, NAC at the dose of 100 µg mL^−1^ can also effectively improve the apoptosis of HT22 cells, which fully confirms the key role of ROS in the induction of neuronal ROS and the necessity of efficient elimination of ROS (Figure 6J,K). Through WB, the protein levels of Bax, C‐Caspase3, and C‐Cyt c in the H/R group were significantly higher than those in the normal group, and the levels of Bcl‐2 were lower than those in the normal group, indicating the apoptotic pathways were activated in HT22 cells. TPM could significantly reduce the activation of the above pathways because TPM strongly protects mitochondria (Figure S40). The above evidence fully proves that TPM can significantly alleviate neuronal apoptosis caused by exogenous and endogenous pathways in ISIR.

### 2.7 Transcriptomic Analysis of Brain Tissue

**FIGURE 7:**
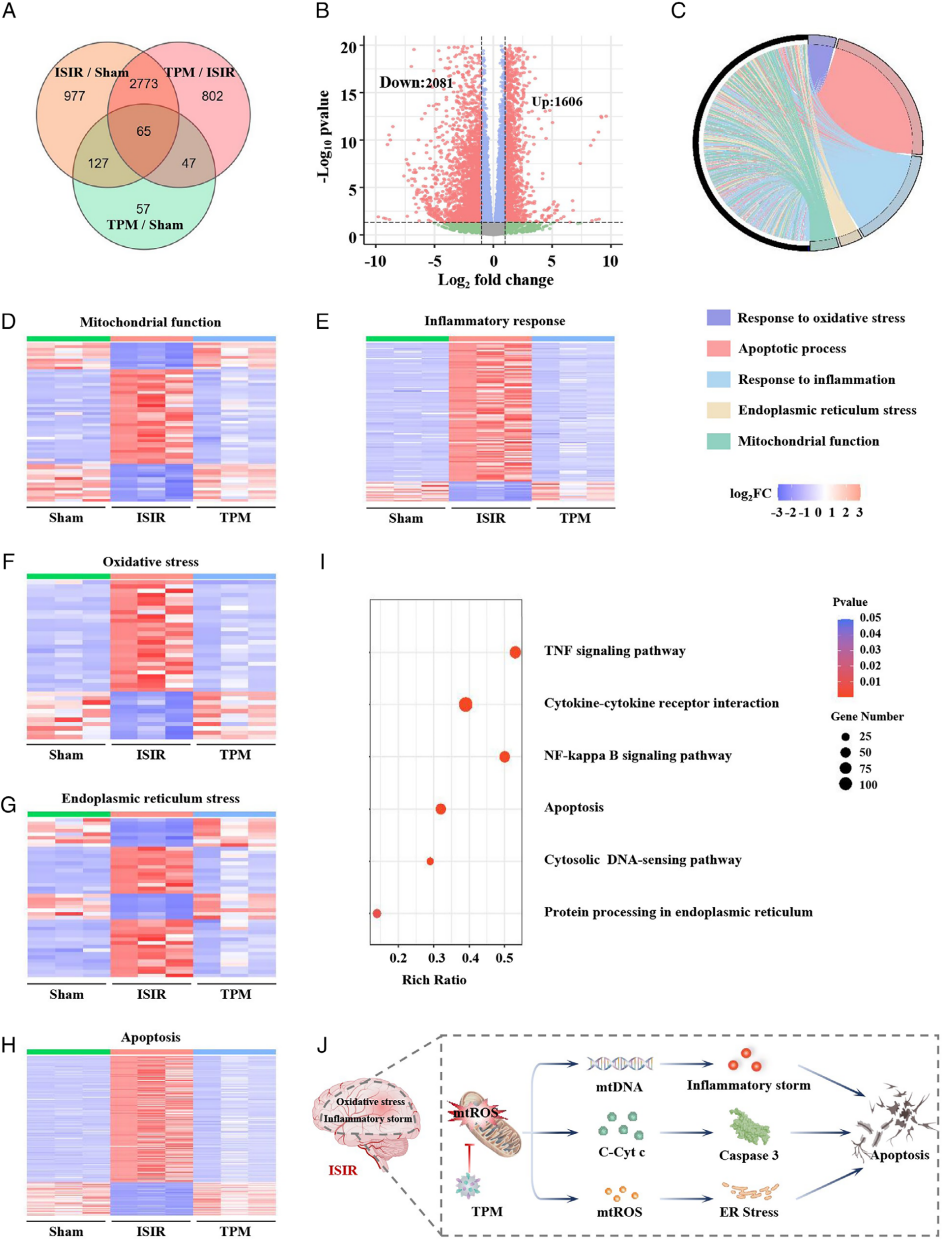
Transcriptomic analysis of brain tissue. (A) VENN/UpSetR graph analysis shows the differentially expressed genes (DEGs) between different comparison groups. (B) Volcano plot depicting DEGs determined between TPM‐treated and ISIR brain tissue, highlighting 1606 upregulated genes and 2081 downregulated genes (*P*‐value ≦ 0.05 and fold difference |log_2_FC| ≧ 1). (C) GO enrichment analysis of biological processes specifically involved in DEGs between the ISIR group and TPM‐treated group. (D–H) Heat map visualization of the DEGs between the Sham group, ISIR group, and TPM treated group within pathways as identified from the GO enrichment analysis, including mitochondrial function (D), inflammatory response (E), oxidative stress (F), ER stress (G), and apoptosis (H). (I) KEGG pathways enrichment analysis of DEGs in the TPM‐treated group versus the ISIR group. (J) Schematic diagram of TPM treating ISIR in rats.

Transcriptome sequencing (RNA‐Seq) analysis of brain tissues of rats in the Sham group, ISIR group, and TPM group was performed to verify the above‐mentioned TPM treatment mechanism. First, we used the VENN/UpSetR graph to analyze the expression of differentially expressed genes (DEGs) between groups. As shown in Figure 7A, there are 3687 DEGs between the TPM group and the ISIR group, and there are only 296 DEGs between the TPM group and the Sham group. Subsequently, |log_2_ *FC*| ≧ 1, *P* value ≦ 0.05 were used as comparison thresholds to draw a volcano plot. Compared with the ISIR group, 1606 genes were upregulated and 2081 genes were downregulated in the TPM group (Figure 7B). We further used the gene ontology database (GO) to conduct enrichment analysis on the above DEGs. As shown in Figure 7C, the DEGs in the ISIR group and the TPM group mainly involved five aspects: inflammatory response, response, and regulation of oxidative stress, cell apoptosis process, mitochondrial function, and ER stress. We performed gene expression cluster analysis on the above five biological processes. The gene expression patterns of the ISIR group and the other two groups were quite different, while the expression patterns of the TPM and Sham groups were similar, which fully confirmed that TPM may achieve relevant therapeutic effects by regulating the genes related to the above five biological processes (Figure 7D–H). In addition, we analyzed the relevant signaling pathways with the Kyoto Encyclopedia of Genes and Genomes database (KEGG). As shown in Figure 7I, compared with the ISIR group, the DEGs in the TPM group were enriched in inflammation‐related signaling pathways, including the cytoplasmic DNA sensing pathway, cytokine‐cytokine receptor interaction, TNF signaling pathway, and NF‐κB signaling pathway, suggesting that mitochondrial damage‐induced neuronal apoptosis and the cytoplasmic DNA sensing pathway‐induced inflammatory signaling pathways are the key to TPM treatment of ISIR. In addition, KEGG analysis demonstrated that the ER protein processing signaling pathway was associated with the treatment of TPM. In summary, the transcriptome results are consistent with the above experimental evidence, which fully proves that TPM effectively protects mitochondria by eliminating mtROS, thereby reducing inflammatory storm, apoptosis, and ER stress (Figure 7J).

### 2.8 Biocompatibility of TPM

**FIGURE 8:**
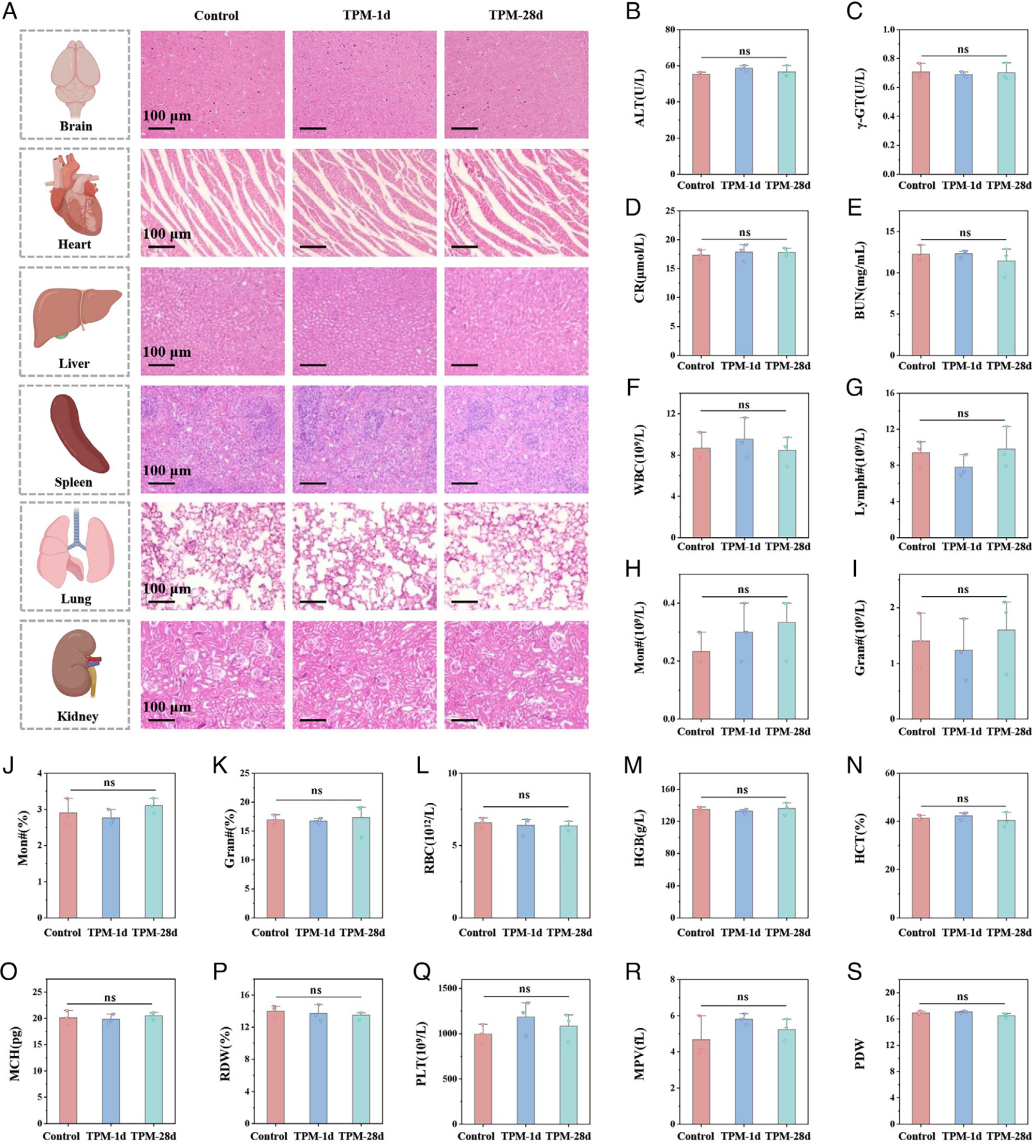
Biosafety of TPM. (A) HE staining of major organs 1 day and 28 days after TPM injection. (B–E) Liver and kidney function test results after TPM injection. (B) Alanine transaminase. (C) Blood urea nitrogen. (D) Creatinine clearance rate. (E) γ‐Glutamyl transferase. (F–S) Routine blood test results after TPM injection. (F) White blood cell count, (G) number of lymphocytes, (H) number of monocytes, (I) number of neutrophils, (J) monocyte percentage, (K) neutrophil percentage, (L) number of red blood cells, (M) number of hemoglobin, (N) hematocrit, (O) mean corpuscular hemoglobin content, (P) red blood cell distribution width coefficient of variation, (Q) platelet count, (R) mean platelet volume, (S) platelet distribution width. Data were expressed as mean ± SE. (*n* = 3, ns: *p* > 0.05).

The three key components of TPM: TA, PDA, and Mo‐based heteropolyacids, have been widely verified for their excellent biocompatibility. Finally, the biocompatibility and safety of TPM were evaluated. First, TPM could be effectively metabolized by the liver and kidneys after intravenous injection (Figure S20). TPM incubated HT22 cells and BV2 cells at a high concentration of 160 µg mL^−1^ (about 27 times the therapeutic concentration) for 24 h/48 h and did not cause changes in cell viability (Figures S41 and S42), indicating that TPM has excellent safety at the cellular level. We further verified the safety and biocompatibility of TPM at the animal level. Healthy SD rats and ISIR rats were given an extremely high dose of TPM (10 times the therapeutic dose) and sacrificed 1 and 28 days after injection to evaluate its acute and long‐term toxicity, respectively. Through HE staining, no obvious abnormalities were found in the heart, liver, spleen, lung, kidney, and brain (Figure 8A), indicating that TPM did not cause damage to major organs in the short term and long term. Next, we tested the blood biochemical indexes and blood routine indexes, and no abnormalities were found in the test results of various indicators, indicating that TPM would not affect the liver and kidney function (Figure 8B–E) and blood routine indexes (Figure 8F–S) of rats. The above results indicate that TPM exhibits good biocompatibility and safety both in vivo and in vitro.

## 3 Conclusion

In this work, we developed a sequentially targeted ternary composite nanomedicine‐TPM to effectively alleviate the neuronal cascade injury caused by ISIR. We first demonstrated that TA had a strong binding affinity to collagen III on the matrix exposed by the damaged BBB and mitochondrial outer membrane protein TOM20, while having negligible binding to glycosylation covering healthy blood vessels. Subsequently, we confirmed that TPM could specifically enter from the damaged BBB into neuronal mitochondria sequentially because TPM inherits the strong affinity of TA for collagen III and TOM20. In addition, PDA significantly enhanced the antioxidant capacity of TPM. Correspondingly, TPM specifically eliminated mtROS in neuronal mitochondria to mitigate mitochondrial damage and relieve ER stress. Moreover, TPM could reduce the release of mitochondrial mtDNA to inhibit the activation of the STING signaling pathway in microglia and reverse neuroinflammation. Thanks to the high specificity of sequential targeting, TPM had a high efficacy in treating ISIR. At an ultra‐low dose (3 mg kg^−1^), TPM almost eliminated the area of cerebral infarction (the area of cerebral infarction accounted for only 3.14%). Our innovation solves the challenges of specific targeting of the damaged BBB and mitochondria to achieve efficient treatment of ISIR.

The BBB is a complex, dynamic interface that precisely regulates the homeostasis of the central nervous system [33]. However, the BBB impedes the treatment of many central nervous system diseases because it blocks more than 98% of small‐molecule drugs and all macromolecules from entering the brain [34]. In recent years, many strategies have been developed to overcome the difficulties of drug delivery in the brain caused by the BBB, for example, by opening tight junctions, inhibiting the efflux system, regulating the lipid solubility of drugs, and simulating endogenous molecules to improve the ability of drugs to cross the BBB [35]. However, nonspecific interference with the normal physiological barrier function of the BBB may not only damage the endothelial cells and tissues of the brain but also allow harmful substances to enter the brain. Second, mimicking endogenous substances with drugs may interfere with the transport of neurotrophic substances and affect the homeostasis of the central nervous system, and changing the physicochemical properties of drugs may lead to changes in their pharmacological properties. In addition, some invasive drug delivery methods (intracerebroventricular injection, intracerebral or intraparenchymal injection, intrathecal injection, etc.) or non‐invasive drug delivery methods (inner ear administration, intranasal administration, etc.) that can bypass the BBB have been developed [35]. Unfortunately, these methods still have many disadvantages. For example, invasive administration may cause infection and severe discomfort to patients, while intranasal administration has problems such as limited dosage, elimination of mucosal fibers, and nasal mucosal permeability [36]. More importantly, these methods of promoting drug entry into the brain are non‐specific and cannot deliver the drugs to the pathological site to exert their effects. In addition, during the occurrence of ISIR, the blood flow in the lesion site is lower than that in the normal area, which will cause the drug to accumulate more in non‐lesion sites, which not only reduces the effectiveness of the drug, but also increases the drug's toxic side effects [37].

Driven by the rapid development of materials science and nanotechnology, a variety of nanomedicines for IS have been developed. These nanomedicines generally can passively target damaged areas because of their small size [38]. At the same time, the surface of nanomedicines is rationally modified to improve their surface charge, biocompatibility, etc., making them more stable in biological fluids and prolonging their circulation in the blood [39]. Although a variety of nanomedicines have been developed for ISIR, their therapeutic effects in ISIR are still limited, mainly due to their incompatibility with the damaged BBB and mitochondrial targeting. In our work, TPM can pass smoothly through the damaged BBB thanks to the modification of TA. Since neurons are very close to brain capillaries (rarely more than 8–20 µm) [35], TPM can be preferentially taken up by neurons. At the same time, the high binding force between TA and TOM20 gives TPM a strong ability to target mitochondria, allowing TPM to efficiently remove mtROS in situ in neuronal mitochondria.

The occurrence and development of ISIR is a complex pathological process, which mainly includes BBB destruction, oxidative stress, neuroinflammation, calcium overload, and the release of neurotoxins, and involves various types of cells (neurons, microglia, endothelial cells, and astrocytes, etc.) [40]. This study currently focuses on the protection of neuronal cells and the inhibition of microglial neuroinflammation, while endothelial cell damage and astrocyte activation have also been shown to be involved in the pathogenesis of ISIR [41]. Future work aims to make further improvements on the basis of TPM, so as to obtain a nanoplatform that can fully protect the neurovascular unit to completely alleviate ISIR‐mediated damage, such as oxidative stress, neuroinflammation, and cell apoptosis in the brain. Based on the findings of this study, modification of TA not only enables TPM to pass through the injured BBB barrier more efficiently, but also gives nanomedicine the capability to target to mitochondria. In summary, the TPM provides a promising strategy for the treatment of ISIR.

## 4 Materials and Methods

### 4.1 Materials

Details of reagents used in this study are provided in Table 1.

**TABLE 1:** List of reagents.

Reagents	Source	Catalogue
Gallic acid	Macklin	G823163
Phosphomolybdic acid	Macklin	P815551
Sodium carbonate anhydrous	Macklin	S818014
Dopamine hydrochloride	Macklin	D806618
Fluorescein isothiocyanate	Macklin	F6120
Methionine	Macklin	L812760
Riboflavin	Macklin	R817215
Nitro‐blue tetrazolium	Macklin	N814596
Terephthalic acid	Macklin	D835927
Hydrogen peroxide	Sigma	216763
Ferrous sulfate	Aladdin	F116338
Triphenyl tetrazolium chloride	Sigma	T8877
Reactive oxygen species assay kit	Solarbio	CA1410
Mitochondrial membrane potential assay kit with JC‐1	Beyotime	C2006
MitoSOX red mitochondrial superoxide indicator	Yeasen	40778ES50
Cell counting kit‐8	Beyotime	C0038
Malondialdehyde (MDA) test kit	Jiancheng Bioengineering Institute	A003‐1
Protease and phosphatase inhibitors	Beyotime	P1046
MitoTracker deep red FM	Yeasen	40743ES50
TUNEL apoptosis detection kit (FITC)	Yeasen	40306ES50
Enhanced BCA protein assay kit	Beyotime	P0010
Hoechst 33342	Beyotime	C1022
Antifade mounting medium with DAPI	Beyotime	P0131
Enhanced ATP assay kit	Beyotime	S0027
CheKine micro mitochondrial complex I activity assay kit	Abbkine	KTB1850
Annexin V‐FITC/PI apoptosis detection kit	Yeasen	40302ES50

### 4.2 Synthesis of TPM

Phosphomolybdic acid (0.18 g) was dissolved in 10 mL ddH_2_O, recorded as solution 1; tannic acid (0.2 g) was dissolved in 15 mL ddH_2_O, recorded as solution 2; sodium carbonate anhydrous (0.675 g) was dissolved in 10 mL ddH_2_O, recorded as solution 3; dopamine hydrochloride (0.1 g) was dissolved in 5 mL ddH_2_O, recorded as solution 4. Then the above solutions were poured into a round‐bottom flask in the order of 1, 2, 3, and 4 and stirred at 25°C for 12 h. The reaction solution was dialyzed for 2–3 days until it was colorless, and finally, TPM was obtained by freeze‐drying.

### 4.3 Synthesis of TPM‐FITC

TPM (40 mg) and NH_2_‐PEG‐NH_2_ (50 mg) were dissolved in 10 mL of Tris solution with a pH of 8.5, and reacted at 25°C for 12 h. The resulting solution was dialyzed for 6 h. The above drug (10 mg) was dissolved in 8 mL of ddH_2_O, and 2 mg of FITC was dissolved in 2 mL of DMSO. The two solutions were poured into a round‐bottom flask and stirred, and then reacted at 25°C for 6 h. The resulting solution was dialyzed to obtain TPM‐FITC.

### 4.4 Characterization

TEM images were taken with a TECNAI G2 high‐resolution transmission electron microscope; TPM was dispersed in ultrapure water, and its hydrodynamic diameter and Zeta potential were measured by a nanoparticle size Zeta potential analyzer. XPSPEAK software (version 4.1) was used to analyze the peaks of C1s, N1s, O1s, and Mo3d of TPM or TM. FT‐IR was obtained with a Bruker Vertex 70 spectrometer (2 cm^−1^). UV–visible spectra of TPM were obtained with a VARIAN CARY 50 UV/visible spectrophotometer. ICP‐MS was measured with a Thermo/Jarrell Ash Advantage Atomscan inductively coupled argon plasma spectrometer.

### 4.5 Detection of O_2_^·−^ Scavenging Capacity In Vitro

The NBT method was used to detect the O_2_^·−^ scavenging ability of TPM. In brief, different concentrations of TPM or TM (0, 0.5, 1, 2, 4, and 8 µg mL^−1^) were mixed with methionine (390 µL), riboflavin (6 µL), NBT (22.5 µL), and 1.5 mL of PBS (0.1 M, pH 7.4), and finally the volume was adjusted to 3 mL with ddH_2_O. Then, the mixed solution was irradiated under UV light for 5 min. Finally, the absorbance was detected at 560 nm to analyze the O_2_^·−^ scavenging ability of TPM or TM.

### 4.6 Detection of ·OH Scavenging Capacity In Vitro

The scavenging efficiency of TPM and TM on ·OH was determined by fluorescence spectrophotometry. In brief, different concentrations of TPM or TM (0, 0.125, 0.25, 0.5, 1, and 2 µg mL^−1^) were mixed with disodium terephthalate (300 µL), ferrous sulfate (300 µL), and H_2_O_2_ (30 µL), and finally fixed to 3 mL with PBS (0.01 M, pH 7.4). After standing for 6 min, the mixture was transferred to a cuvette, and the corresponding fluorescence intensity was scanned at an excitation wavelength of 320 nm.

### 4.7 Detection of H_2_O_2_ Scavenging Capacity In Vitro

The H_2_O_2_ scavenging ability of TM and TPM was tested by using a H_2_O_2_ detection kit (Beyotime). H_2_O_2_ solution of appropriate concentration was prepared, and different concentrations of TM or TPM were added. After reacting at room temperature for 30 min, the absorbance was detected using an enzyme reader to calculate the hydrogen peroxide concentration.

### 4.8 Detection of ONOO^−^ Scavenging Capacity In Vitro

Pyrogallol red was used to detect the scavenging ability of ONOO^−^ 10 µL of pyrogallol red (5 mM), 9 µL of ONOO^−^ (1.73 mM), and different concentrations (0, 2, 4, and 8 µg mL^−1^) of TPM or TM were mixed, and the pyrogallol red solution and pyrogallol red containing ONOO^−^ were used as controls. After 15 min of reaction, the absorbance of pyrogallol red at 540 nm was measured by UV–visible spectrophotometry to calculate the ONOO^−^ scavenging ability.

### 4.9 The Protein‐Coated Plate Experiment

The six‐well plates were coated with albumin and glycocalyx, then incubated at 4°C for 12 h. Then gently washed with PBS for three times, added TPM‐FITC solution (20 µg mL^−1^), and incubated at 4°C for 12 h away from light. After careful washing with PBS for three times, the images were taken under a fluorescence microscope, and the fluorescence intensity was measured by Image‐J software.

### 4.10 Molecular Docking

The crystal structures of Glycalyx, Collagen II, Albumin, and TOM20 proteins were downloaded from the PDB database with PDB IDs of 4BWE, 4AE2, 1AO6, and 4APO, respectively. In addition, the two‐dimensional structure of TA (PUBCHEM CID:16129778) is downloaded from the PUBCHEM database and converted into a three‐dimensional structure using Chem3D. All non‐specific molecules, water molecules, etc., are removed from the protein structure using PyMol 2.4.0. The protein and TA structures were converted to PDBQT format using OpenBable2.4.1 software. Then, PYRX software is used for molecular docking. Finally, the docking results were visualized and analyzed using PyMOL 2.4.0.

### 4.11 Construction of MCAO Model

A model of cerebral ischemia/reperfusion injury in MCAO rats was established using SD male rats (8 weeks of age) weighing 260–280 g. SD rats were purchased from Hunan STA Experimental Animal Co., Ltd. (SYXK (XIANG) 2020‐0019) (Changsha, China). All animal procedures in this manuscript were performed according to protocols approved by the Ethics Committee of Xiangya Medical College, Central South University, Ethics approval number: XMSB‐2023‐0188. The MCAO model was established by the suture method. Briefly, SD rats were anesthetized with sodium pentobarbital, and the left common carotid artery (CCA), external carotid artery (ECA), and internal carotid artery (ICA) were isolated. The CCA and ECA were ligated separately with nylon sutures, and then the suture was inserted from the CCA into the ICA to block the blood supply of the middle cerebral artery. The suture was removed 2 h later for reperfusion.

### 4.12 TTC Staining

Twenty‐four hours after MCAO modeling, the brain was removed, frozen at −20°C for 1 h, sliced, and incubated with 1% TTC for 10 min in the dark. After staining, the slices were fixed with 4% paraformaldehyde (PFA) and photographed with a camera, and finally, Image J was used to quantify the infarct area.

### 4.13 HE Staining

Twenty‐four hours after MCAO modeling, the brain was removed after perfusion with physiological saline and 4% PFA, dehydrated, paraffin‐embedded, and sliced, and HE staining was performed using HE staining solutions to observe tissue pathological changes, and photos were taken under a microscope.

### 4.14 TUNEL Staining

TUNEL staining was used to evaluate cell apoptosis. After dewaxing the paraffin sections, staining was performed according to the TUNEL kit (YESSEN, 40306ES50) instructions, and then DAPI (Solarbio, C0060) was used to stain the cell nuclei. Finally, the cells were observed under a fluorescence microscope.

### 4.15 Nissl Staining

Nissl staining is used to reflect the damage to neurons. After the paraffin sections are dewaxed, they are stained with 1% toluidine blue dye and finally photographed under a microscope.

### 4.16 ROS Staining

After the paraffin sections were dewaxed, they were stained with a sufficient amount of DHE working solution, and the nuclei were stained with DAPI, and then photographed with a fluorescence microscope.

### 4.17 Immunofluorescence Staining

Dewax and hydrate the paraffin sections, perform antigen retrieval with EDTA, and permeabilize with PBS containing 0.1% tritonX‐100. Then use 5% BSA‐PBS solution to block. Use 1% BSA to dilute the primary antibody and incubate overnight at 4°C. Use 1% BSA to dilute the fluorescent secondary antibody Alexa Flour 488 (1:500) or Alexa Flour 555 (1:500) incubation in the dark. Finally, add DAPI solution to stain the nucleus. Observe under a fluorescence microscope. Antibody details for immunofluorescence are provided in Table 2.

**TABLE 2:** Antibody list 1.

Primary/Secondary antibody	Company	Cat. No	Dilution
Anti‐dsDNA antibody	Abcam	AB27156	1:500
TOM20 polyclonal antibody	Proteintech	11802‐1‐AP	1:500
CD206 recombinant antibody	Proteintech	81525‐1‐RR	1:100
iNOS antibody	Affinity	AF0199	1:100
Alexa Fluor 555	Invitrogen	A21428	1:500
Alexa Fluor 488	Invitrogen	A11029	1:500

### 4.18 Immunohistochemistry

The paraffin sections were dewaxed and hydrated, and antigen repair was performed with EDTA. An appropriate amount of endogenous catalase blocker was added for incubation. 5% BSA was used for blocking, and an appropriate amount of cGAS antibody and STING antibody was added for incubation at 4°C overnight. An appropriate amount of reaction enhancement solution was added for incubation, and an appropriate amount of enzyme‐labeled goat anti‐mouse and rabbit IgG polymer was added for incubation for 30 min. Diaminobenzidine solution was added for incubation, and the reaction was terminated by soaking in tap water. The sections were counterstained with hematoxylin and rinsed with tap water. The sections were differentiated with 1% hydrochloric acid and ethanol and soaked in tap water to turn blue. The sections were dehydrated and sealed with neutral resin, and observed and photographed under a histochemical microscope. Antibody details for immunohistochemistry are provided in Table 3.

**TABLE 3:** Antibody list 2.

Primary/Secondary antibody	Company	Cat. No	Dilution
cGAS	Proteintech	29958‐1‐AP	1:500
STING	Proteintech	19851‐1‐AP	1:500

### 4.19 MDA Content Detection

Take the above supernatant and detect the MDA content therein according to the instructions of the MDA kit (Jiancheng Bioengineering Institute, A003‐1).

### 4.20 Inflammatory Factor Detection

A 10% homogenate of brain tissue or BV2 cells was obtained according to the above method. The ELISA kit (Tables 4 or 5) was used to evaluate the content of IL‐1β, IL‐6, TNF‐α, IL‐4, and IL‐10 in brain tissue or BV2 cells.

**TABLE 4:** List of ELISA kits for rat.

Primary/Secondary antibody	Company	Cat. No
Rat IL‐10 (Interleukin 10) ELISA kit	Elabscience	E‐EL‐R0016
Rat IL‐4 (Interleukin 4) ELISA kit	Elabscience	E‐EL‐R0014
Rat TNF‐α (Tumor Necrosis Factor Alpha) ELISA kit	Elabscience	E‐EL‐R2856
Rat IL‐6 (Interleukin 6) ELISA kit	Elabscience	E‐EL‐R0015
Rat IL‐1β (Interleukin 1 Beta) ELISA kit	Elabscience	E‐EL‐R0012

**TABLE 5:** List of ELISA kits for BV2.

Primary/Secondary antibody	Company	Cat. No
Mouse IL‐10 (Interleukin 10) ELISA kit	Elabscience	E‐EL‐M0046
Mouse TNF‐α (Tumor Necrosis Factor Alpha) ELISA kit	Elabscience	E‐EL‐M3063
Mouse IL‐4 (Interleukin 4) ELISA kit	Elabscience	E‐EL‐M0043
Mouse IL‐6 (Interleukin 6) ELISA kit	Elabscience	E‐EL‐M0044
Mouse IL‐1β (Interleukin 1 Beta) ELISA kit	Elabscience	E‐EL‐M0037

### 4.21 Protein Extraction and WB

An appropriate amount of brain tissue was weighed into the homogenate tube, and RIPA buffer containing benzoyl fluoride was added to crack the sample, homogenized at 4°C for 5–10 min, and then the tissue homogenate was centrifuged at 13,000 rpm at 4°C for 15 min. The supernatant was carefully absorbed, and the protein concentration was detected with the BCA kit. The protein was denatured at 99°C for 10 min after the proper loading buffer was added. Samples were taken at 40 mg protein per well. By SDS‐PAGE electrophoresis, the protein in the sample is divided into bands in the gel according to molecular weight. Then, the protein divided into bands in the gel was transferred to the PVDF membrane by the wet transfer method, and then sealed with 5% skim milk for 1 h. The primary antibody was diluted according to the antibody dilution ratio in the table below, and the membrane and the primary antibody were incubated at 4°C for 16 h, then TBST was washed three times for 5 min each time, and the membrane and the diluted secondary antibody were incubated at room temperature for 1 h. The chemiluminescence imaging system was used to realize visualization, and Image J software was used to quantify the band strength. Antibody details for WB are provided in Table 6.

**TABLE 6:** Antibody list 3.

Primary/Secondary antibody	Company	Cat. No	Dilution
β‐actin	Proteintech	66009‐1‐Ig	1:20000
P‐P65	Affinity	AF2006	1:1000
P65	Affinity	BF8005	1:1000
P‐IRF3	Affinity	AF2436	1:1000
IRF3	Affinity	DF6895	1:1000
C‐Cyt c	Proteintech	10993‐1‐AP	1:5000
C‐Caspase 3	Wanleibio	WL02117	1:500
Bax	Abcam	ab32503	1:5000
Bcl‐2	Affinity	BF9103	1:1000
Goat anti rabbit IgG H&L (HRP)	Abclonal	AS014	1:6000
Goat anti mice IgG H&L (HRP)	Abclonal	AS003	1:5000
cGAS	Proteintech	29958‐1‐AP	1:1000
STING	Proteintech	19851‐1‐AP	1:1000

### 4.22 Detection of Mitochondrial Complex I Activity

Different groups of brain tissue samples were prepared according to the CheKine micro mitochondrial complex I activity assay kit instructions, and the working liquid was prepared. A 96‐well plate was taken, 10 µL of samples, 200 µL of working liquid, and 15 µL of Working ReagentVI were added to each well, and after fully mixing, the initial light absorption value A1 of 0 min at 340 nm and the light absorption value A2 after 2 min were immediately ready for calculation.

### 4.23 RNA Isolation and Quantitative Real‐Time PCR

Total RNA from brain tissues or HT22 cells was isolated by Trizol. cDNA was synthesized using PrimeScript RT kit (Takara, Japan). qPCR reactions were performed using TB Green Premix Ex Taq (Takara, Japan). Results were calculated by the 2^−ΔΔ^ *^Ct^* method. Primer sequences for qPCR are provided in Table 7 and Table 8.

**TABLE 7:** List of qPCR primers for tissue.

Gene name	Primer sequence (5′‐3′)
Bip‐F	TAC TCG AAT TCC AAA GAT TCA G
Bip‐R	TCA AGC AGA ACC AGG TC
PERK‐F	TCC TGT CTT GGT TGG GTC TG
PERK‐R	TGC GTG CTC CGC TTA TTC
eIF2α‐F	TTA TGC CTG CGA AAG CAA C
eIF2α‐R	TTC CAT TTG TCC TCG AAG GT
ATF4‐F	GCC AAG CAC TTC AAA CCT CA
ATF4‐R	GCA TGG TTT CCA GGT CAT CC
Chop‐F	CCT GAA AGC AGA AAC CGG TC
Chop‐R	CCT CAT ACC AGG CTT CCA GC
IRE1α‐F	AGA GCC CAT CAC CTT GCT T
IRE1α‐R	TGA TCC TGC CAT GTG CGT T
Caspase12‐F	ATA GCC ACT GCT GAT ACA GA
Caspase12‐R	CCA CTC TTG CCT ACC TTC C
JNK‐F	AGT GTA GAG TGG ATG CAT GA
JNK‐R	ATG TGC TTC CTG TGG TTT AC
XBP1s‐F	CTG AGT CCG CAG CAG GTG
XBP1s‐R	GAC CTC TGG GAG TTC CTC CA
ATF6‐F	GGA TTT GAT GCC TTG GGA GTC AGA C
ATF6‐R	ATT TTT TTC TTT GGA GTC AGT CCA T
β‐actin‐F	CAC TGC CGC ATC CTC TTC CT
β‐actin‐R	AAC CGC TCA TTG CCG ATA GTG

**TABLE 8:** List of qPCR primers for BV2 cell.

Gene name	Primer sequence (5′‐3′)
Bip‐F	TGT GTG TGA GAC CAG AAC CG
Bip‐R	TAG GTG GTC CCC AAG TCG AT
PERK‐F	AGG CTT TAA CTT CCC GCA TT
PERK‐R	AGT GCC AGA CTG AAA GTA AAT ACG
eIF2α‐F	CCG CTC TTG ACA GTC CGA G
eIF2α‐R	GCA GTA GTC CCT TGT TAG TGA CA
ATF4‐F	CCT GAA CAG CGA AGT GTT GG
ATF4‐R	TGG AGA ACC CAT GAG GTT
Chop‐F	GTC CCT GCC TTT CAC CTT GG
Chop‐R	GGT TTT TGA TTC TTC CTC TTC G
IRE1α‐F	CCT ACA AGA GTA TGT GGA GC
IRE1α‐R	GGT CTC TGT GAA CAA TGT TGA GAG
Caspase12‐F	ATG GCG GCC AGG AGG ACA CAT G
Caspase12‐R	CTA ATT CCC GGG AAA AAG GTA G
JNK‐F	GGT ATG CCC AAG AGG ACA GAG GA
JNK‐R	AGC CCA GAT AGA GCC AGT CGT AA
XBP1s‐F	CTG AGT CCG AAT CAG GTG CAG
XBP1s‐R	GTC CAT GGG AAG ATG TTC TGG
ATF6‐F	TCG CCT TTT AGT CCG GTT CTT
ATF6‐R	GGC TCC ATA GGT CTG ACT C
β‐actin‐F	ACG AGG CCC AGA GCA AGA
β‐actin‐R	TTG GTT ACA ATG CCG TGT TCA

### 4.24 In Vivo Fluorescence Imaging

The experimental animals were subjected to MCAO modeling, and TPM‐FITC was injected sublingually. After 1 h, the heart, liver, spleen, lung, kidney, and brain tissues were taken and immersed in PBS. The fascia and blood vessels on the surface of the tissues were peeled off and imaged under a Leica fluorescence microscope.

### 4.25 Cell Culture

HT22 cells and BV2 cells were purchased from Wuhan Servicebio Technology Co., Ltd. High‐glucose DMEM medium with 10% fetal bovine serum and 1% penicillin‐streptomycin was used for culture, and incubated in an incubator at 37°C and 5% CO_2_.

### 4.26 Cell Hypoxia/Reoxygenation Model

BV2 cells were planted in the upper chamber of Transwell pore plate at a concentration of 1 × 10^5^, while HT22 cells were planted in the lower chamber of the Transwell pore plate at a concentration of 1 × 10^5^ to create a co‐culture system, which was randomly divided. After incubation for 12 h, the cell culture base of the PBS group, TM group, and TPM group was replaced with a culture base containing 450 µM CoCl_2_. In addition, the TM and TPM groups contained two drugs, respectively, and were cultured for 16 h. The culture base containing cobalt chloride was replaced with the normal culture base containing only the corresponding drugs and incubated for 6 h. After culture, HT22 cells in the lower chamber were collected for Western blot and qPCR, and BV2 cells in the upper chamber were collected for Western blot, and the culture medium was collected for detection of inflammatory factors.

### 4.27 CCK8 Assay

HT22 cells and BV2 cells were seeded in 96‐well plates at a density of 1 × 10^5^. After incubation for 24 h, the medium was replaced with a culture base containing (0, 0.625, 1.25, 2.5, 5, 10, 20, 40, 80, and 160 µg mL^−1^) TPM, respectively. The 96‐well plates were removed at 24 and 48 h, and the culture medium was replaced with a culture base containing 10% CCK8, and incubated at 37°C for 30 min. The light absorption value was measured at 450 nm using an enzyme marker.

### 4.28 Measurement of Intracellular MitoSOX Levels

To further measure intracellular MitoSOX levels, HT22 cells were seeded at 1 × 10^5^/well in 6‐well plates and cultured for 24 h. After incubation with TPM under CoCl_2_ conditions for 16 h, they were incubated with complete medium without CoCl_2_ and TPM for another 6 h, stained according to the instructions, and finally analyzed by flow cytometry (Cytek, DxpAthena, 01‐DXPSF13‐01) or fluorescence microscope.

### 4.29 Measurement of Intracellular ROS Levels

The cells were treated as described above, then stained according to the instructions of the ROS detection kit, and finally analyzed by flow cytometry (Cytek, DxpAthena, 01‐DXPSF13‐01) or fluorescence microscopy.

### 4.30 MMP Measurement

MMP was measured using the JC‐1 kit (Beyotime, C2005). Cells were treated as described above, stained according to the kit instructions, and analyzed by flow cytometry (Cytek, DxpAthena, 01‐DXPSF13‐01) and fluorescence microscopy.

### 4.31 Detection of ATP Generation

ATP generation was detected using an ATP detection kit (Beyotime, Shanghai, China). Briefly, cells were treated as described above, and the detection was performed according to the instructions of the kit.

### 4.32 Subcellular Localization

Colocalization of TPM with mitochondria was imaged by fluorescence microscopy. HT22 cells were seeded at 5 × 10^4^/well in 24‐well plates, cultured overnight, and then treated with TPM‐FITC for 12 h. They were stained with MitoTracker (Beyotime, Shanghai, China) for 30 min and Hoechst for 10 min at 37°C. Images were acquired using a fluorescence microscope (Zeiss, LSM900, Germany) and analyzed using ImageJ software (NIH, MD, USA).

### 4.33 Safety Assessment

The normal group was SD male rats without drug administration, while the drug administration group was given 10 times the therapeutic dose (30 mg kg^−1^) of TPM. The blood of the two groups of rats was taken for blood routine examination at 24 h and 28 days of drug administration, and the heart, liver, spleen, lung, kidney, and brain tissues were taken for HE staining.

### 4.34 Statistical Analysis

All experiments were performed in parallel at least three times, and the results were expressed as mean ± standard error. Analyze data using GraphPad Prism, Image J, and Origin. The statistical method was one‐way analysis of variance, and *p* < 0.05 was considered statistically significant.

## Conflicts of Interest

The authors declare no conflicts of interest.

## Supporting information




**Supporting File 1**: exp270068‐sup‐0001‐SuppMat.docx

## Data Availability

The main data supporting the results in this study are available within the paper and its Supporting Information. The other data that support the findings of this study are available from the corresponding author upon reasonable request.
