# Supplementary material for: Specifically Breaking Through the Injured Blood‐Brain Barrier With Tannic Acid‐Based Nanomedicine for Ischemic Stroke Ischemia Reperfusion Treatment
Source: Exploration (Beijing). 2025 Sep 9;5(5):20240388. doi: 10.1002/EXP.20240388 (PMC12561273; doi:10.1002/EXP.20240388)
Supplement: Supplementary file 1 — Supporting File 1: exp270068‐sup‐0001‐SuppMat.docx [file EXP2-5-20240388-s001.docx]

**Supporting Information**

**Specifically breaking through the injured blood-brain barrier with tannic acid-based nanomedicine for ischemic stroke ischemia reperfusion treatment**

Xiaojing Shi^1,2,3†^, Shuya Wang^1,2†^, Tingli Xiong^1,2^, Ruishi Li^1,2^, Wenxuan Zheng^1,2^, Wensheng Chen^1,3^, Tianjiao Zhao^1,2^, Yongqi Yang^1,2^, Xiaohong Ying^1,2^, Weimin Qi^1,2^, Yingci Xia^1,2^, Jue Wang^1,2^, Yuqi Zhang^6^, Yayun Nan^7*^, Qiong Huang^1,3*^, Kelong Ai^2,4,5,8*^

^1^Department of Pharmacy, Xiangya Hospital, Central South University, Changsha, 410008, China.

^2^Xiangya School of Pharmaceutical Sciences, Central South University, Changsha, 410013, China.

^3^National Clinical Research Center for Geriatric Disorders, Xiangya Hospital, Central South University, Changsha, 410008, China.

^4^Hunan Provincial Key Laboratory of Cardiovascular Research, Xiangya School of Pharmaceutical Sciences, Central South University, Changsha, 410013, China.

^5^Key Laboratory of Aging-related Bone and Joint Diseases Prevention and Treatment, Ministry of Education, Xiangya Hospital, Central South University, Changsha, 410008, China.

^6^Communication design, Royal Melbourne Institute of Technology University, 124 La Trobe St, Melbourne VIC 3000, Australia.

^7^Geriatric Medical Center, People’s Hospital of Ningxia Hui Autonomous Region, Yinchuan, Ningxia, 750002, China.

^8^FuRong Laboratory, Changsha 410078 Hunan. China.

^†^The authors Xiaojing Shi and Shuya Wang contributed equally to the work.

*Corresponding authors: qionghuang@csu.edu.cn (Prof. Qiong Huang), nanyy_2009@163.com (Prof. Yayun Nan), aikelong@csu.edu.cn (Prof. Kelong Ai)

**
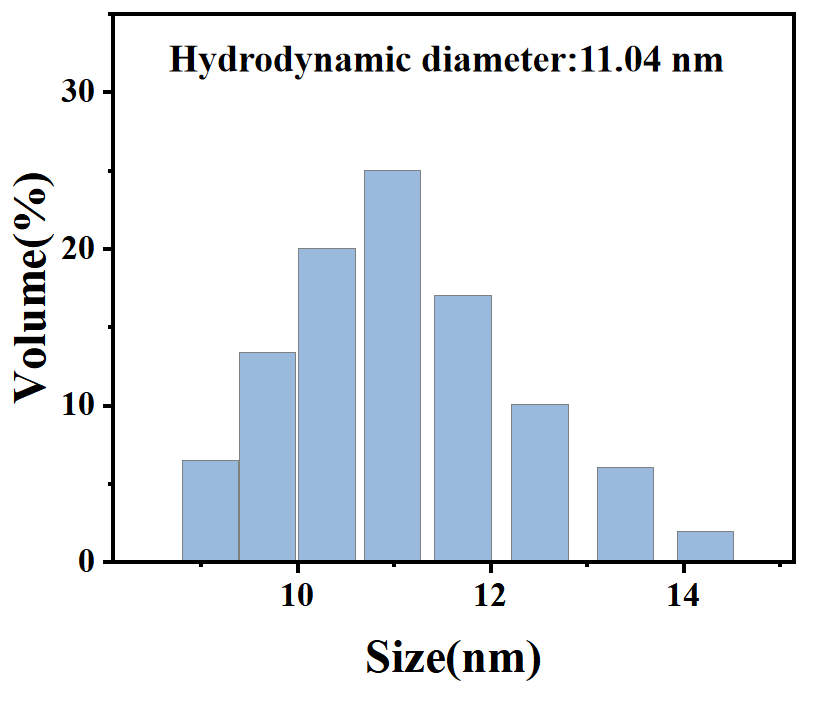
**

**Figure S1. Hydrodynamic diameter of TPM.**

**
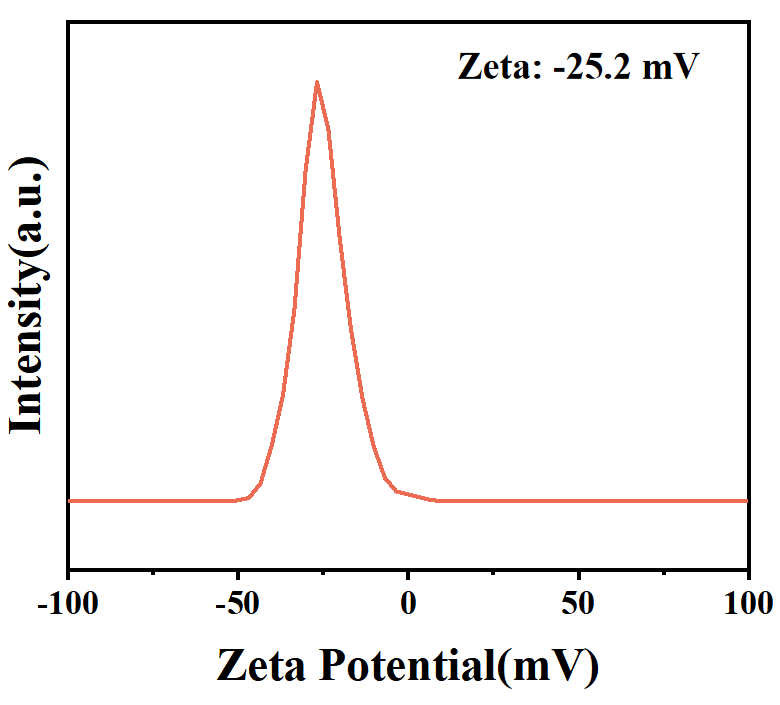
**

**Figure S2. Zeta potential determination of TPM.**

**
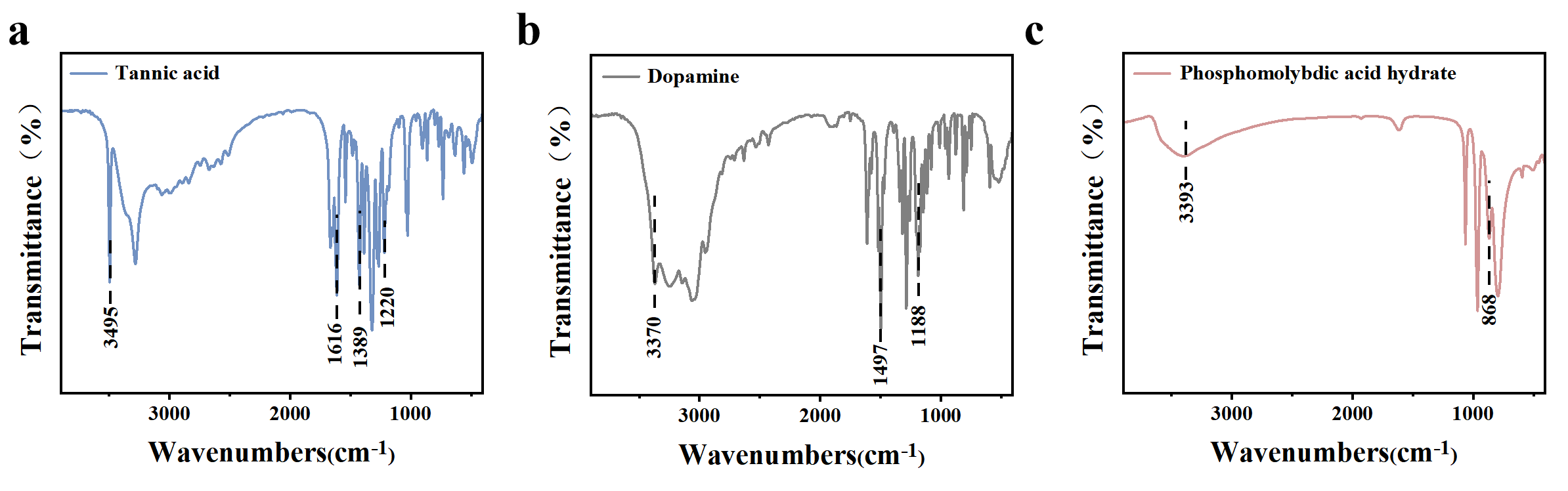
**

**Figure S3. FT-IR spectrum of** **Tannic acid, Dopamine and Phosphomolybdic acid hydrate. a**) FT-IR spectrum of Tannic acid, characteristic absorption peaks of hydroxyl, carbonyl, benzene ring and phenolic hydroxyl can be detected at 3495 cm^-1^, 1616 cm^-1^, 1389 cm^-1^ and 1220 cm^-1^. **b**) FT-IR spectrum of dopamine, Characteristic absorption peaks of hydroxyl, benzene ring and phenolic hydroxyl can be detected at 3370 cm^-1^, 1497 cm^-1^ and 1188 cm^-1^. **c**) FT-IR spectrum of Phosphomolybdic acid hydrate, characteristic absorption peaks of hydroxyl and molybdenum can be detected at 3393 cm^-1^, 868 cm^-1^.

**
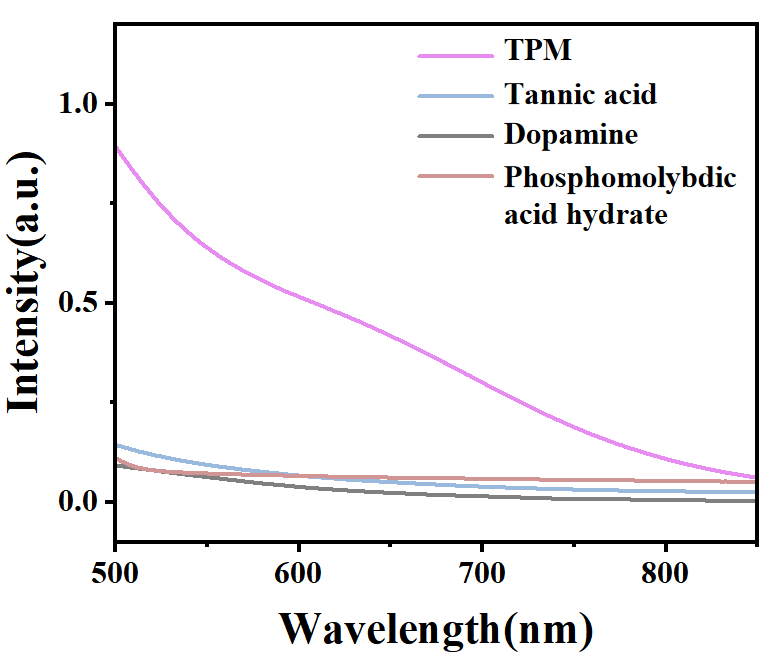
**

**Figure S4. Vis-NIR spectrum of TPM, Tannic acid, Dopamine and Phosphomolybdic acid hydrate.**

**
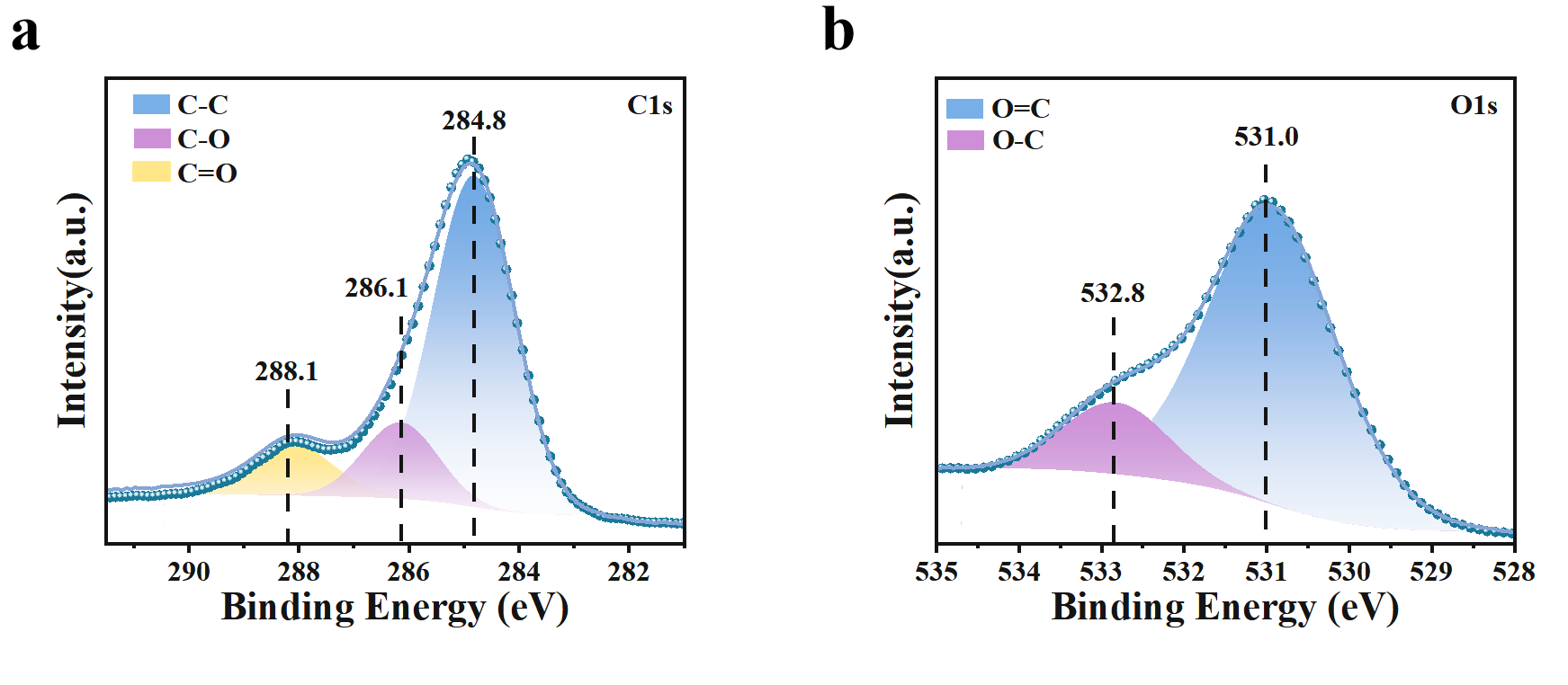
**

**Figure S5. XPS spectrum of TPM. a**) C1s narrow scan XPS spectrum. **b**) O1s narrow scan XPS spectrum.

**
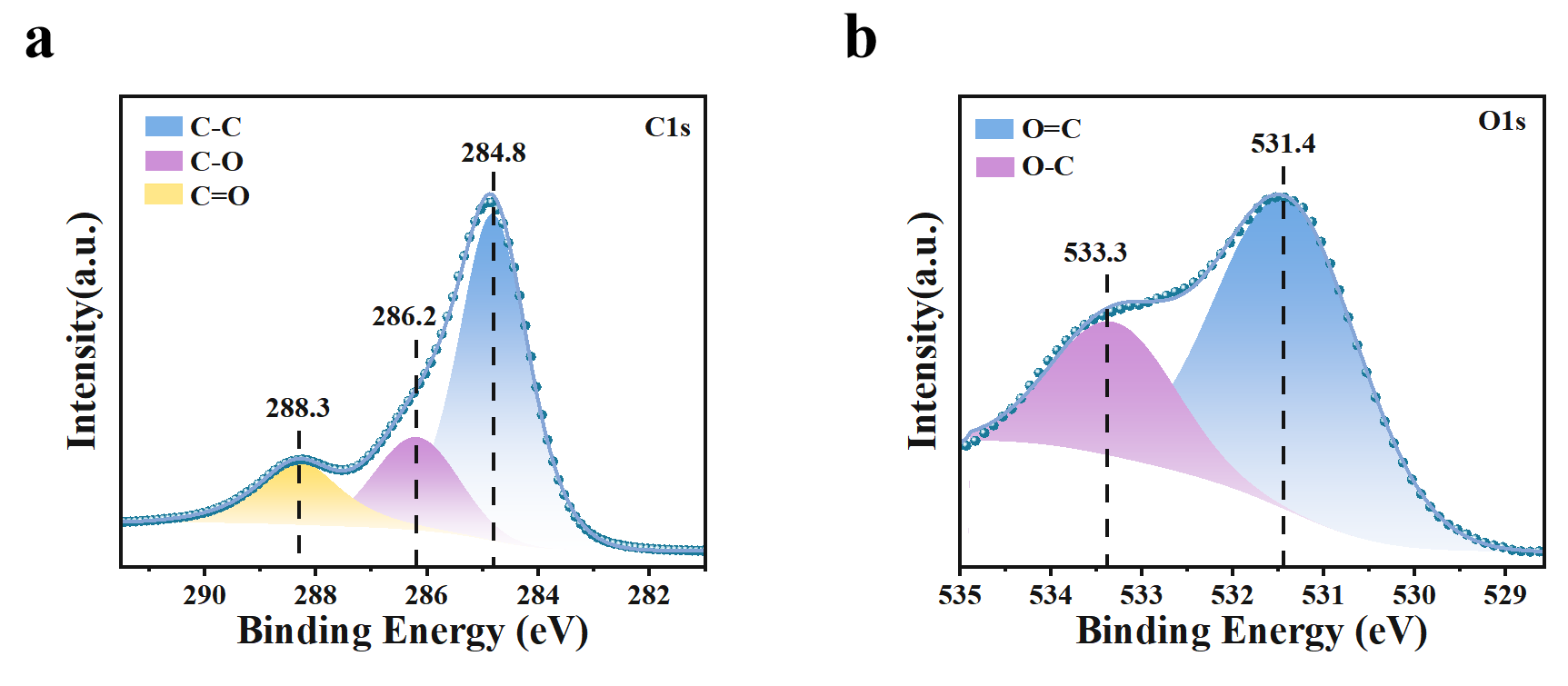
**

**Figure S6. XPS spectrum of TM. a**) C1s narrow scan XPS spectrum. **b**) O1s narrow scan XPS spectrum.


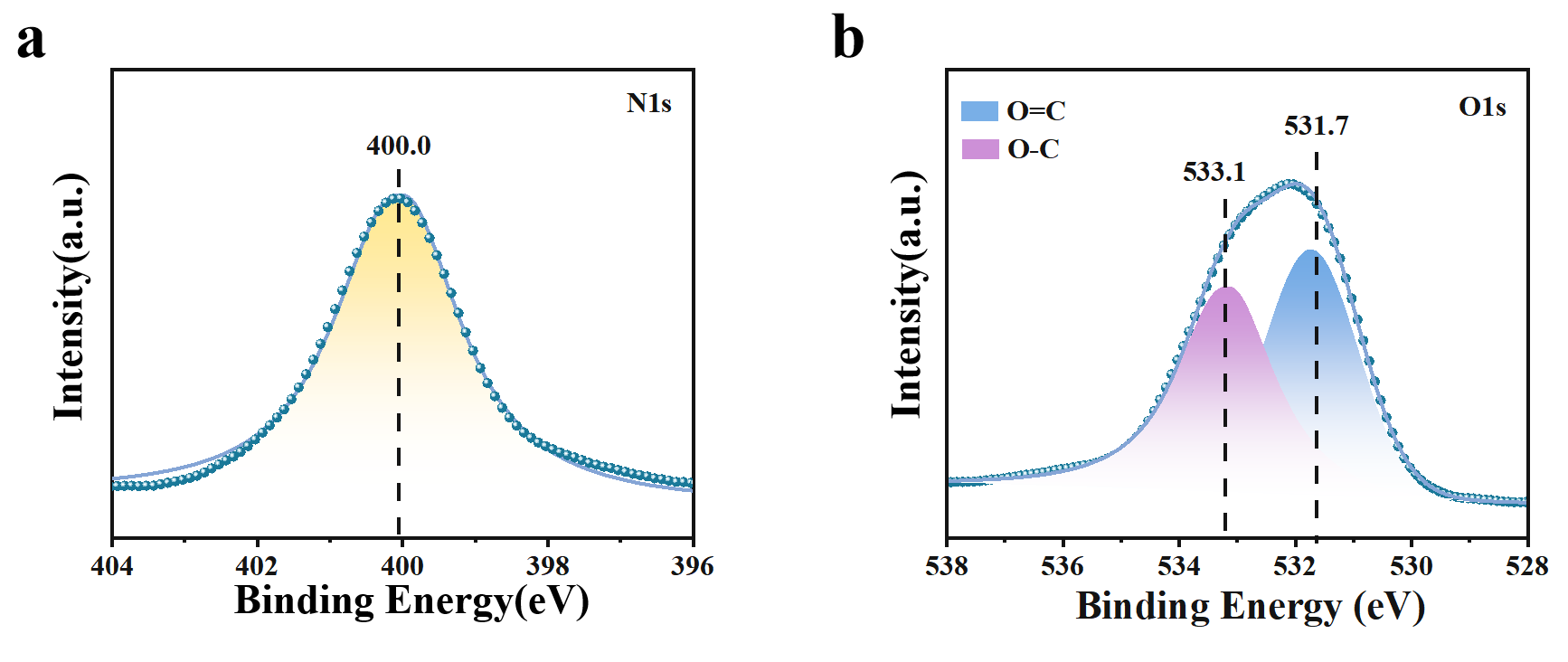


**Figure S7.** **XPS spectrum of TPM after reaction with H_2_O_2_. a**) N1s narrow scan XPS spectrum. **b**) O1s narrow scan XPS spectrum.


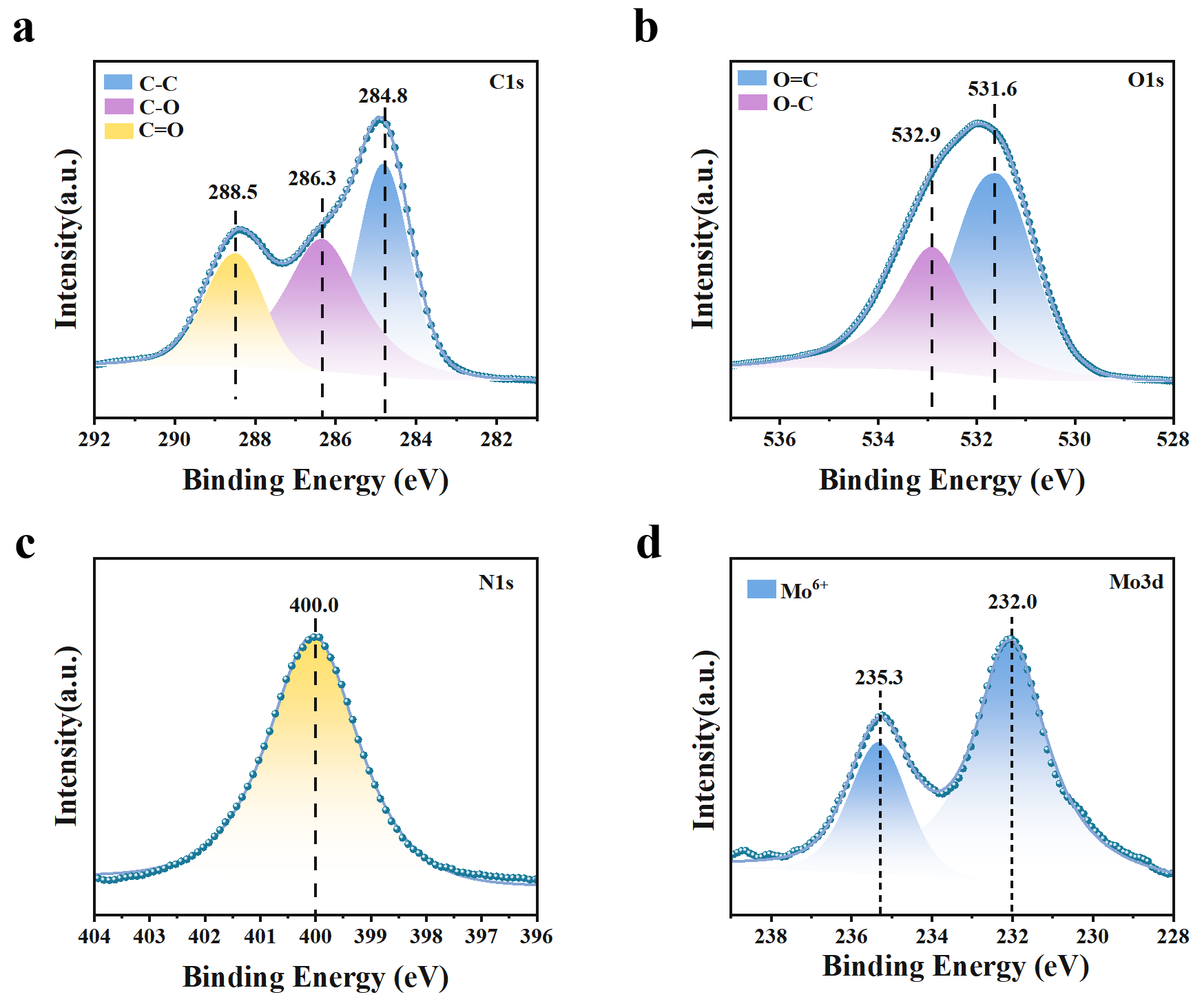


**Figure S8.** **XPS spectrum of TPM after reaction with·OH. a**) C1s narrow scan XPS spectrum. **b**) O1s narrow scan XPS spectrum. **c**) N1s narrow scan XPS spectrum. **d**) Mo3d narrow scan XPS spectrum.

**
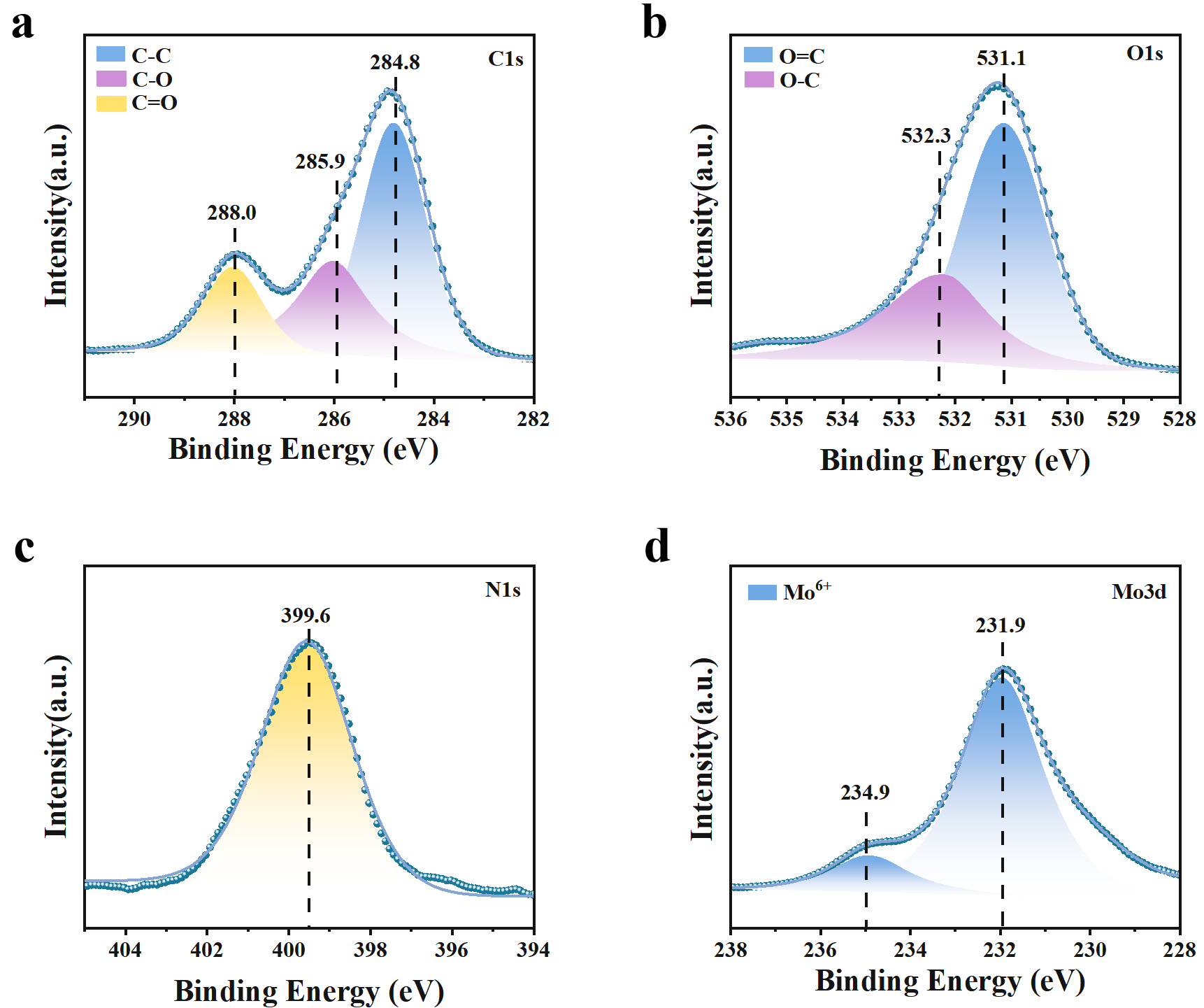
**

**Figure S9. XPS spectrum of TPM after reaction with O_2_^·-^. a)** C1s narrow scan XPS spectrum. **b**) O1s narrow scan XPS spectrum. **c**) N1s narrow scan XPS spectrum. **d**) Mo3d narrow scan XPS spectrum.

**
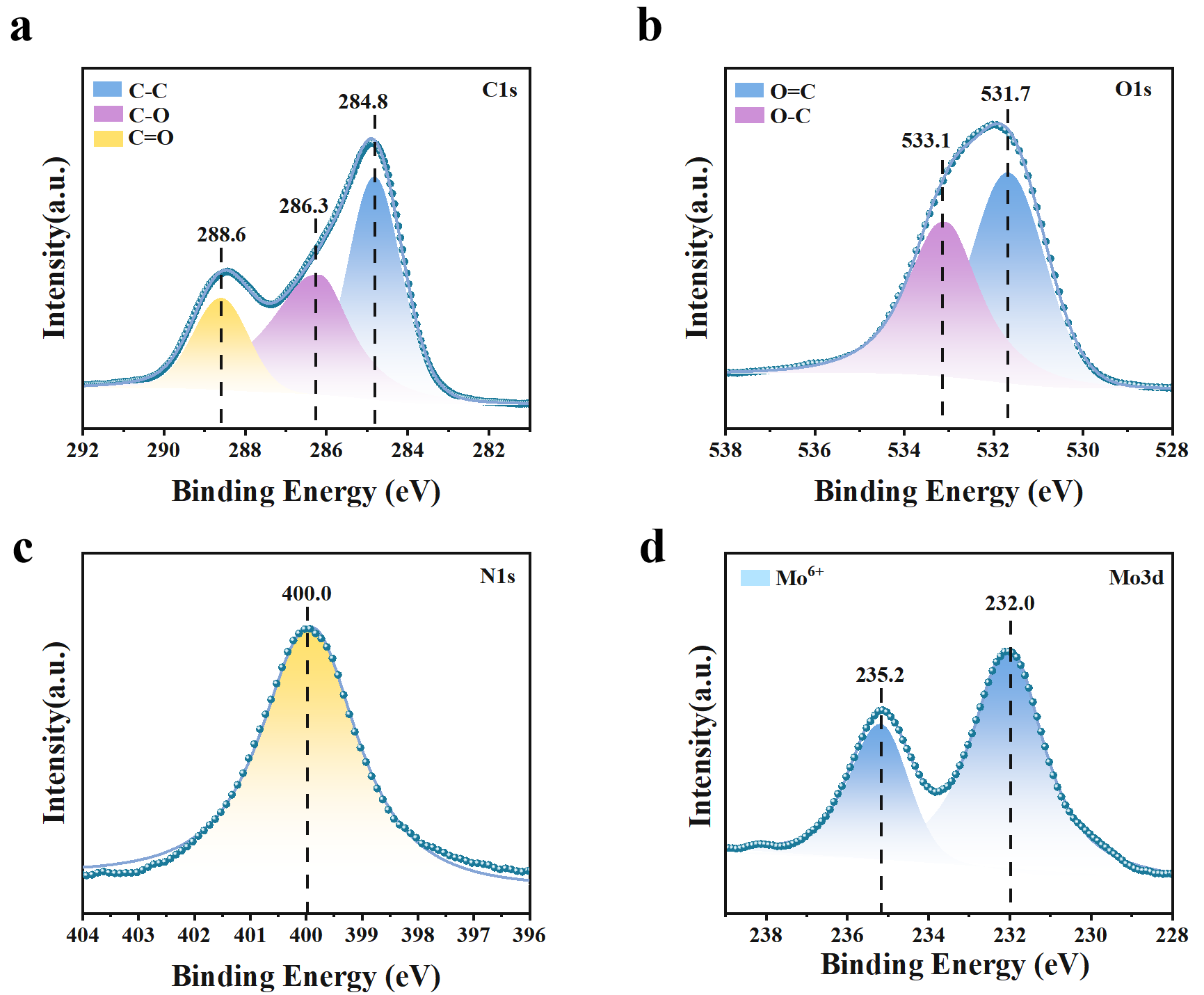
**

**Figure S10. XPS spectrum of TPM after reaction with ONOO^-^. a**) C1s narrow scan XPS spectrum. **b**) O1s narrow scan XPS spectrum. **c**) N1s narrow scan XPS spectrum. **d**) Mo3d narrow scan XPS spectrum.

**
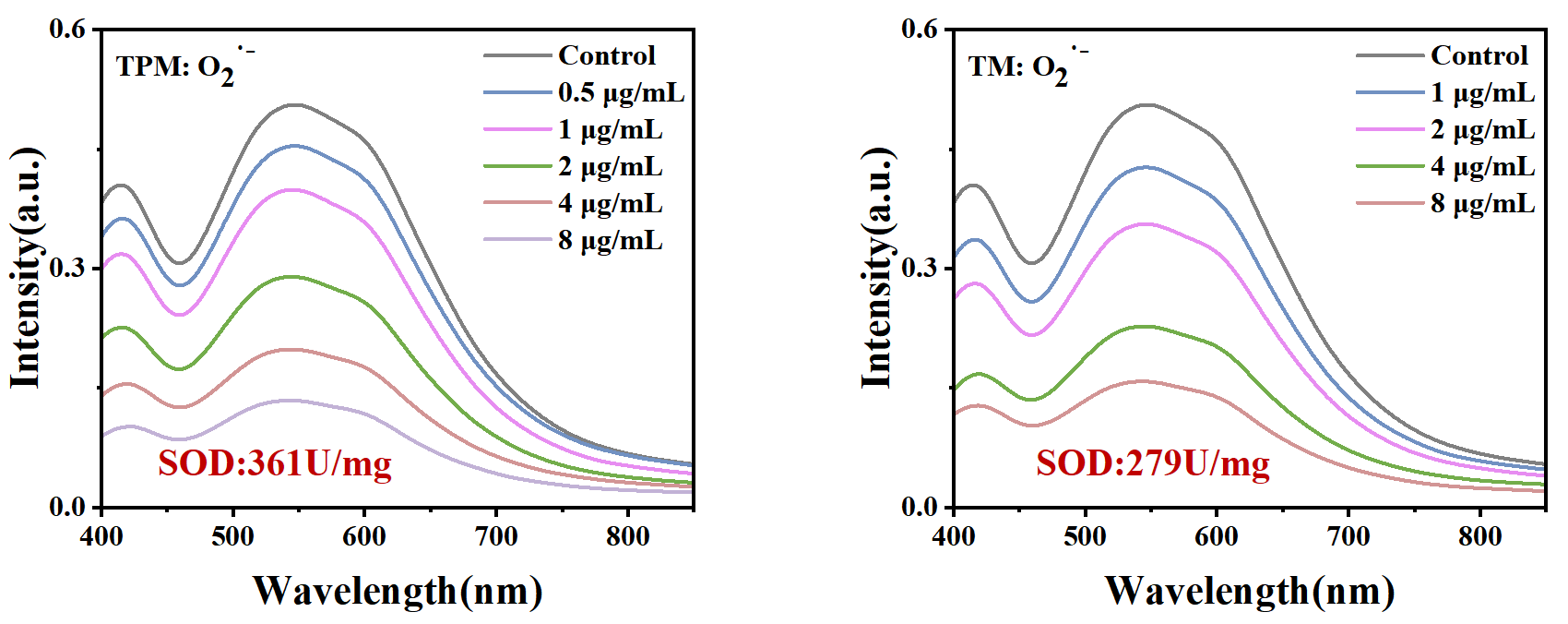
**

**Figure S11. In vitro O_2_^·-^ scavenging ability of TPM and TM.**

**
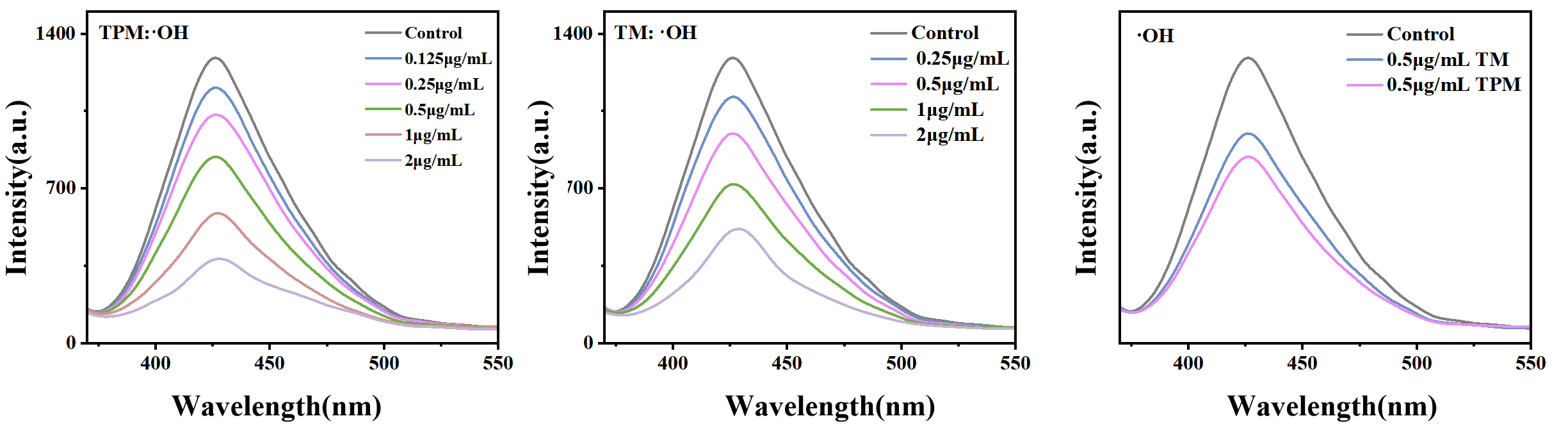
**

**Figure S12. In vitro ·OH scavenging ability of TPM and TM.**


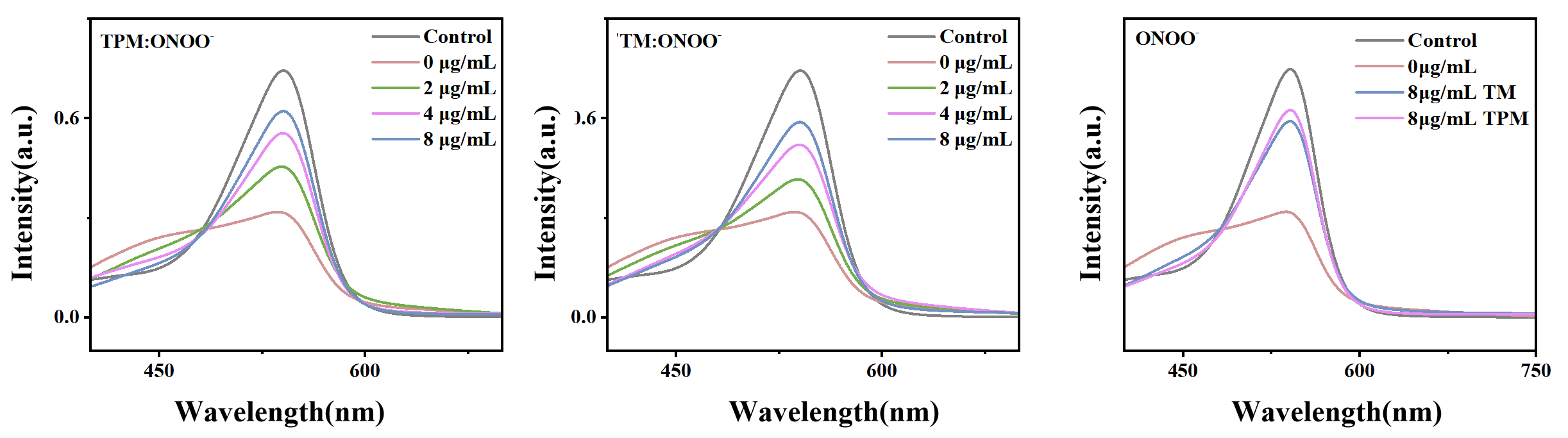


**Figure S13. In vitro ONOO^-^ scavenging ability of TPM and TM.**


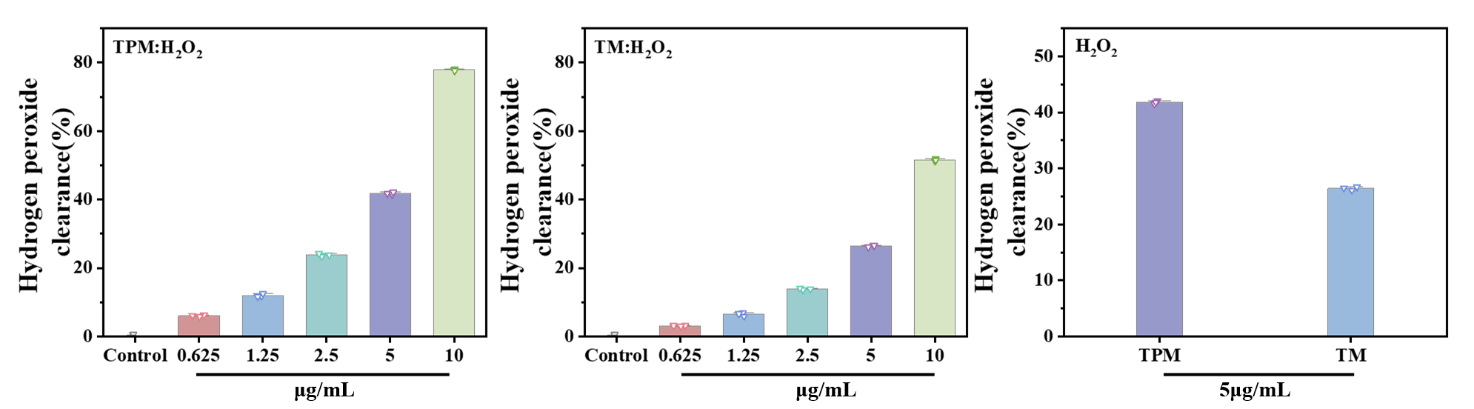


**Figure S14. In vitro H_2_O_2_ scavenging ability of TPM and TM.**

**
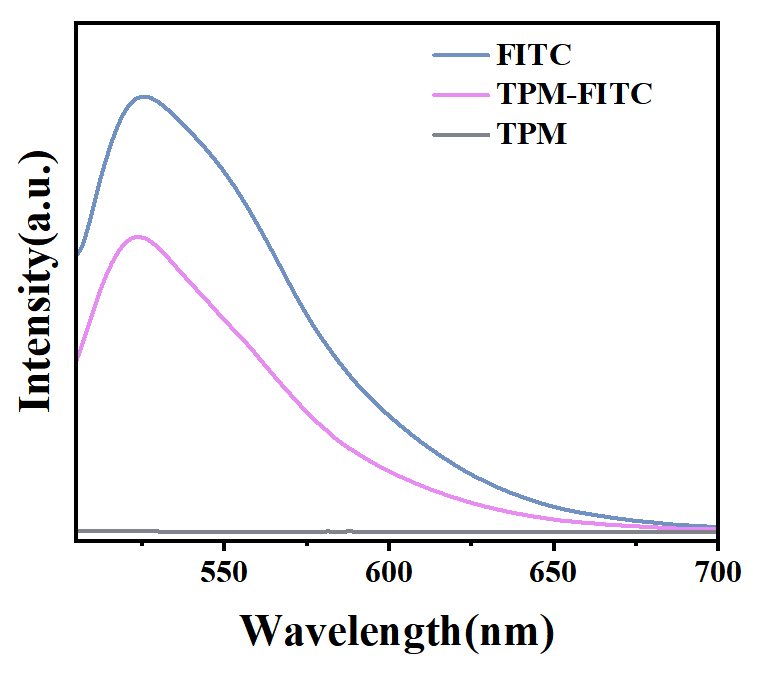
**

**Figure S15. In vitro fluorescence of FITC-labeled TPM.**


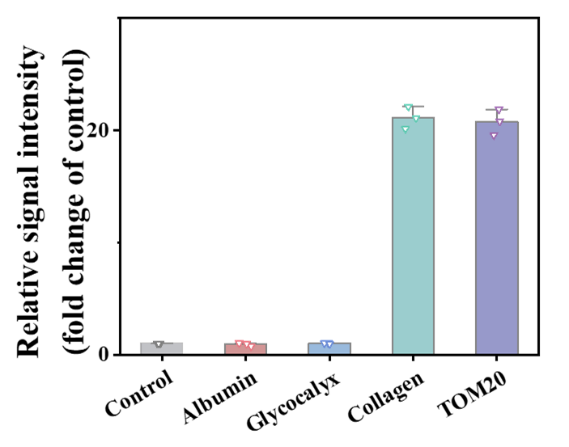


**Figure S16. Quantitative analysis of the protein-coated plate experiment demonstrated TPM could bind to collagen**

**
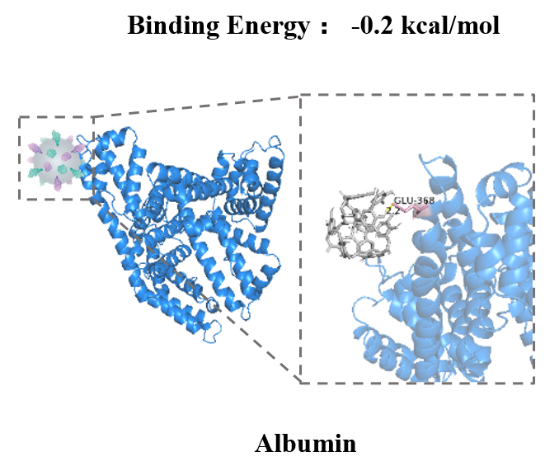
**

**Figure S17. Results of molecules docking of Tannic acid with Albumin protein.**

**
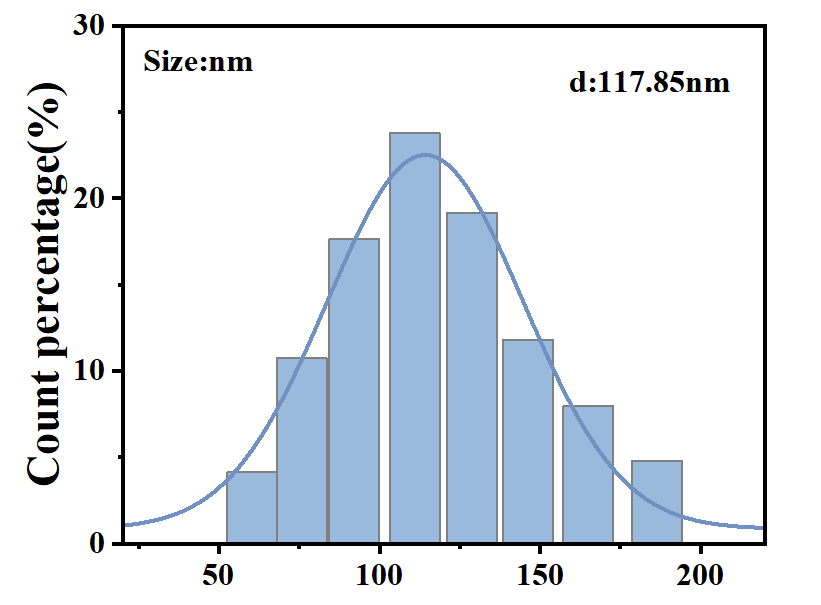
**

**Figure S18.** **Histogram of the size of endothelial cell gaps in infarcted brain tissue.**


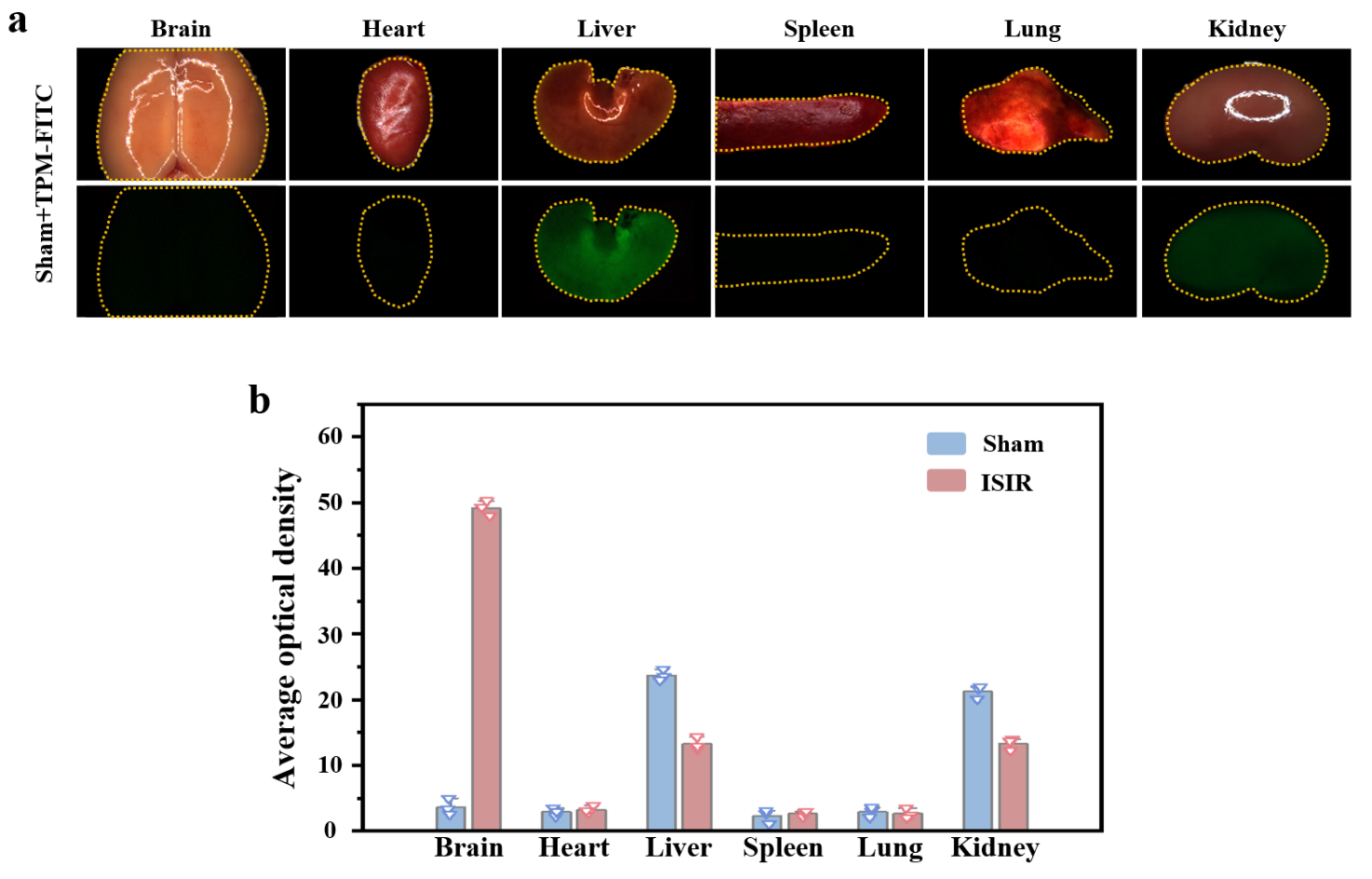


**Figure S19. Representative images of TPM-FITC fluorescence imaging and bright field in the brain, heart, lung, liver, spleen, and kidney of rats with Sham (a) and** **fluorescence imaging statistics of each organ (b).**


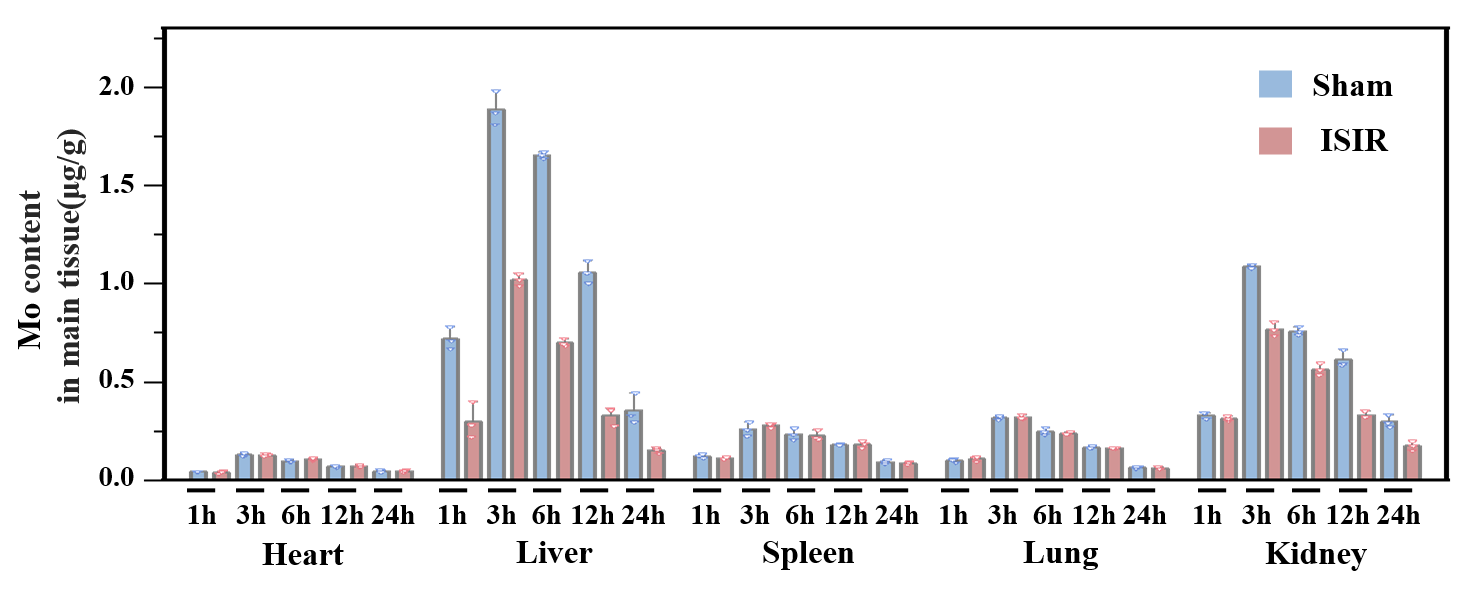


**Figure S20.** **ICP-MS measured the content of Mo in heart, liver, spleen, lung and kidney tissue after intravenous injection of TPM at 1h, 3h, 6h, 12h, and 24h.**

**
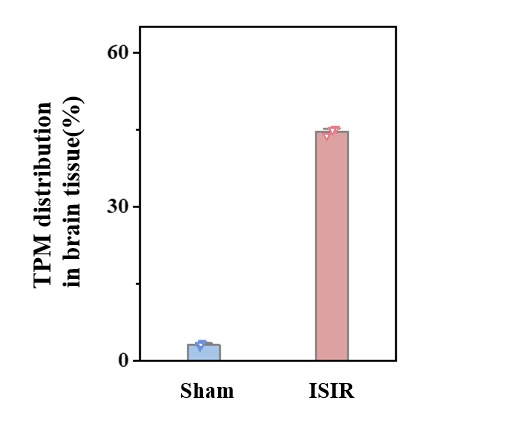
**

**
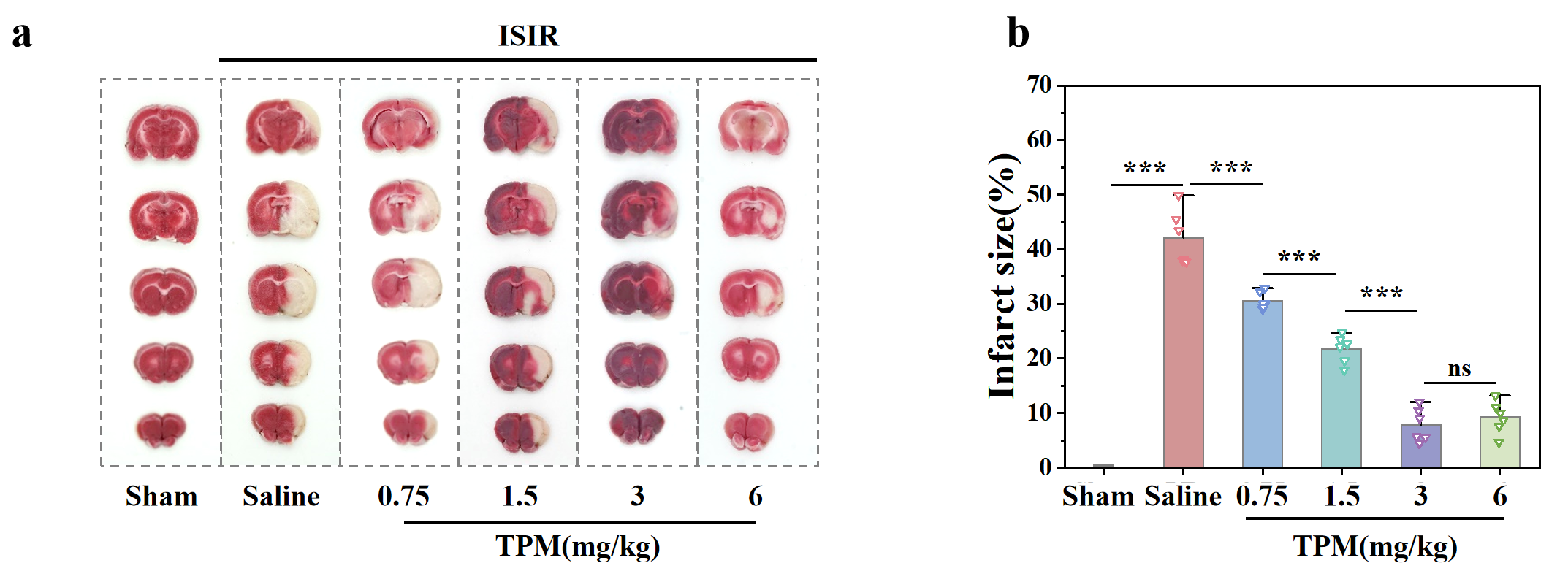
Figure S21. The distribution percentages of TPM in the brain tissue of the ISIR group were detected by ICP-MS.** (n = 3 animals per group).

**Figure S22. Brain tissue TTC staining(a) and infarct size statistics(b).** Data were expressed as mean ± SE. (n = 3, ns: *P* > 0.05, *^***^P* < 0.001)


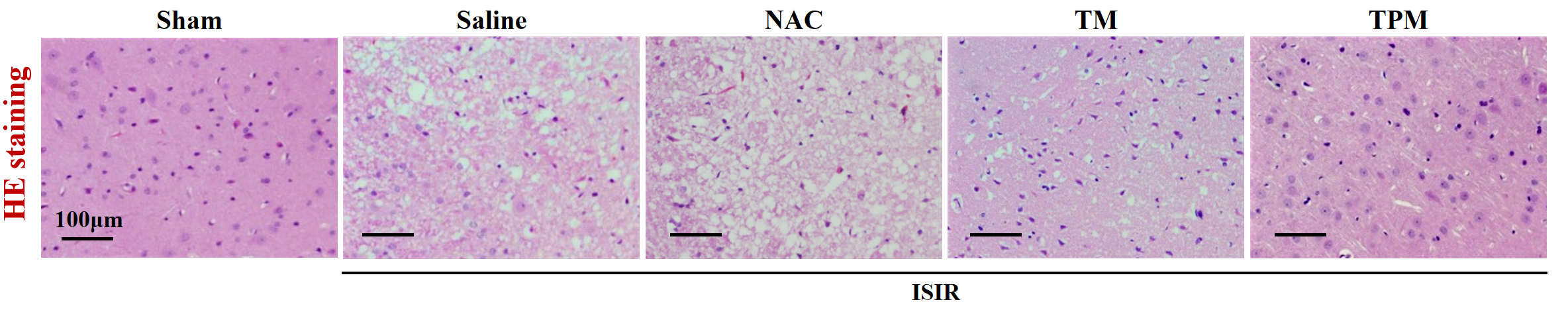


**Figure S23. HE staining explored the effects of TPM on brain tissue structure and neuronal function.** Scale bar: 100 μm.

**
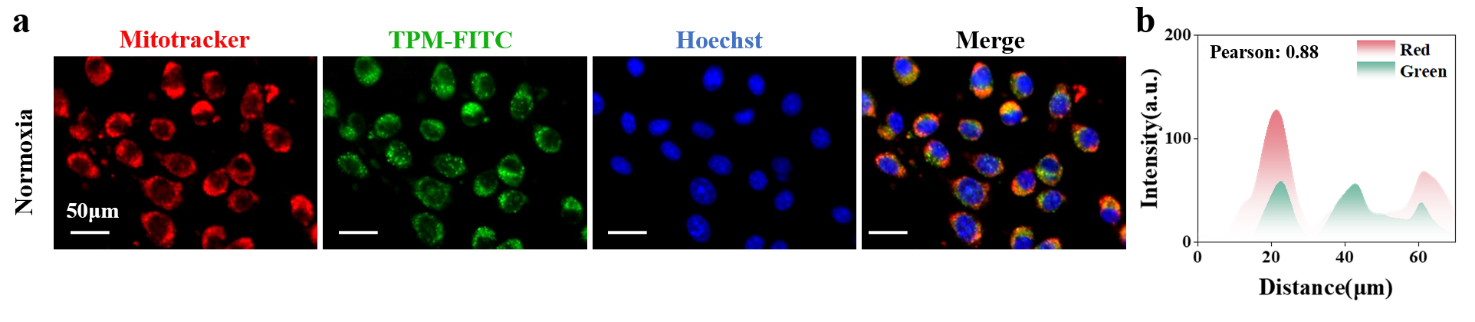
**

**Figure S24. Representative images and co-localization analysis of TPM-FITC and mitochondria (a) and Pearson coefficient analysis (b).** Scale bar: 50 μm.

**
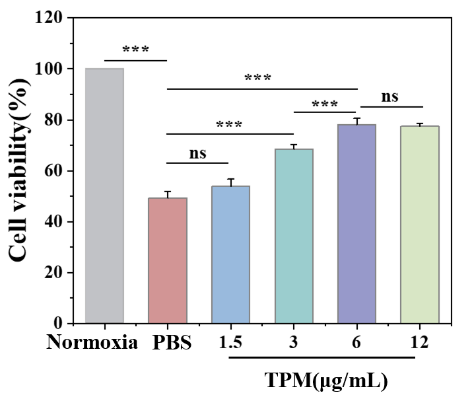
**

**Figure S25. The efficacy of TPM on HT22 cells induced by H/R.** Data were expressed as mean ± SE. (n = 3, ns *P* > 0.05, *^***^P* < 0.001)

**
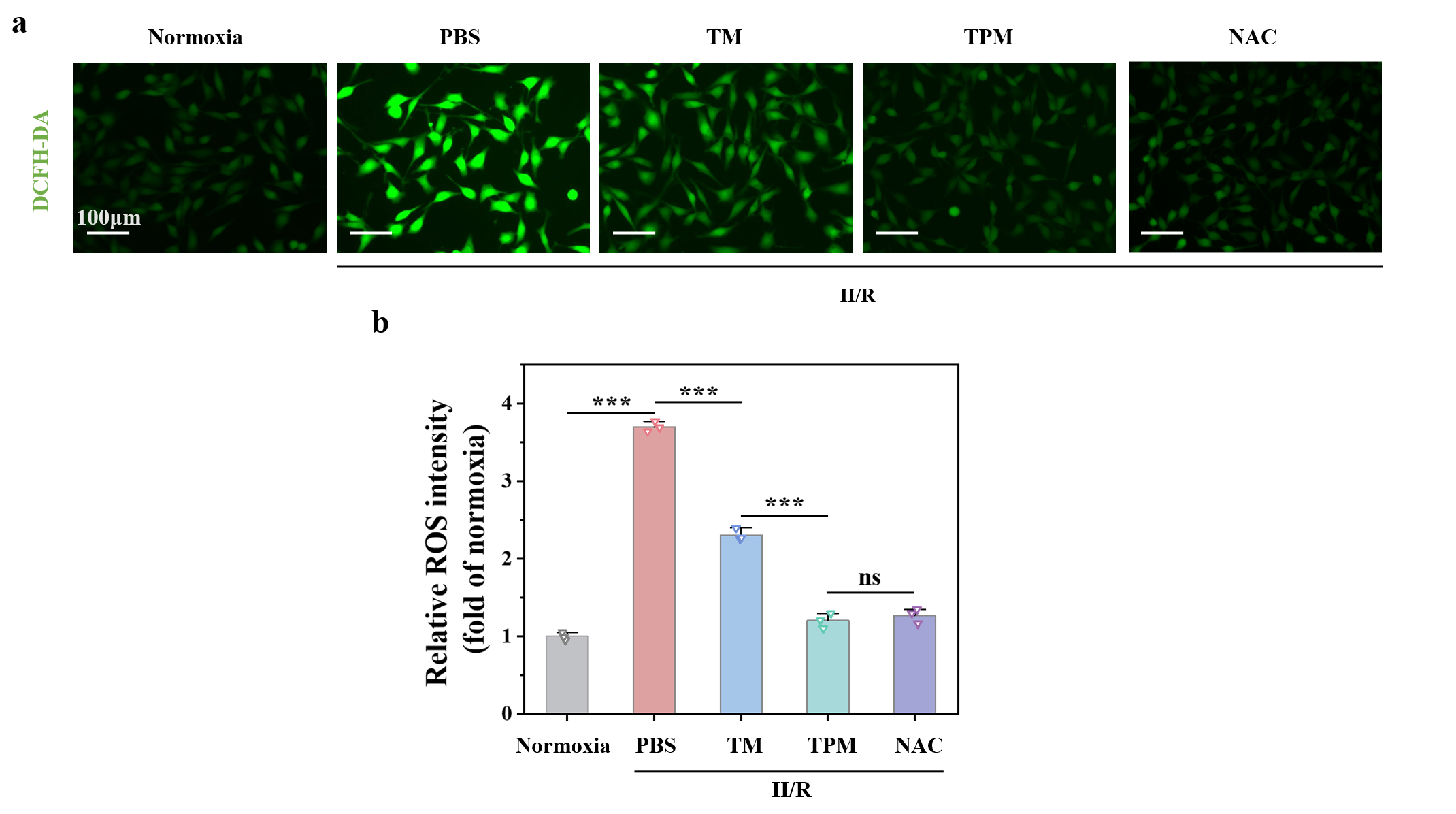
**

**Figure S26. Representative pictures of ROS in HT22 cells indicated by DCFH-DA (a) and quantitative analysis (b).** Scale bar: 100 μm. Data were expressed as mean ± SE. (n = 3, ns *P* > 0.05, *^**^P*< 0.01, *^***^P* < 0.001)

**
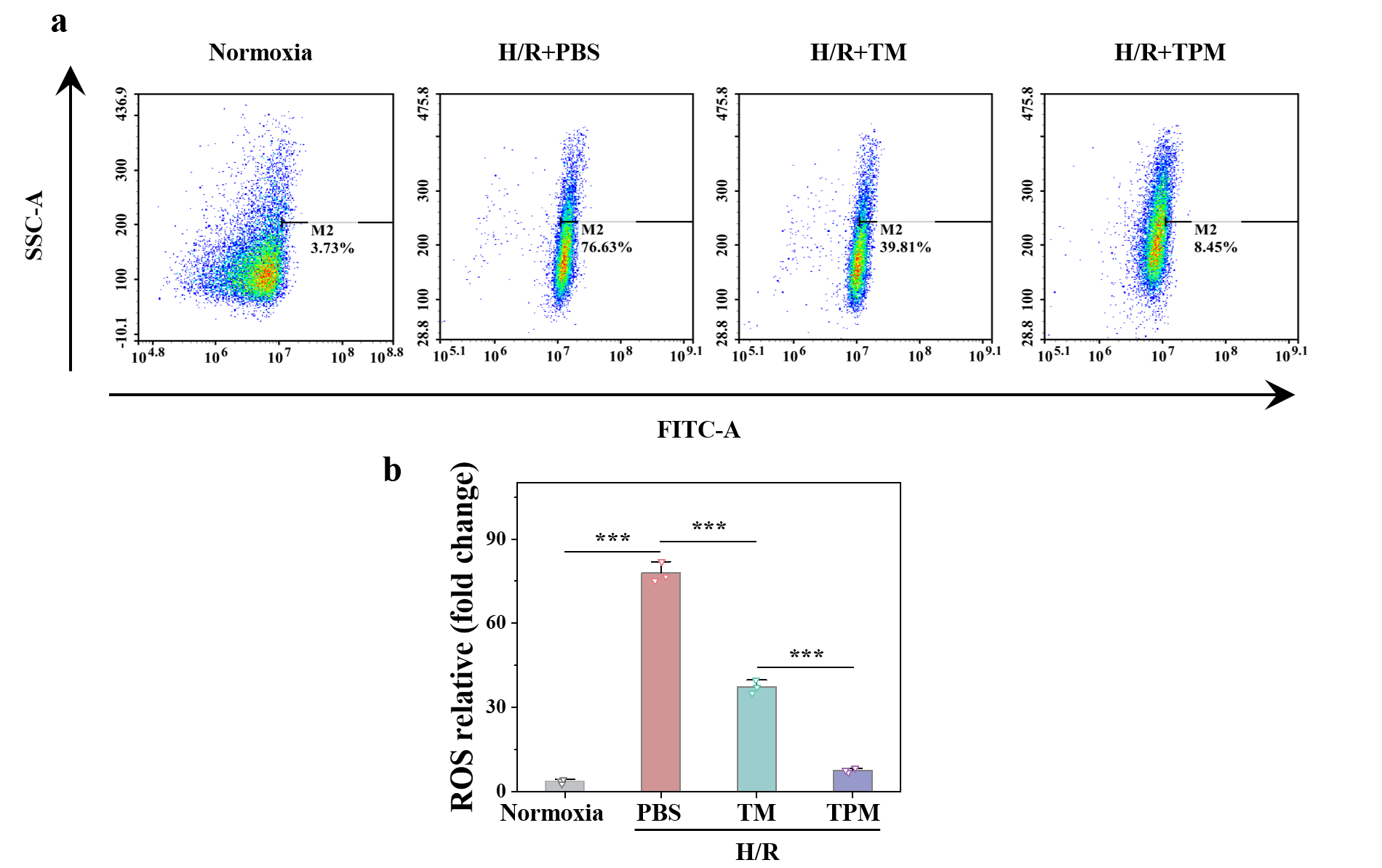
**

**Figure S27. Flow cytometric analysis of ROS activity in HT22 cells and quantification analysis of the relative mean fluorescence intensity.** Data were expressed as mean ± SE. (n = 3, *^***^P* < 0.001).


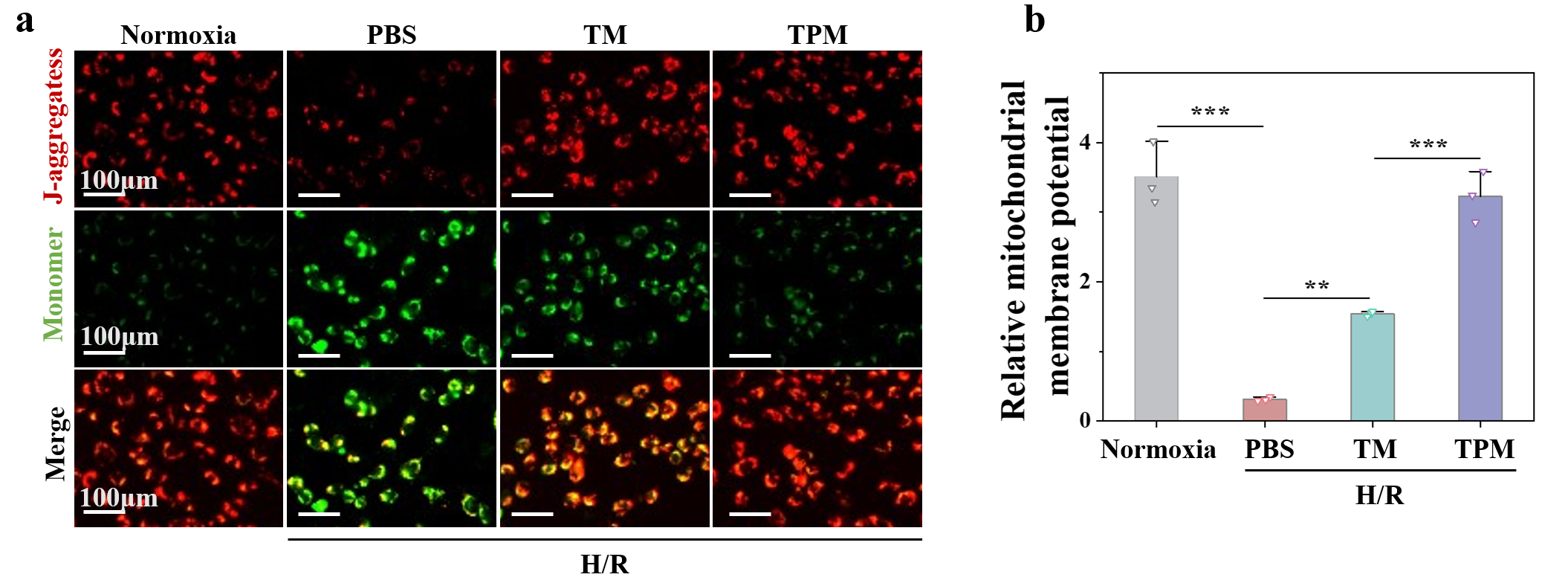


**Figure S28. Immunofluorescence staining of JC-1 and quantitative analysis in HT22 cells.** Scale bar: 100 μm. Data were expressed as mean ± SE. (n = 3, *^**^P* < 0.01, *^***^P* < 0.001).

**
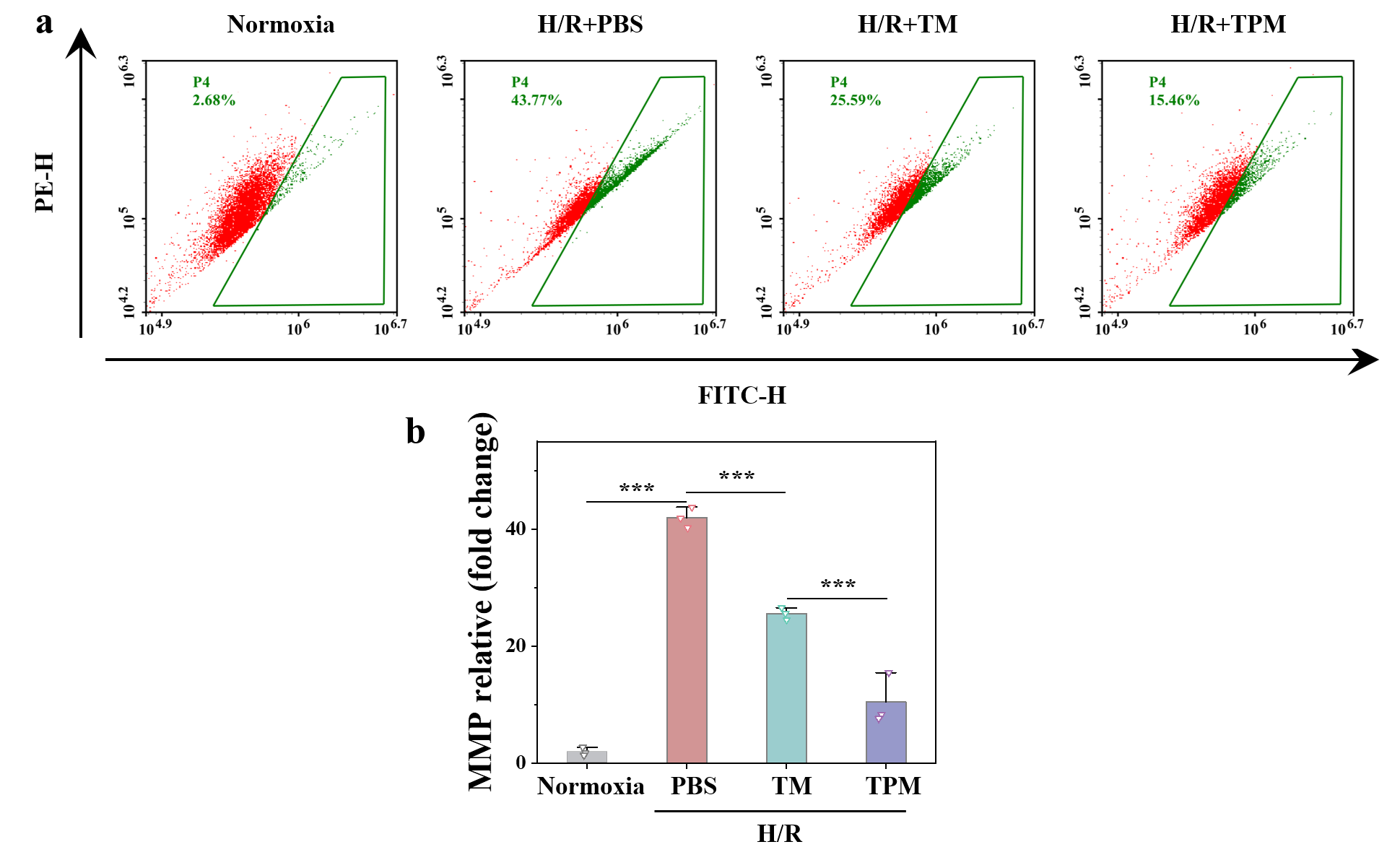
**

**Figure S29. Flow cytometric analysis of mitochondrial membrane potential (MMP) in HT22 cells and quantification analysis of the relative mean fluorescence intensity.** Data were expressed as mean ± SE. (n = 3, *^***^P* < 0.001).

**
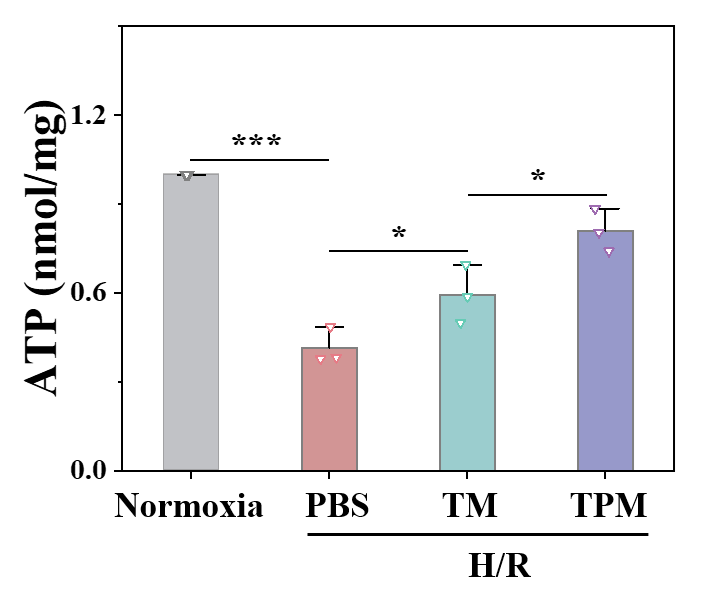
**

**
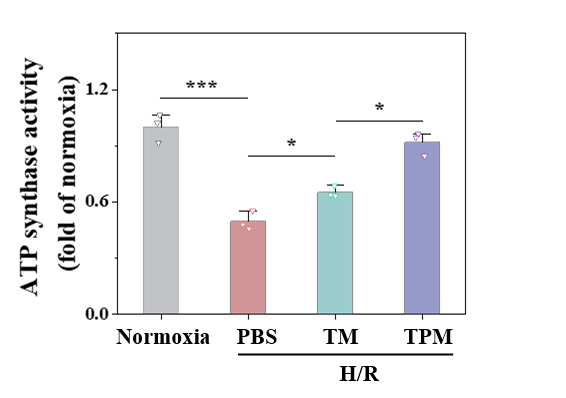
Figure S30. ATP production of HT22 cells under different treatments.** Data were expressed as mean ± SE. (n = 3, *^*^P* < 0.05, *^***^P* < 0.001)**.**

**Figure S31. ATP synthase activity of HT22 cells under different treatments.** Data were expressed as mean ± SE. (n = 3, **P* < 0.05, ****P* < 0.001)

**
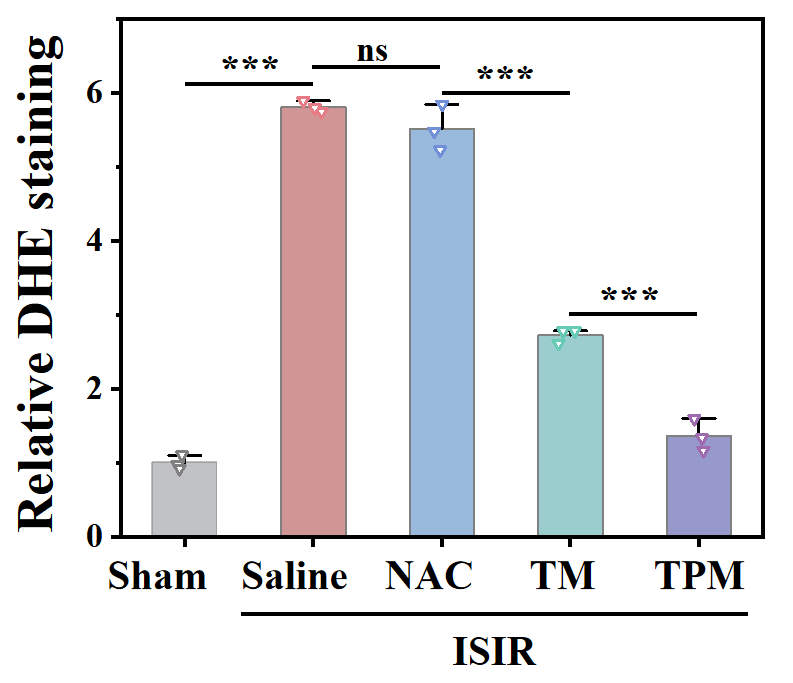
**

**Figure S32. Statistical results of DHE staining of brain tissue.** Data were expressed as mean ± SE. (n = 3, ns: *P* > 0.05, *^***^P* < 0.001)

**
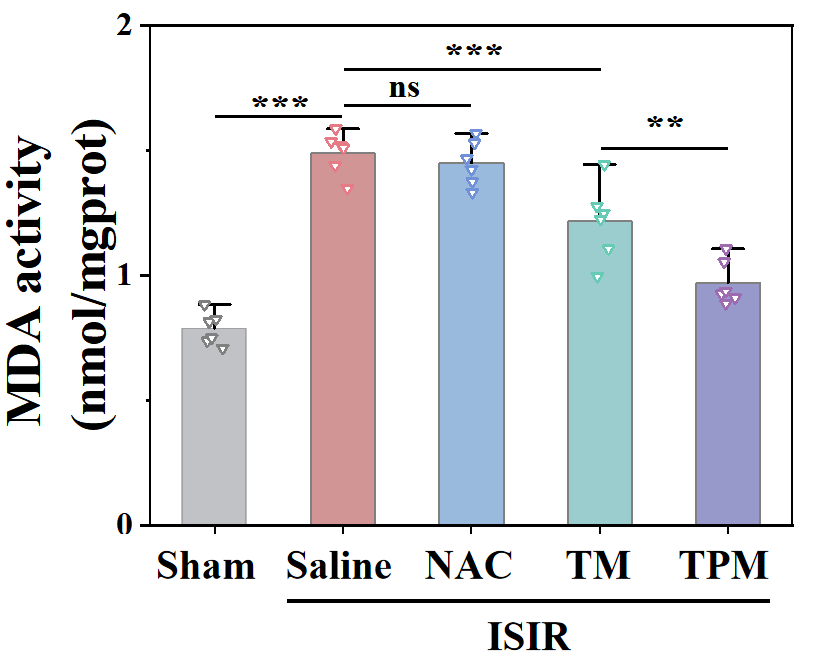
**

**Figure S33. Expression levels of MDA in different groups.** Data were expressed as mean ± SE. (n = 6, ns: *P* > 0.05, *^**^P* < 0.01, *^***^P* < 0.001)**.**

**
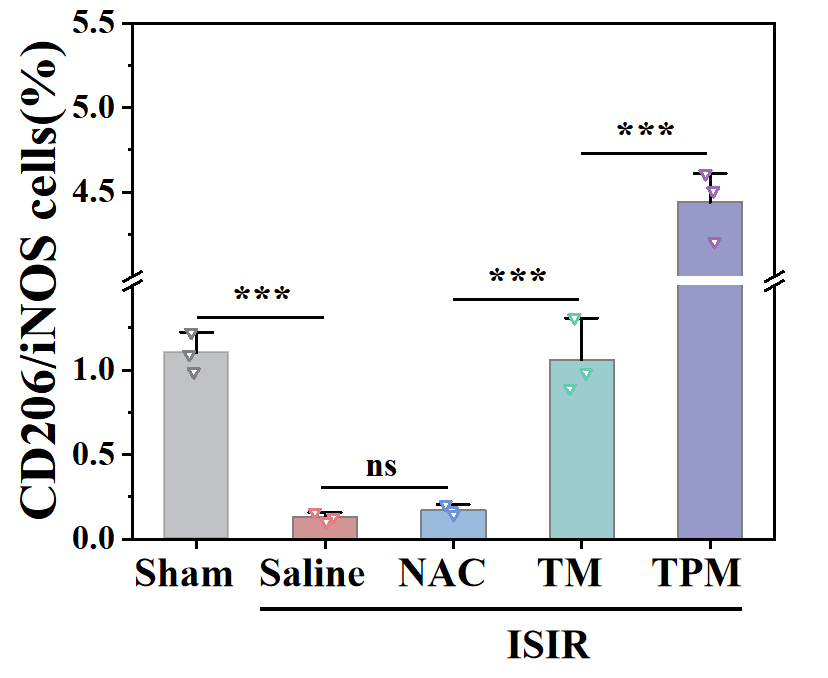
**

**Figure S34. Quantitative statistics of iNOS/CD206 fluorescence in brain tissue.** Data were expressed as mean ± SE. (n = 3, ns: *P*＞0.05, *^***^P* < 0.001**)**

**
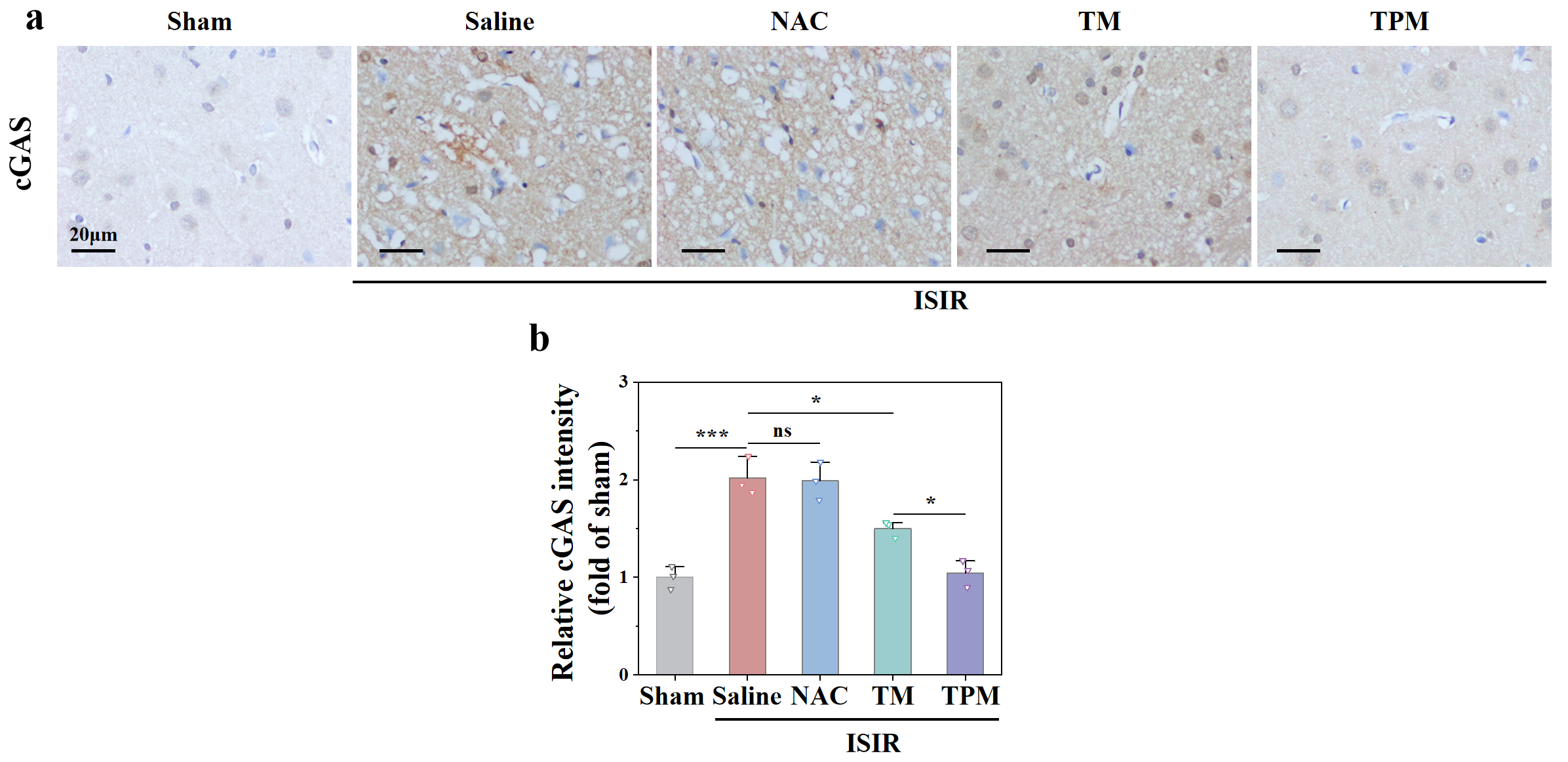
**

**Figure S35. Representative images (a) and quantitative analysis (b) of cGAS immunohistochemical staining in brain tissues of different treatment groups.** Scale bar: 20 μm. Data were expressed as mean ± SE. (n = 3, ns: *P*＞0.05, *^*^P* < 0.05, *^***^P* < 0.001**)**

**
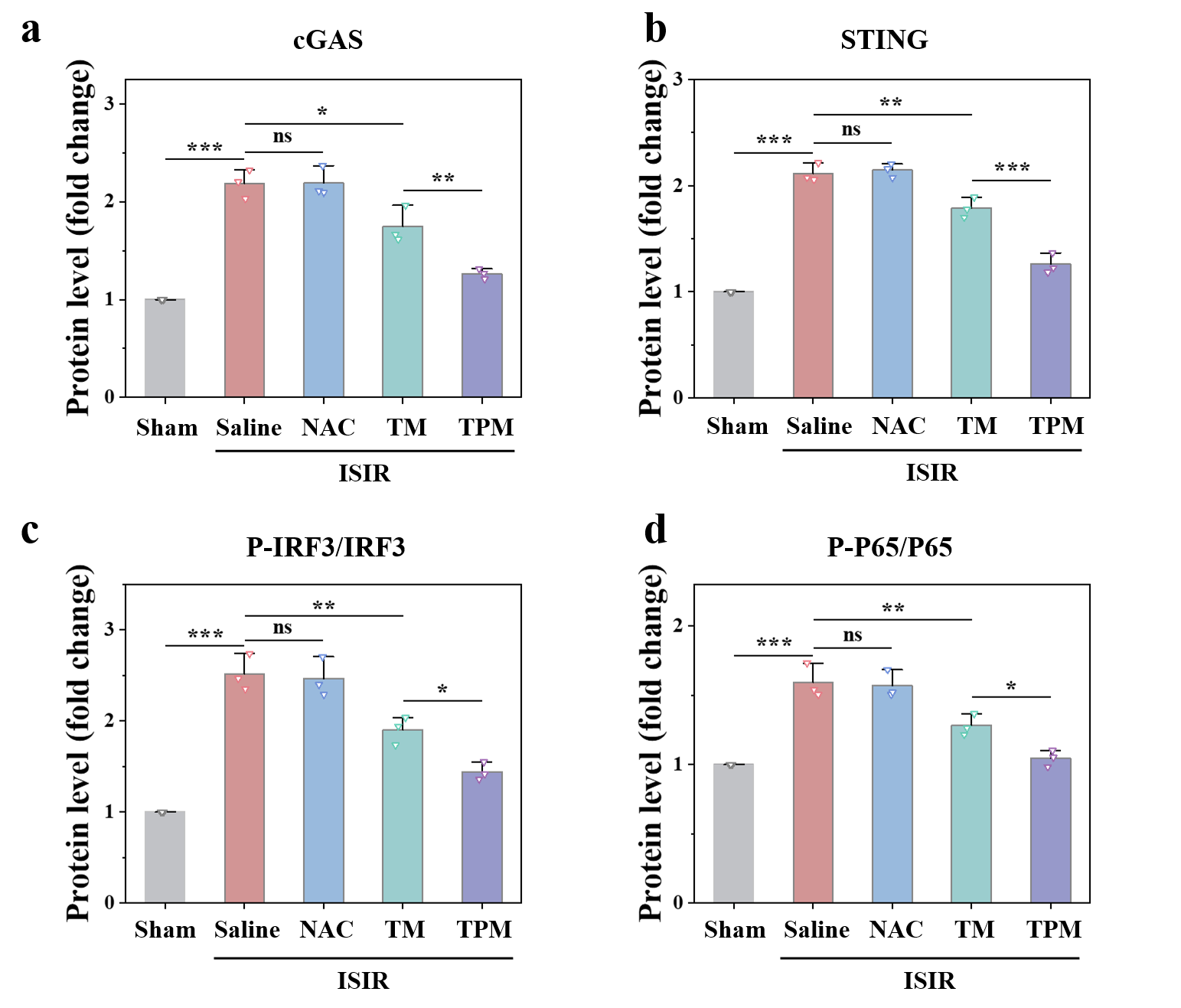
**

**Figure S36. Quantitative analysis of brain tissue inflammation-related proteins by WB. a**) cGAS, **b**) STING, **c**) P-IRF3/IRF3, **d**) P-P65/P65. Data were expressed as mean ± SE. (n = 3, ns: *P*＞0.05, *^*^P* < 0.05, *^**^P* < 0.01, *^***^P* < 0.001)


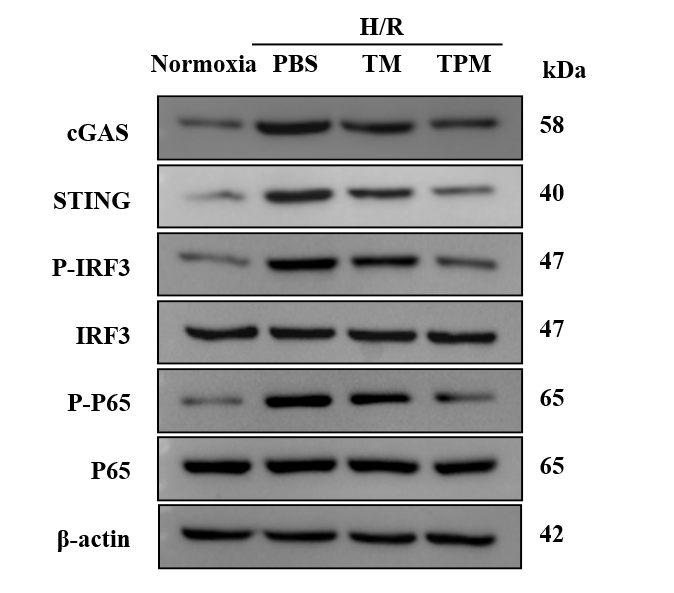


**Figure S37. WB representative image of inflammation-related proteins in BV2 cells.**

**
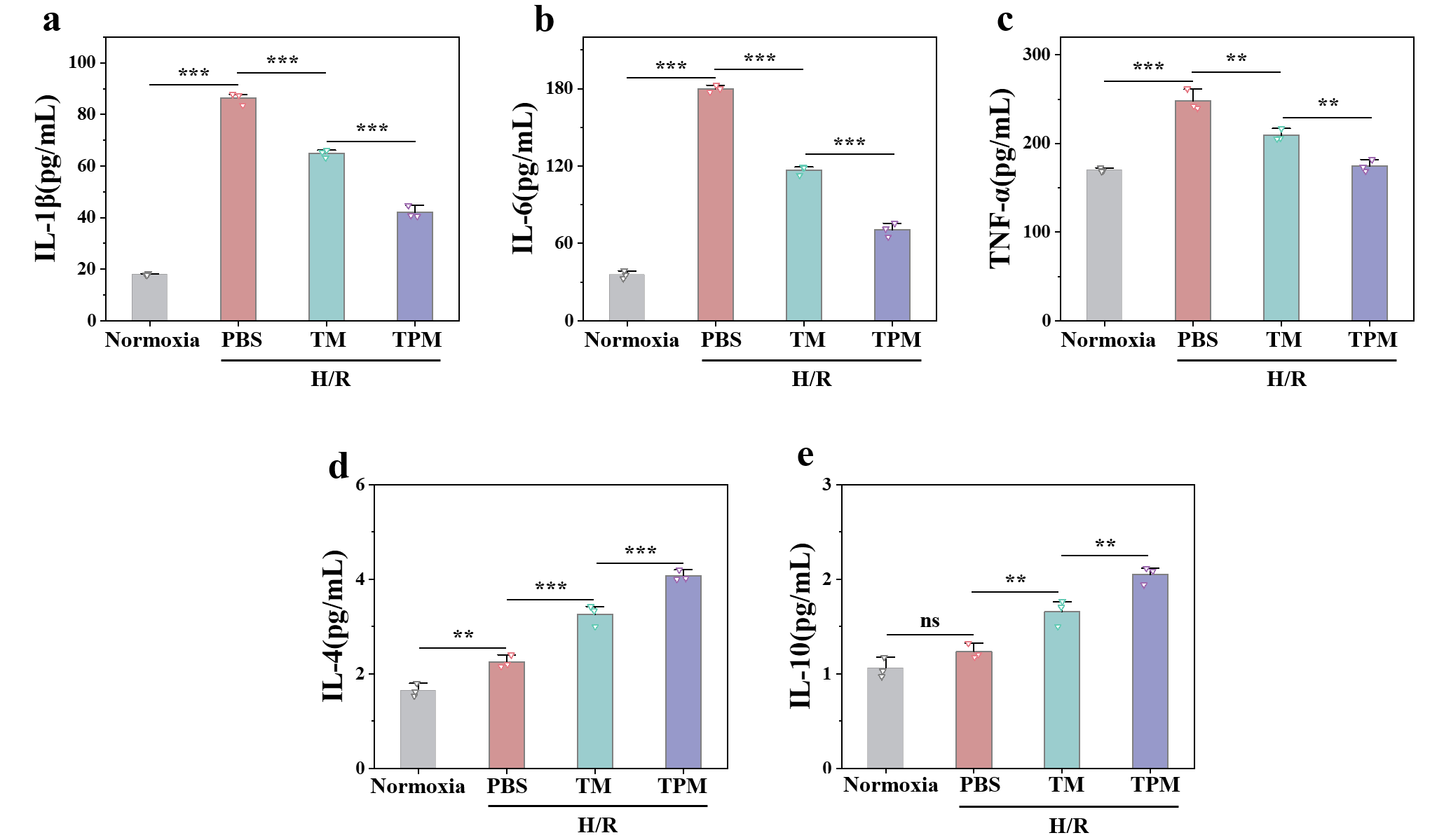
**

**Figure S38. BV2 cell inflammatory factor detection results. a**) IL-1β, **b**) IL-6, **c**) TNF-α, **d**) IL-4, **e**) IL-10. Data were expressed as mean ± SE. (n = 3, ns: *P*＞0.05, *^**^P* < 0.01, *^***^P* < 0.001).


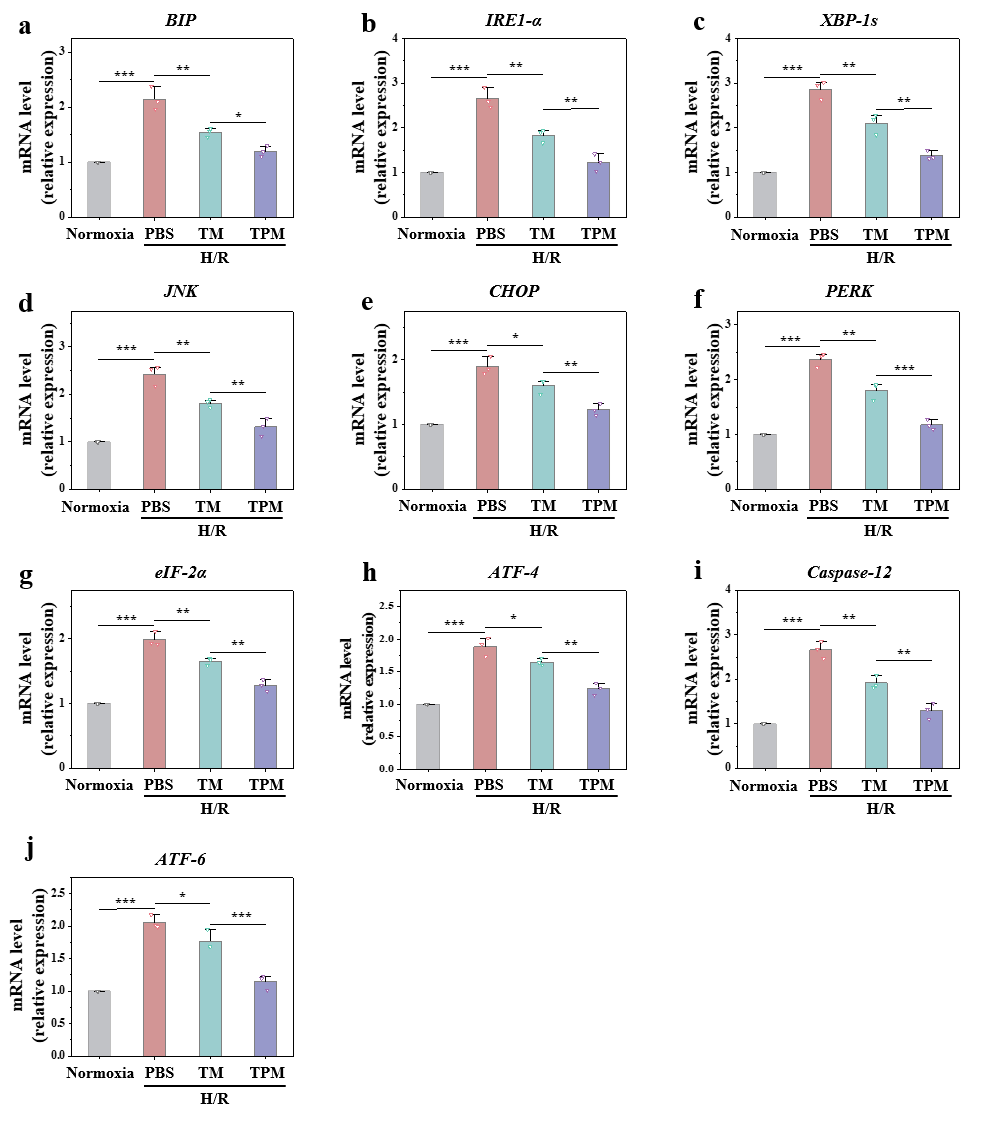


**Figure S39. qPCR detection of expression levels of ER stress-related factors in HT22 cells after different treatments. a**) *BIP*, **b**) *IRE1-α*, **c**) *XBP-1s*, **d**) *JNK,* **e**) *CHOP*, **f**) *PERK*, **g**) *eIF-2α*, **h**) *ATF-4*, **i**) *Caspase-12*, **j**) *ATF-6*. Data were expressed as mean ± SE. (n = 3, *^*^P* < 0.05, *^**^P* < 0.01, *^***^P* < 0.001)

**
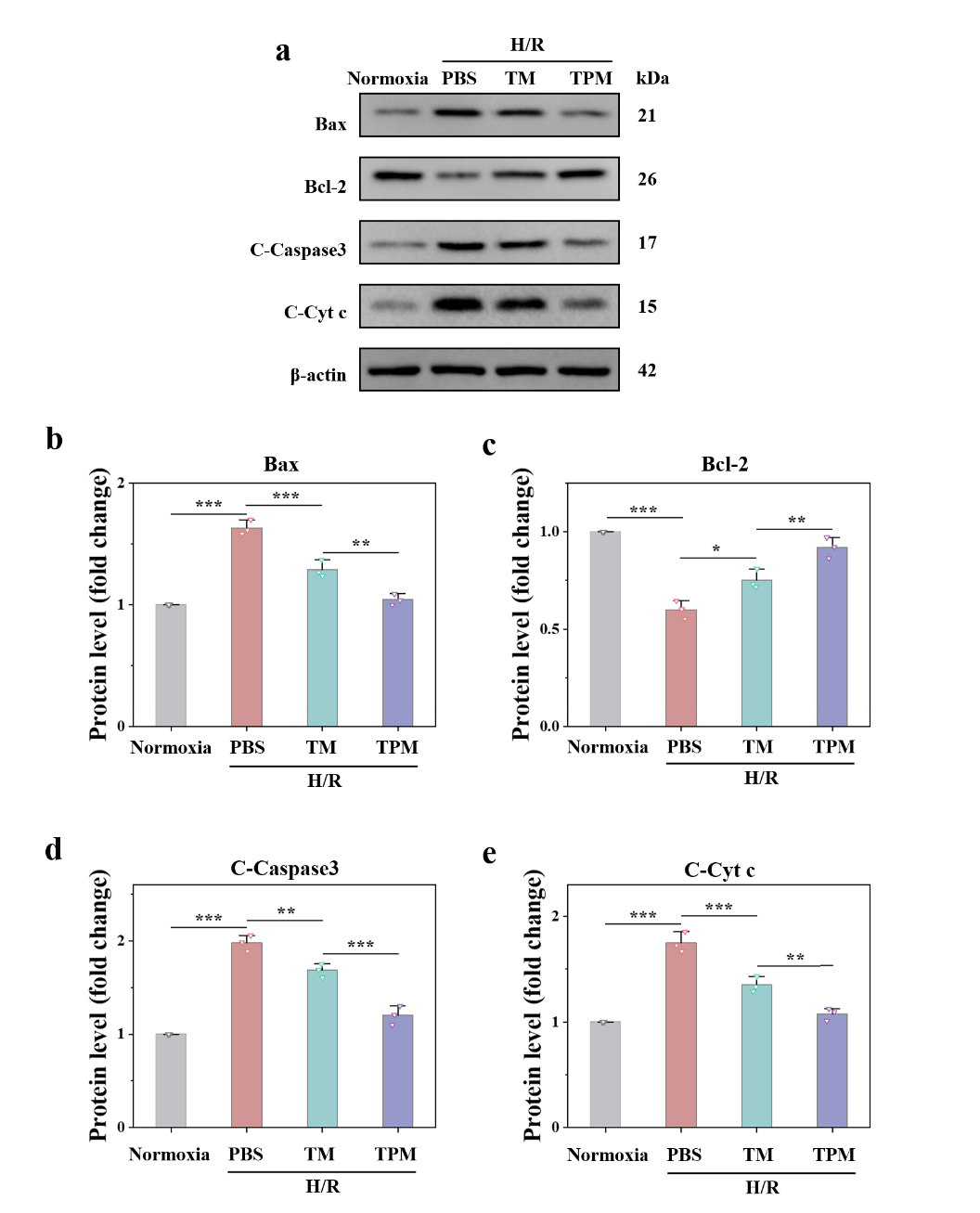
**

**Figure S40. WB representative image and quantitative analysis of apoptosis-related proteins in HT22 cells.** **a**) Representative image, **b**) Bax, **c**) Bcl-2, **d**) Cleaved-caspase3, **e**) Cytosolic-Cyt c. Data were expressed as mean ± SE. (n = 3, *^*^P* < 0.05, *^**^P* < 0.01, *^***^P* < 0.001)

**
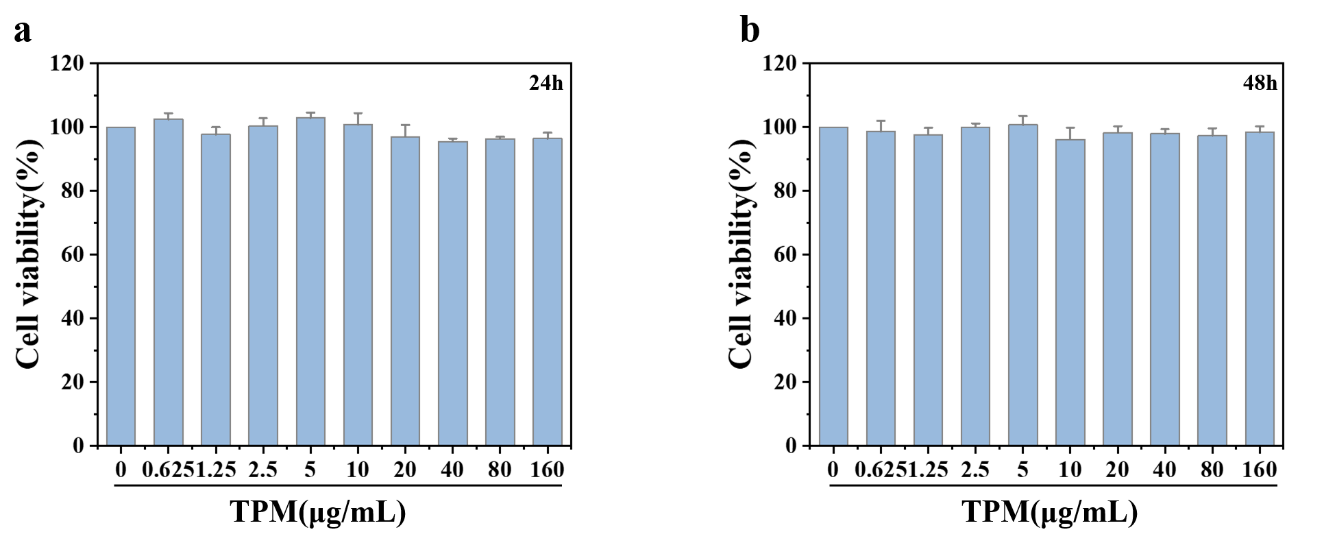
**

**Figure S41. After treating HT22 cells with different concentrations of TPM for 24h (a) and 48h (b), CCK8 detected cell viability results.**

**
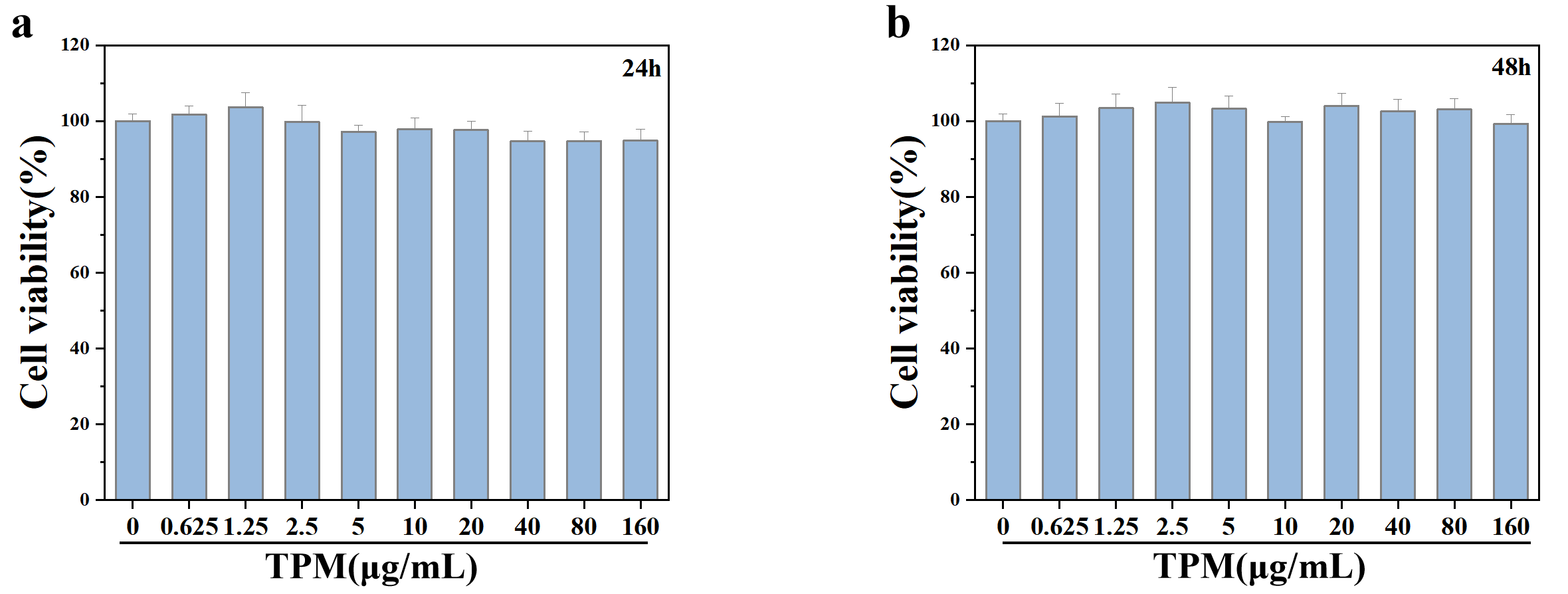
**

**Figure S42. After treating BV2 cells with different concentrations of TPM for 24h (a) and 48h (b), CCK8 detected cell viability results.**
